# Reactivity of a Unique Si(I)–Si(I)-Based η^2^-Bis(silylene) Iron Complex

**DOI:** 10.1021/acs.inorgchem.2c01369

**Published:** 2022-07-20

**Authors:** Zhiyuan He, Lingyu Liu, Felix J. de Zwart, Xiaolian Xue, Andreas W. Ehlers, KaKing Yan, Serhiy Demeshko, Jarl Ivar van der Vlugt, Bas de Bruin, Jeremy Krogman

**Affiliations:** † School of Physical Science and Technology, ShanghaiTech University, Shanghai 201210, China; ‡ van’t Hoff Institute for Molecular Sciences, University of Amsterdam, Science Park 904, 1098 XH Amsterdam, The Netherlands; § Institute of Chemistry, Carl von Ossietzky University, Carl-von-Ossietzky-Straße 9-11, 12629 Oldenburg, Germany; ∥ Department of Chemistry, University of Johannesburg, Auckland Park, Johannesburg P.O. Box 254, ZA-2006, South Africa; ⊥ Department of Chemistry, Georg August University, Tammanstraße 4, 37077 Göttingen, Germany

## Abstract

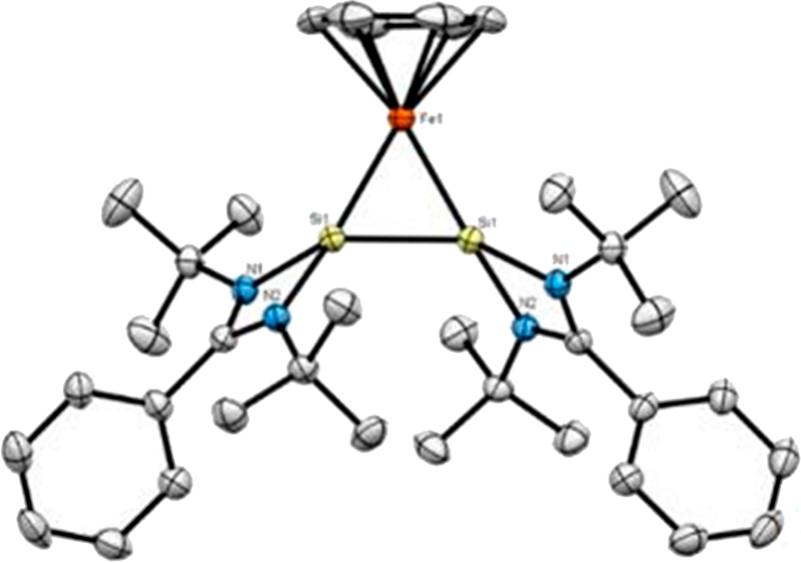

In this paper, we report the synthesis of a unique silicon(I)-based metalla-disilirane and report on its reactivity toward TMS-azide and benzophenone. Metal complexes containing disilylenes ((bis)silylenes with a Si–Si bond) are known, but direct ligation of the Si(I) centers to transition metals always generated dinuclear species. To overcome this problem, we targeted the formation of a mononuclear iron(0)–silicon(I)-based disilylene complex via templated synthesis, starting with ligation of two Si(II) centers to iron(II), followed by a two-step reduction. The DFT structure of the resulting η^2^-disilylene-iron complex reveals metal-to-silicon π-back donation and a delocalized three-center–two-electron (3c–2e) aromatic system. The Si(I)–Si(I) bond displays unusual but well-defined reactivity. With TMS-azide, both the initial azide adduct and the follow-up four-membered nitrene complex could be isolated. Reaction with benzophenone led to selective 1,4-addition into the Si–Si bond. This work reveals that selective reactions of Si(I)–Si(I) bonds are made possible by metal ligation.

## Abstract 2

The first selective ligation of a silicon(I)-based disilylene to a mononuclear metal center is achieved via templated synthesis. Computational analysis of the ensuing η^2^-disilylene-iron(0) complex supports a delocalized three-center−two-electron (3c−2e) aromatic system. The Si(I)−Si(I) bond displays well-defined reactivity toward trimethylsilyl azide and benzophenone. This work reveals that selective reactions of Si(I)−Si(I) bonds are made possible by metal ligation.

## Introduction

**Scheme 1:**
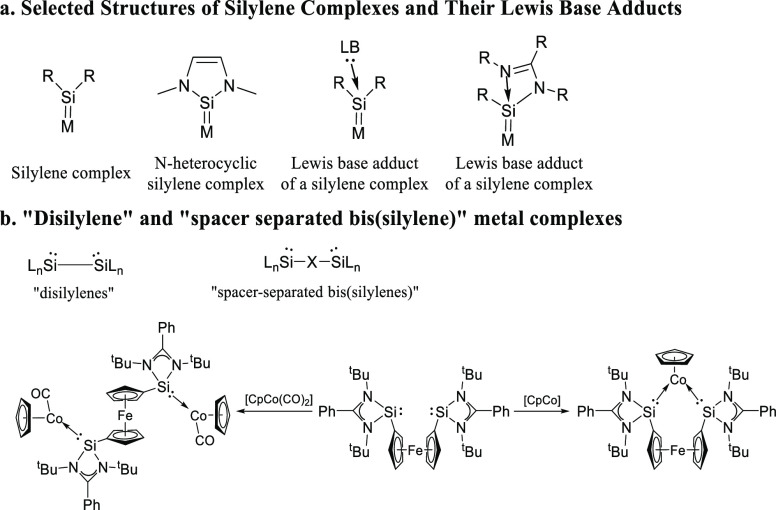
(a) Selection of Different Monosilylenes; (b) Difference between “Disilylene” and “Spacer-Separated Bis(silylene)” and Examples of Metal Complexes of the Latter

Transition metal silylene complexes (Scheme 1a) have attracted significant interest as they have shown interesting (electronic) structures and reactivity, giving rise to synthetic and catalytic applications that differ significantly from transition metal carbene complexes.^1^ Several variants of transition metal silylene complexes have been reported, varying in the oxidation state of the silicon atom and the types of substituents, and among those variants, *N*-heterocyclic silylenes have been studied most extensively.^2^

Two types of the general class of bis(silylene) compounds have been reported: (1) “bis(silylenes) with a direct Si–Si bond”, wherein the two divalent silicon atoms are adjacent to each other and are connected by a central Si–Si bond (like others in the field,^3^ we term these “disilylenes”); (2) “spacer-separated bis(silylenes)”, with the two divalent silicon atoms separated by a spacer.^4^ Driess and co-workers recently reported a ferrocene-separated bis(silylene) acting as a bidentate ligand in catalytically active mono- and dicobalt complexes (Scheme 1b).^4e^

**Scheme 2:**
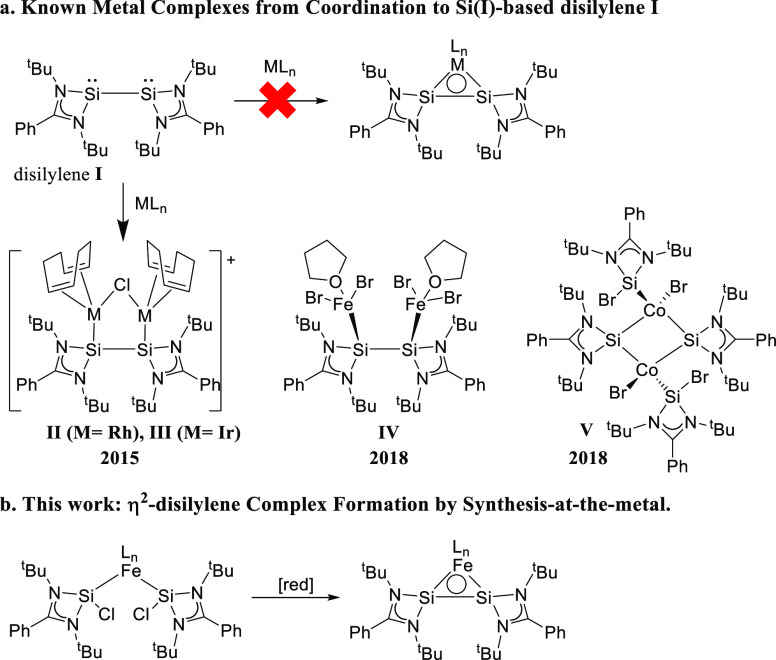
(a) Reported Di- and Multinuclear Disilylene Transition Metal Complexes; (b) Synthesis and Reactivity of a Mononuclear η^2^-Disilylene Iron Complex (This Work)

We consider “disilylenes” to be particularly interesting due to the potential reactivity associated with their Si–Si bond. Compound **I**, with each Si center bearing an amidinate ligand (Scheme 2), was synthesized by Roesky and co-workers,^5^ while Jones *et al*. reported a bulkier derivative thereof.^6^ Additionally, “disilylenes” that are stabilized by an *N*-heterocyclic carbene (NHC) or an intramolecular phosphine are also reported.^4a,7^ Targeting the synthesis of η^2^-coordinated Si(I)-based “disilylene” complexes seems particularly useful because the altered electronic structure and the lability of the Si–Si bond induced by η^2^-coordination are expected to give rise to unique reactivity.

Thus far, the reported reactivity of these platforms predominantly involves the Si-centered lone pairs rather than the Si–Si σ-bond. For example, So *et al.* reported the formation of the bimetallic disilylene Rh and Ir complexes **II** and **III** (Scheme 2a).^8^ Also, the dinuclear iron(II)bromide complex **IV** is formed cleanly, but the Si–Si bond is cleaved by cobalt(II)bromide to form dinuclear cobalt(bromosilylene)(silyl) complex **V**.^9^ To the best of our knowledge, no mononuclear metal adducts of ligand **I** are reported to date.

**Scheme 3:**
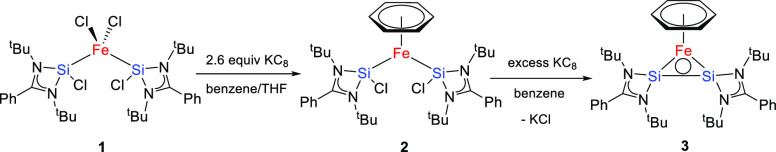
Synthetic Procedure for the Preparation of Complex **3**

To achieve η^2^-coordination of **I** to a mononuclear transition metal complex, we decided to explore the reductive coupling of two Si(II)-chlorides in the coordination sphere of a metal center (Scheme 2b). We herein describe iron complex **3** as the first η^2^-disilylene complex prepared by this protocol (Scheme 3). This species has an unusual electronic structure, with the Si–Si bond acting as a π-acceptor moiety, while the three-membered Fe–Si–Si ring shows 2π-aromaticity. It also displays selective Si–Si bond-centered reactivity toward azide activation and ketone addition, giving access to new “spacer-separated bis(silylenes)”.

## Results and Discussion

### Synthesis of **3**

**Figure 1:**
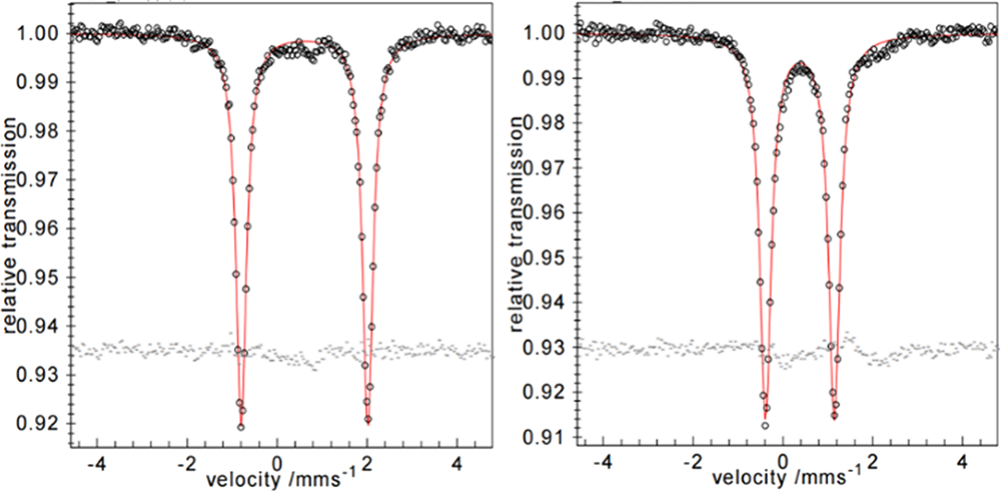
Mössbauer spectra of compounds **1** (left) and **2** (right) at 80 K.

The amidinate-stabilized silylene iron-halide precursor **1**, synthesized from Fe{N(SiMe_3_)_2_}_2_ using a literature procedure,^10^ was characterized with zero-field ^57^Fe Mössbauer spectroscopy (Figure 1). The isomer shift δ (0.61 m/s) and the quadrupole splitting Δ*E*_Q_ (2.81 mm/s) are in agreement with a four-coordinated Fe(II) center.^11^ Iron-centered reduction of this well-defined Fe(II) species with a slight excess of KC_8_ in a benzene-THF mixture (1:1 v/v%) generated bis-silylene compound **2**, featuring an η^6^-benzene fragment bound to Fe(0).

The ^1^H NMR spectrum of diamagnetic **2** in deuterated benzene exhibits a chemical shift for the η^6^-benzene fragment at δ 5.15 ppm, which is similar to that seen for the previously reported zero-valent iron complex [(SiFcSi)Fe-η^6^(C_6_H_6_)] bearing a ferrocene-bridged bis(silylene) ligand (δ 5.16 ppm).^12^ The ^29^Si NMR spectrum of **2** exhibits two sharp singlets at δ 45.12 and 42.45 ppm (suggesting chemically inequivalent Si centers), which are close to the reported chemical shift (δ 43.10 ppm) for an *N*-heterocyclic silylene iron(0) complex.^13^ Species **2** was also examined using zero-field ^57^Fe Mössbauer spectroscopy to confirm the oxidation state of Fe (Figure 1). Indeed, both the isomer shift δ (0.38 m/s) and quadrupole splitting Δ*E*_Q_ (1.53 mm/s) support reduction to Fe(0).^14^

**Figure 2:**
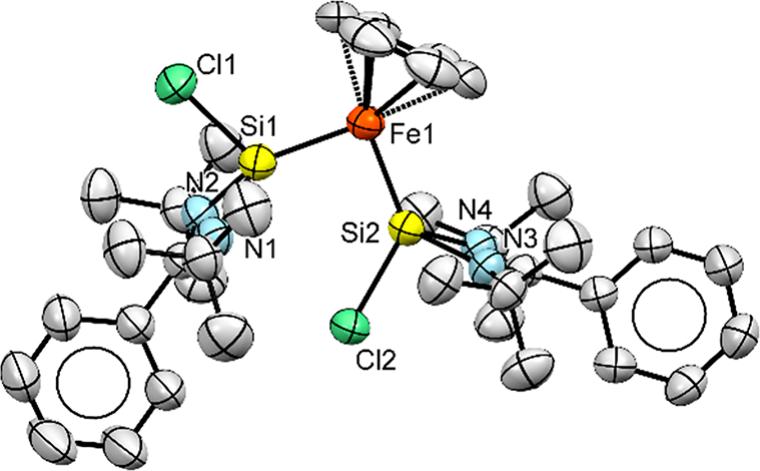
Molecular structure of **2** with thermal ellipsoids drawn at 30% probability. Hydrogen atoms are omitted for clarity. Selected bond lengths (Å) and angles (°): Si1–Fe1, 2.162(2); Si2–Fe1, 2.148(1); Si1–N1, 1.860(5); Si1–N2, 1.866(5); Si1–Si2, 3.100(2); Si1–Fe1–Si2, 91.99(6).

Single crystals of **2** that were obtained from pentane at −30 °C proved suitable for crystallographic analysis by X-ray diffraction at room temperature; loss of crystallinity was observed at 150 K. The molecular structure (Figure 2) shows two different orientations for the Si–Cl bonds with respect to the Fe(η^6^-benzene) fragment (one “up” and one “down”), which explains the presence of two chemically inequivalent Si nuclei in the ^29^Si NMR spectrum. The Si–Fe–Si angle is nearly 90° (91.99°), and the intramolecular Si1···Si2 distance is 3.100(2) Å. The Si–Fe1 bond lengths are 2.162(2) Å (Si1) and 2.148(1) Å (Si2), which are significantly shorter than the corresponding bond lengths in precursor **1** (∼2.445 Å) and slightly shorter than those found in the zero-valent iron silylene complexes ([(NHSi)Fe(dmpe)_2_] (2.184(2) Å) and Fe(0)[SiNSi] (2.164(15) and 2.170(13) Å)).^15^

Further reduction of this zero-valent Fe species **2** by an excess of KC_8_ at room temperature yielded the three-membered metallacyclic complex **3**, featuring a direct Si–Si bond (Scheme 3). This reaction was also monitored using ^1^H NMR spectroscopy, revealing quantitative conversion (see also Figure S26). The complex was characterized by multinuclear NMR spectroscopy (^1^H, ^13^C, and ^29^Si). The ^1^H NMR signal of the Fe-coordinated η^6^-benzene shifts from δ 5.15 ppm in **2** to δ 5.34 ppm in **3**. The ^29^Si NMR spectrum of **3** contains only one singlet at δ 34.49 ppm, indicating formation of a symmetric molecule.

**Figure 3:**
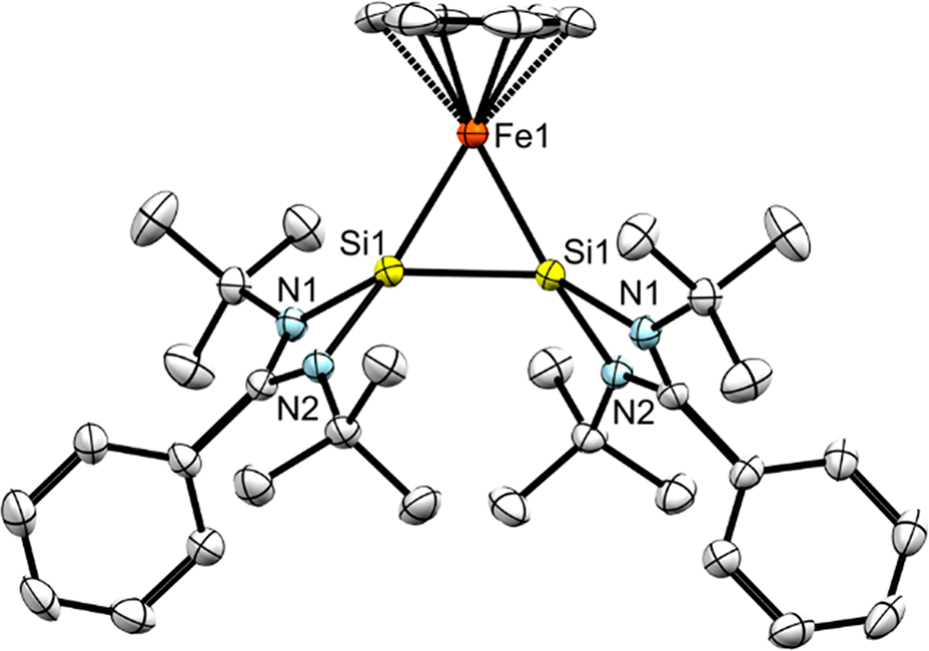
Molecular structure of **3** with thermal ellipsoids drawn at 50% probability. Hydrogen atoms are omitted for clarity. Selected bond lengths (Å) and angles (°): Si1–Si1 and Si1–Fe1, 2.217(6); Si1–N1, 1.865(2); Si1–N2, 1.887(2); Si1–Fe1–Si1, 59.99; Fe1–Si1–Si1, 60.00.

Dark red single crystals of **3** were collected after storing a pentane solution at −30 °C for 1 month. Compound **3** crystallizes in the monoclinic space group *C*_2_/*c*, which is in line with the high degree of symmetry observed in the ^1^H NMR spectrum. The Si(I)–Fe–Si(I) three-membered heterocycle forms an equilateral triangle with interatomic distances of 2.217(6) Å (Figure 3); for the Si–Si bond, this value falls in the range observed for Si=Si double bonds (2.120–2.250 Å).^16^ The isosceles Si–B–Si disilaborirane compound reported by Roesky and co-workers shows a Si–Si bond length of 2.188(5) Å,^17^ while the equilateral triangular (di-*t*-butyl(methyl)silyl)bis(tri-*t*-butylsilyl)cyclotrisilenylium cation has an average Si–Si bond length of 2.217(3) Å.^16^

### Electronic Structure of **3**

Given the unprecedented nature of the ferracyclic motif found in this species, the bonding of the Si(I)–Si(I) fragment to iron(0) was theoretically investigated at the TZ2P/OPBE^18^ level of theory using energy decomposition analysis (EDA).^19^ The lowest energy structure with *C*_2_ symmetry is analogous to that derived from X-ray diffraction. To facilitate the EDA analysis (for symmetry reasons), the bonding of the disilylene moiety to iron has been analyzed using a *C*_2v_ optimized structure, which is only 4.4 kcal·mol^–1^ higher in energy. This small energy difference is consistent with the dynamic behavior observed in solution-state NMR spectroscopy, resulting in a single signal for the ^*t*^Bu substituents at nitrogen.

**Figure 4:**
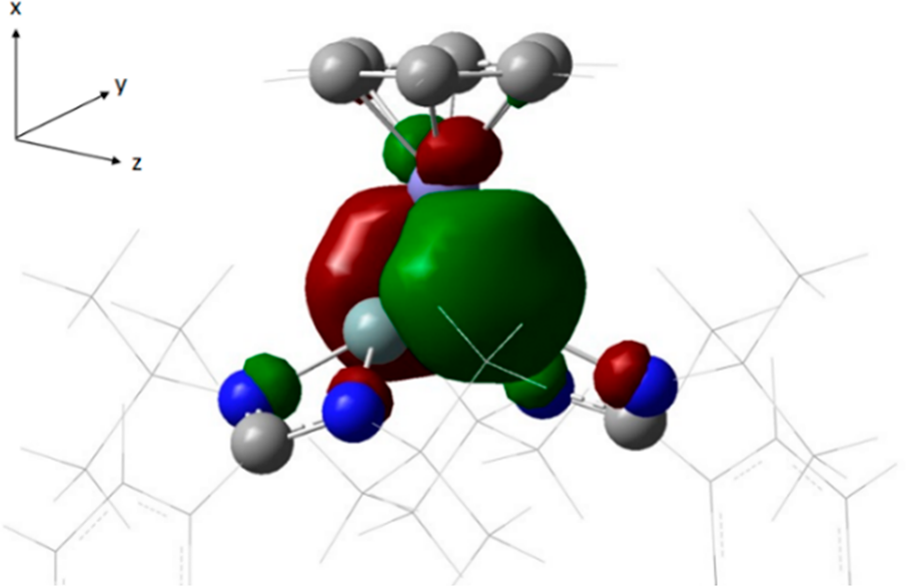
Graphical representation of the Fe-to-(Si–Si) π*-*back donation in **3**.

The Fe(η^6^-benzene) fragment has been computed in the singlet state (Fe(0)-*d*^8^). It has a two-electron occupied d_*xz*_ orbital (perpendicular to the Si–Fe–Si plane) and an empty d_*yz*_ orbital. The electronic structure of the Si(I)–Si(I) fragment is best described as having a Si–Si single bond with a lone pair on each silicon atom and an empty π-orbital perpendicular to that plane (Figure S31). The symmetry-adapted linear combinations of the two lone pairs on silicon donate electron density toward the Fe(η^6^-benzene) fragment, forming two (delocalized) σ-bonds of −30.3 kcal·mol^–1^ (A1) and −74.4 kcal·mol^–1^ (B1). The iron itself donates electron density back from the occupied d_*xz*_ orbital into an empty π-orbital formed by the two Si p_*z*_ orbitals (B2: −67.8 kcal·mol^–1^), giving rise to considerable π-backbonding (Figure 4). To probe the resulting orbital (which is delocalized over the Si–Fe–Si three-membered ring) for aromaticity, we employed the nucleus-independent chemical shift (NICS(0/1)) approach at the B3LYP/6-11+G(d,p) level (negative value of the isotropic magnetic shielding at the center of the Fe–Si–Si three-membered ring and 1 Å perpendicularly above and below, respectively). These are found to be −47.8 and −21.4 ppm, respectively, corroborating the magnetic aromaticity. For comparison, the NICS(0/1) values at the center of the benzene ring in **3** are calculated to be −42.8 and −18.4 ppm. Similar values have been reported for other metallacycles.^20^ A more refined method by Stanger^21^ following the out-of-plane component to the shielding tensor along a trajectory orthogonal to the plane of the ring (NICS_*zz*_) has been used by Roesky *et al.* to assign 2π-aromaticity to their disilaborirane species.^17^ However, instead of showing the typical off-center minimum for 2π-aromatic systems, the NICS scan of **3** shows a steady and continuous increase to less negative NICS_*zz*_ values over a value of 3 Å (see Figure S33), which may be caused by anisotropy of the metal center at the ring and/or by σ-aromaticity.^17,19,22^

Therefore, we resorted to the canonical molecular orbital (CMO) analysis of the NICS(0), which separates the total shieldings into contributions from canonical molecular orbitals.^23^ Indeed, there is a sizable contribution of −13.8 ppm from the Fe d_*xy*_ orbital (HOMO–5, see Figure S32), which is part of the σ-framework. More importantly, the major contribution of −16.6 ppm originates from the delocalized π-orbital shown in Figure 4, substantiating the 2π-aromaticity of the Si_2_Fe three-membered ring in complex **3**.

### Reactivity of **3**

**Figure 5:**
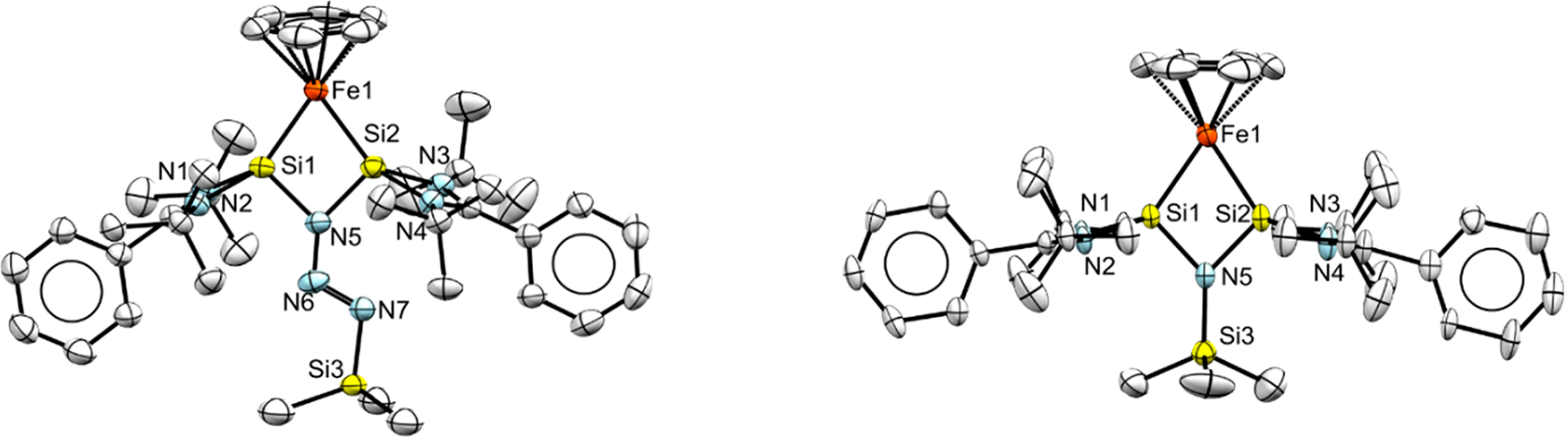
X-ray crystal structures of **4** (left) and **5** (right) with thermal ellipsoids drawn at 50% probability. Hydrogen atoms are omitted for clarity. Selected bond lengths (Å) and angles (°) of **4**: Si1···Si2, 2.5040 (8); Si1–Fe1, 2.1687(6); Si2–Fe1, 2.1746(7); Si1–N5, 1.784(2); Si2–N5, 1.791(2); Si1–Fe1–Si1, 70.41(2); Fe1–Si1–N5, 100.57(6); Fe1–Si2–N5, 100.11(6); Si1–N5–Si2, 88.91(8); **5**: Si1···Si2, 2.412(2); Si1–Fe1, 2.174(2); Si2–Fe1, 2.177(2); Si1–N5, 1.770(5); Si2–N5, 1.767(4); Si1–Fe1–Si1, 67.33(6); Fe1–Si1–N5, 103.30(1); Fe1–Si2–N5, 103.30(1); Si1–N5–Si2, 86.00(2).

**Scheme 4:**
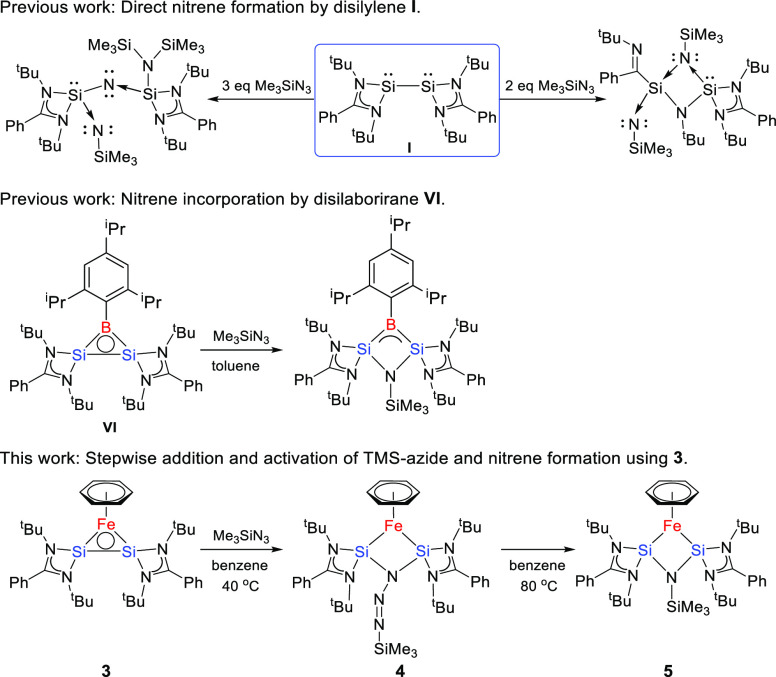
Synthetic Scheme for the Preparation of Azide Adduct **4** and Final Product **5** upon Reaction of **3** with Trimethylsilyl Azide

The unligated Si(I)-based disilylene species **I** shows stoichiometry-dependent reactivity toward trimethylsilyl azide, forming either a silaazatriene or a silaimine product.^24,25^ Roesky and co-workers described ring opening of their disilaborirane species **VI** with TMSN_3_, forming a 1-aza-2,3-disila-4-boretidine derivative (Scheme 4).^17^ We observed selective conversion of **3** with 1 equiv of TMSN_3_ to give the unique azide adduct **4** after 1 h at 40 °C in benzene solution. This species was obtained as a single crystalline material by recrystallization from pentane at −30 °C. The molecular structure, as determined by X-ray diffraction, is displayed in Figure 5. It contains a planar four-membered Si1–Fe1–Si2–N5 heterocycle resulting from the insertion of the azide terminal N-atom in the Si–Si bond, with Fe–Si–N5 angles of roughly 100°. The two Si–Fe distances are slightly different, Si1/2–Fe1 (2.1687(6) and 2.1746(7) Å). The intramolecular Si···Si distance is long at 2.504(8) Å.

Prolonged heating of a mixture of **3** with TMSN_3_ or isolated **4** at 80 °C generated the follow-up trimethylsilylnitrene complex **5** as the major product, together with small amounts of intermediate **4** (see Figure S17). Single crystals of **5**, obtained by recrystallization from benzene at room temperature, were analyzed by X-ray diffraction (Figure 5). Compound **5** also shows a planar four-membered Si1–Fe1–Si2–N5 heterocycle with Fe–Si–N5 angles of 103(1)° but with a different substitution pattern at N5, i.e., only a TMS group resulting from the insertion of trimethylsilylnitrene in the Si–Si bond. The two Si–Fe distances are nearly identical, Si1/2–Fe1 (2.174(2) and 2.177(2) Å), and so are the Si1/2–N5 (1.770(5) and 1.767(4) Å) bond lengths. The Si–Fe σ-bonds are on average 0.2547 Å longer than the Si–B distance in the N-insertion product obtained from disilaborirane,^18^ while the Si–N5 bond length is similar. The intramolecular Si···Si distance (2.412(2) Å) in **5** is shortened relative to that in **4**. Apart from the X-ray crystallographic analysis, reaction monitoring and product identification have also been achieved using NMR spectroscopy. The ^1^H NMR signal of the Fe-bound η^6^-benzene fragment shifts from δ 5.34 ppm (**3**) to δ 5.26 ppm (**4**) and 5.12 ppm (**5**), and the trimethylsilyl group in azide adduct **4** appears at δ 0.47 ppm (for **5**, δ 0.39 ppm) compared to −0.08 ppm for TMSN_3_. In ^29^Si NMR, the N*Si*N signals for both **4** and **5** are strongly upfield-shifted (Δδ ∼ 27 ppm) with respect to **3** (**4**: δ 7.05 ppm; **5**: δ 7.42 ppm), in line with the rupture of the Si–Si bond, which also disrupts the 2π-aromaticity and leads to loss of ring current. The *Si*(CH_3_)_3_ signal appears at δ 15.41 ppm for **4** and at −20.71 ppm for **5**.

**Figure 6:**
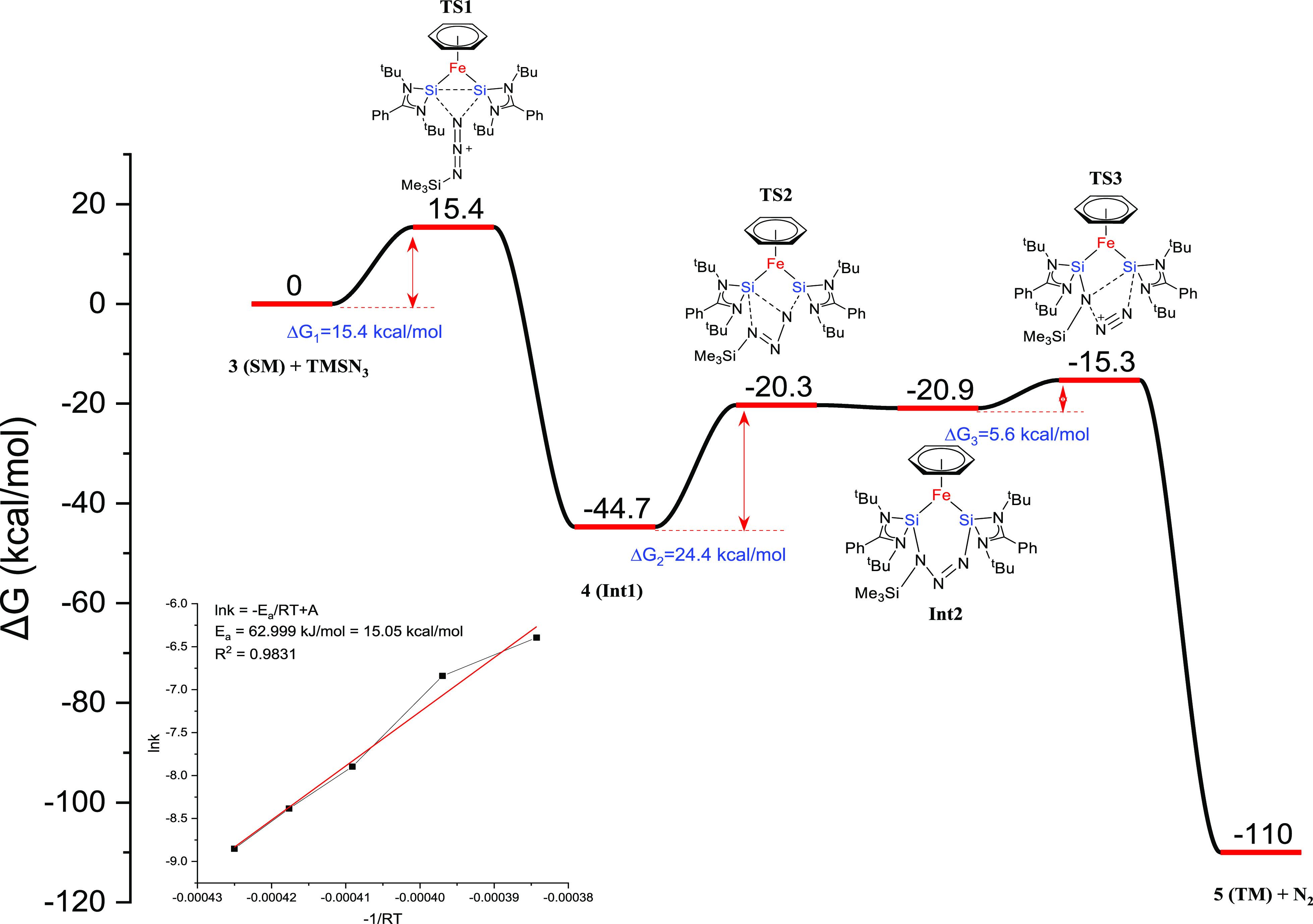
Computed free energy profile for nitrene formation from trimethylsilyl azide and complex **3** (B3LYP-TZVP and def2-TZVP). Transient bonds in transition states are drawn as dashed lines. The inset shows the Arrhenius plot for the consumption of **3** (for details, see the Supporting Information).

To understand the observed two-step reaction between **3** and trimethylsilylazide, we used DFT calculations to explore the reaction mechanism. The initial step involves the nucleophilic attack of the azide onto one of the Si centers, which results in induced nucleophilic character at the second Si center that subsequently attacks back onto the azide in **TS1** (Figure 6). As a result, this insertion can be considered to involve induced FLP reactivity. The energy barrier to afford **Int1** (15.4 kcal·mol^–1^) is consistent with the experimentally determined barrier (15.1 kcal·mol^–1^) for formation of **4** (Arrhenius plot, Figure 5). After forming this symmetric azide adduct, the energy barrier for the subsequent dinitrogen release (29.4 kcal·mol^–1^) is high enough to rationalize the successful isolation of **4**. Finally, a Staudinger pathway, involving four-membered ring transition state **TS3**, releases dinitrogen from **4** to afford product **5** (−110 kcal·mol^–1^).

**Figure 7:**
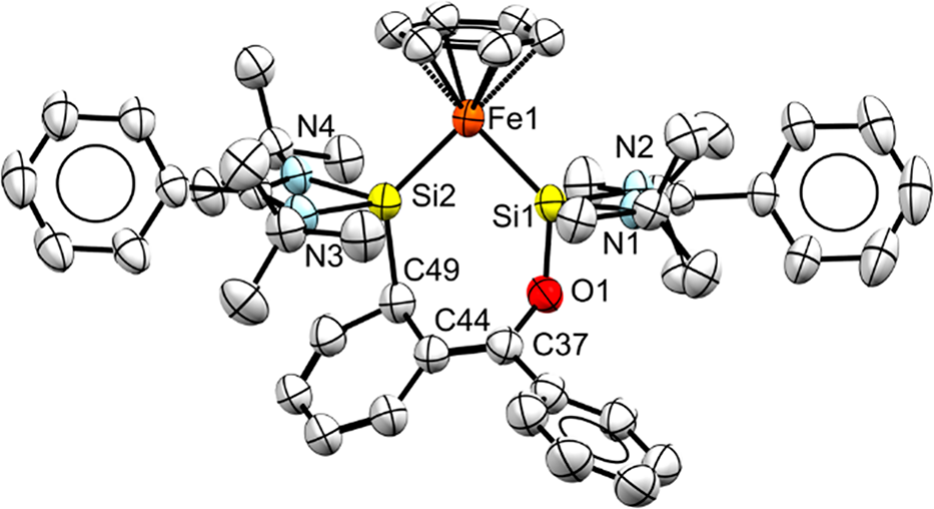
X-ray crystal structure of **6** with thermal ellipsoids drawn at 50% probability. Hydrogen atoms are omitted for clarity. Selected bond lengths (Å) and angles (°) of **6**: Si1···Si2, 3.101(1); Si1–Fe1, 2.146(9); Si2–Fe1, 2.179(9); Si1–O1, 1.688(2); O1–C37, 1.384(3); C37–C44, 1.348(5); C44–C49, 1.503(5); C49–Si2, 1.983(4); Si1–Fe1–Si1, 88.21(4).

**Scheme 5:**
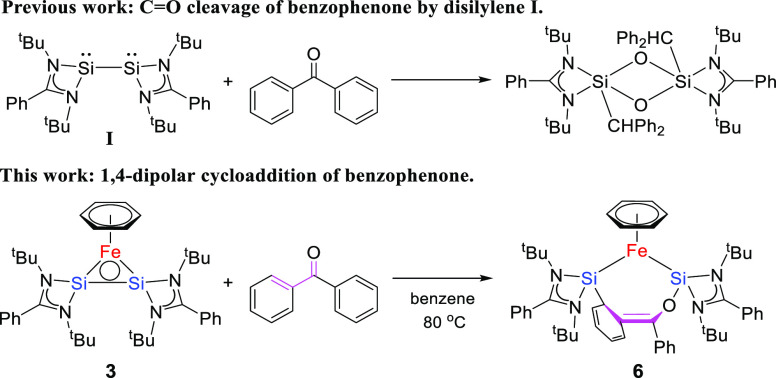
Synthetic Scheme for the Preparation of **6**

The ambivalent reactivity of the Si(I) centers in the Si–Si bond of **3**, able to act either as nucleophile or electrophile, was also apparent during the conversion of species **3** conversion with benzophenone. Disilylene **I** was previously reported to react with benzophenone to furnish selective C–O cleavage with formation of a cyclodisiloxane (Scheme 5).^26^ Strikingly different reactivity was observed when complex **3**, featuring a “protected” Si–Si bond, was exposed to benzophenone for 1 h at 80 °C. Selective formation of seven-membered ring product **6** was obtained via a formal 1,4-addition of benzophenone, displaying strongly attenuated and controlled reactivity of the Si–Si fragment in complex **3**. Complex **6** was characterized in the solid state using single-crystal X-ray diffraction (Figure 7). The structure consists of a nonplanar seven-membered O–Si–Fe–Si–C–C–C ring (angle between the planes O1–C37–C44–C49 and O1–Si1–Fe1–Si2 is 7.20°). The C37–C44 bond length (1.348(5) Å) lies in between that of a typical single carbon bond and a double carbon bond (1.510–1.317 Å).^27^ The Si–Fe–Si angle (∠88.21(4)°) is larger than in species **4** and **5** because of increased steric hindrance. One of the phenyl rings has undergone *ortho*-silylation, concomitant with ring de-aromatization and formation of an enolate-type fragment bound via the oxygen to the second Si center. As a result, overall 1,4-addition of benzophenone has occurred, with no sign of the 1,2-C,O addition product. The ^1^H NMR signal for the η^6^-benzene fragment shifts from δ 5.34 ppm (**3**) to δ 5.07 ppm (**6**). The C–H hydrogen at the *ortho*-silylated position resonates at around δ 6.99 ppm according to 2D-COSY NMR spectroscopy, while the other four hydrogens of the de-aromatized ring appear in the range of 4.90–6.20 ppm. The ^29^Si NMR spectrum of **6** exhibits two sharp singlets at δ 65.57 and δ 33.66 ppm, in accordance with the two different bonding features.

## Conclusions

In summary, we have developed an efficient method to synthesize the first example of a mononuclear transition metal complex bearing a ligated Si(I)–Si(I) disilylene fragment, ferracyclic η^2^-disilylene complex **3**. The electronic structure of this species shows that the Si–Si fragment acts as a four-electron σ-donor to iron, while significant π-back donation from the iron(0) center to the silicon atoms of the disilylene moiety leads to further stabilization of the overall structure. Complex **3** shows well-defined nucleophile-induced FLP reactivity toward TMS-azide and benzophenone, leading to Si–Si bond cleavage by addition of the reagents to the Si_2_ fragment, generating unexpected four- and seven-membered ring structures. Expanding this unique reactivity to other small molecules is currently being explored within our groups.

## Experimental Section

### Materials and Methods

Unless otherwise stated, all manipulations were performed under a nitrogen atmosphere using Schlenk techniques or in a Vigor glovebox maintained at or below 1 ppm of O_2_ and H_2_O. All new metal complexes were prepared and handled in the glovebox under a N_2_ atmosphere. Anhydrous FeCl_2_ (98%) was purchased from Strem Chemicals. PhC(N^*t*^Bu)_2_SiHCl_2_,^28^ LiN(SiMe_3_)_2_(Et_2_O),^28^ Fe(N(SiMe_3_)_2_)_2_,^29^ and complex **1** (FeCl_2_{PhC(N^*t*^Bu)_2_SiCl}_2_)^10^ were synthesized according to reported procedures. Other reagents were purchased from J&K Chemical and SCRC. Glassware was dried at 150 °C overnight. Celite and molecular sieves were dried at 200 °C under vacuum. Benzene, pentane, hexanes, and diethyl ether were degassed with nitrogen, dried over activated molecular sieves, and kept over 4 Å molecular sieves in a N_2_-filled glovebox. NMR data were recorded either on a Bruker 400 or a 500 MHz spectrometer and were internally referenced to residual proton solvent signals in C_6_D_6_ (7.16 ppm). Data for ^1^H NMR are reported as follows: chemical shift (δ ppm) and multiplicity (s = singlet, d = doublet, t = triplet, m = multiplet, br = broad). IR data were recorded on a Thermo Scientific Nicolet iS5 FTIR, and signal strength is represented as follows: VS = very strong, W = weak, S = strong, VW = very weak, m = middle, w = wide. The UV–Vis spectra were recorded using a StellarNet BLACK Comet C-SR diode array miniature spectrophotometer connected to deuterium and halogen lamp by optical fiber using 1 cm matched quartz cuvettes at room temperature. Elemental analysis was performed by the Analytical Laboratory of Shanghai Institute of Organic Chemistry (CAS).

### X-ray Crystallography

Crystals were coated with Paratone-N oil and mounted on a Bruker D8 Venture diffractometer equipped with an APEX-II CCD diffractometer. The crystal was kept at 150 K during data collection. Using Olex2,^30^ the structure was solved with the ShelXT^31^ structure solution program using Intrinsic Phasing and refined with the XL^32^ refinement package using least squares minimization. CCDC 2157512–2157516 contain the supplementary crystallographic data for this paper. These data can be obtained free of charge from The Cambridge Crystallographic Data Centre via www.ccdc.cam.ac.uk/data_request/cif.

### Mössbauer Spectroscopy

Mössbauer spectra were recorded with a ^57^Co source in a Rh matrix using an alternating constant acceleration Wissel Mössbauer spectrometer operated in transmission mode and equipped with a Janis closed-cycle helium cryostat. Isomer shifts are given relative to the iron metal at ambient temperature. Simulation of the experimental data was performed with the Mfit program (developed by Dr. E. Bill, Max-Planck Institute for Chemical Energy Conversion, Mülheim/Ruhr, Germany) using Lorentzian line doublets.

### Computational Details

Geometries were fully optimized as minima or transition states using the Turbomole program package,^33^ coupled to the PQS Baker optimizer^34^ via the BOpt package.^35^ We used unrestricted ri-DFT-D3 calculations at the B3LYP level,^36^ in combination with the def2-TZVP basis set^37^ and a small (m4) grid size. Grimme’s dispersion corrections^38^ (version 3, disp3, “zero damping”) were used to include van der Waals interactions. The energy decomposition analysis (EDA)^19^ was performed on the TZ2P/OPBE^18^ optimized geometry constrained to *C*_2v_ symmetry (+3.9 kcal·mol^–1^). The nucleus-independent chemical shift (NICS)^39^ was used as a diagnostic probe for quantitative measure for aromaticity at the B3LYP/6-11+G(d,p) level.^40,41^ For further details, see the Supporting Information.

#### {PhC(N^*t*^Bu)_2_SiCl}_2_Fe(C_6_H_6_) (**2**)

A solution of **1** (110 mg, 0.153 mmol) in benzene (5 mL) was added dropwise to a solution of KC_8_ (53.7 mg, 0.398 mmol) in THF (5 mL) in a vial while stirring. The color of the reaction mixture turned from yellow to dark red-brown. After stirring for 12 h, volatile materials were removed under vacuum and compound **2** was extracted with pentane solution. The solid was crystallized in pentane solution in a −30 °C freezer for 2 days, and only crystalline material was used for subsequent reactions (yield: 40 mg, 40%). ^1^H NMR (500 MHz, benzene-*d*_6_, ppm) δ 8.04 (m, 1H, Ar-H), 7.26 (d, 1H, Ar-H), 7.09 (d, 1H, Ar-H), 7.00–6.9 (m, 6H, Ar-H), 6.86 (t, 1H, Ar-H), 5.15 (s, 6H, benzene-H), 1.52 (s, 18H, N^*t*^Bu-H), 1.31 (s, 18H, N^*t*^Bu-H). ^13^C NMR (126 MHz, benzene-*d*_6_, ppm) δ 171.84 (N*C*N), 170.75 (N*C*N), 132.55, 132.44, 129.80, 129.70, 129.47, 129.24, 129.02, 128.84, 128.55, 127.63, 127.36 (132.55–127.36: Ph), 80.23 (Fe-benzene), 54.24 (*C*Me_3_), 53.64 (*C*Me_3_), 31.86 (*C*H_3_), 31.39 (*C*H_3_). ^29^Si NMR (99 MHz, benzene-*d*_6_, ppm) δ 45.12, 42.45. UV–Vis (THF, λ (nm) (ε, M^–1^·cm^–1^)): 410 (1604). IR-ATR (cm^–1^): 3059 (VW), 2970 (w), 2928 (VW), 2868 (VW), 1640 (VW), 1577 (VW), 1519 (VW), 1472 (m), 1443 (m), 1415.63 (S), 1389 (S), 1361 (S), 1272 (m), 1203 (S), 1085 (m), 1022 (m), 972 (VW), 926 (W), 882 (W), 789 (W), 753 (S), 726 (W), 708 (S), 636 (m), 617 (S). Anal. calcd for C_36_H_52_Cl_2_FeN_4_Si_2_: C, 59.74; H, 7.24; N, 7.74. Found: C, 57.43; H, 7.29; N, 7.77. Note: Due to the formation of silicon carbide, the carbon values in the elemental analyses were consistently too low for all the disilylene Fe compounds reported in this paper.

#### {PhC(N^*t*^Bu)_2_Si}_2_Fe(C_6_H_6_) (**3**)

A solution of **2** (37 mg, 0.051 mmol) in benzene (10 mL) was added dropwise to KC_8_ (42 mg, 0.311 mmol) in a vial with stirring. About 20 mg of KC_8_ was added every 3 h until all material was converted to {PhC(N^*t*^Bu)_2_Si}_2_Fe(C_6_H_6_), which can be determined by ^1^H NMR monitoring. The color of the reaction mixture turned from red-brown to black. Compound **3** (yield: 29 mg, 89.3%) was collected by removing solvents and volatile materials under vacuum. The solid was stored in pentane solution in a −30 °C freezer for 1 month to give X-ray quality crystals. ^1^H NMR (500 MHz, benzene-*d*_6_) δ 7.14 (m, 2H, Ar-H), 7.00–6.9 (m, 6H, Ar-H), 6.80 (td, 2H, Ar-H), 5.34 (s, 6H, benzene-H), 1.47 (s, 36H, N^*t*^Bu-H). ^13^C NMR (126 MHz, benzene-*d*_6_, ppm) δ 162.70 (N*C*N), 135.72, 129.91, 128.98, 128.93, 127.77, 127.56 (135.72–127.56: Ph), 76.74 (Fe-benzene), 54.62 (*C*Me_3_), 32.78 (*C*H_3_). ^29^Si NMR (99 MHz, benzene-*d*_6_, ppm) δ 34.49. UV–Vis (THF, λ (nm) (ε, M^–1^·cm^–1^)): 390 (4280). IR-ATR (cm^–1^): 3047 (W), 2961 (m), 2922 (m), 2855 (W), 1957 (VW), 1598 (W), 1442 (VW), 1403 (W), 1387 (VS), 1356 (S), 1266 (m), 1203 (S), 1071 (m), 1029 (W), 965 (W), 925 (W), 889 (VW), 836 (VW), 791 (W), 752 (S), 704 (VS), 654 (VW), 610 (m), 560 (VW). Anal. calcd for C_36_H_52_FeN_4_Si_2_: C, 66.23; H, 8.03; N, 8.58. Found: C, 65.11; H, 8.17; N, 8.57.

#### {PhC(N^*t*^Bu)_2_Si}_2_Fe(C_6_H_6_)(N_3_SiMe_3_) (**4**)

Compound **4**, which is also formed during the formation of species **5** (vide infra), can be obtained as an isolable species upon reaction of **3** with TMSN_3_ (7.5 μL, 0.153 mmol) for 1 h at 40 °C in benzene solution in a J-Young tube (quantitative conversion). After 40 min of reaction, the solution was evaporated to dryness under vacuum and redissolved in pentane. X-ray quality crystals were grown from a pentane solution stored in a −30 °C freezer. ^1^H NMR (500 MHz, benzene-*d*_6_) δ 7.32 (dt, 2H, Ar-H), 7.22 (m, 1H, Ar-H), 7.11 (m, 2H, Ar-H), 7.04 (t, 2H, Ar-H), 7.00 (t, 2H, Ar-H), 6.97 (d, 1H, Ar-H), 5.26 (s, 6H, benzene-H), 1.33 (s, 36H, N^*t*^Bu-H), 0.47 (s, 9H, Si(CH_3_)_3_). ^13^C NMR (126 MHz, benzene-*d*_6_, ppm) δ 159.33–127.76 (5 signals; 2 Ph), 76.66 (Fe-benzene), 53.68 (*C*Me_3_), 32.04 (*C*H_3_), −0.08 (Si(*C*H_3_)_3_), −1.23 (N_3_Si(*C*H_3_)_3_). ^29^Si NMR (99 MHz, benzene-*d*_6_, ppm) δ 15.41, 7.05. UV–Vis (THF, λ (nm) (ε, M^–1^·cm^–1^)): 485 (2343). IR-ATR (cm^–1^): 3048 (VW), 2962 (W), 2033 (W), 1523 (VW), 1472 (W), 1422 (S), 1391 (W), 1358 (W), 1274 (W), 1237 (W), 1207 (S), 1148 (m), 1078 (W), 1021 (W), 989 (m), 966 (W), 923 (W), 832 (S), 789 (W), 751 (S), 724 (W), 704 (S), 641 (W), 609 (W). Elemental analysis of this species did not yield satisfactory results, which is attributed to the demonstrated thermal instability of this complex, leading to “decomposition” to form species **5**.

#### {PhC(N^*t*^Bu)_2_Si}_2_Fe(C_6_H_6_)(NSiMe_3_) (**5**)

TMSN_3_ (7.5 μL, 0.153 mmol) was added by a pipette to a solution of **3** (36 mg, 0.055 mmol) in a J-Young tube or Schlenk tube while stirring at 80 °C. After heating the reaction mixture for 90 min, the product was isolated (yield: 20.3 mg, 50%) as a purple-red solid by removing all solvents and other volatile materials under vacuum and washing with cold pentane. At shorter reaction times, this product coexists with complex **4** according to NMR spectroscopy. X-ray quality crystals were collected by dissolving the obtained solid in benzene solution and slow evaporation of the solution at room temperature. ^1^H NMR (500 MHz, benzene-*d*_6_) δ 7.36 (s, 1H, Ar-H), 7.31 (d, 3H, Ar-H), 7.01–6.99 (m, 4H, Ar-H), 6.96 (m, 2H, Ar-H), 5.12 (s, 6H, benzene-H), 1.43 (s, 36H, N^*t*^Bu-H), 0.39 (s, 9H, Si(CH_3_)_3_). ^13^C NMR (126 MHz, benzene-*d*_6_, ppm) δ 169.61 (N*C*N), 133.69, 129.94, 128.47, 127.67 (133.69–127.67: Ph), 75.31 (Fe-benzene), 53.61 (*C*Me_3_), 31.75 (*C*H_3_), 3.79 (NSi(*C*H_3_)_3_). ^29^Si NMR (99 MHz, benzene-*d*_6_, ppm) δ 7.42, −20.71. UV–Vis (THF, λ (nm) (ε, M^–1^·cm^–1^)): 530 (592). IR-ATR (cm^–1^): 3357 (VW), 2961 (w), 2029 (S), 1597 (W), 1473 (VW), 1419 (S), 1392 (W), 1358 (m), 1237 (W), 1204 (S), 1074 (W), 1019 (S), 924 (VW), 832 (S), 787 (VW), 746 (S), 704 (S), 649 (VW), 641 (VW), 609 (W). Anal. calcd for C_39_H_61_FeN_5_Si_3_: C, 63.30; H, 8.31; N, 9.46. Found: C, 60.19; H, 7.99; N, 8.82.

#### {PhC(NtBu)_2_Si}_2_Fe(C_6_H_6_)(Ph_2_CO) (**6**)

Benzophenone (437 μL of a 100 mg/10 mL stock solution in benzene) was added dropwise to a solution of **3** (15.6 mg) in benzene (5 mL) in a J-Young tube or Schlenk tube at 80 °C with stirring. Product **6** was isolated as a dark greenish-black solid by washing with cold pentane after removing all solvents and other volatile materials. Yield: 12 mg, 60%. X-ray quality crystals were collected by dissolving the solid in diethyl ether solution and then placing the sample in a −30 °C freezer. ^1^H NMR (500 MHz, benzene-*d*_6_) δ 8.36 (d, 0.5H, Ar-H), 7.76 (d, 2H, Ar-H), 7.52 (d, 1H, Ar-H), 7.31 (d, 0.5H, Ar-H), 7.25 (t, 2H, Ar-H), 7.06–6.95 (m, 9H, Ar-H), 6.98 (d, 1H, de-ArCH), 6.17 (dd, 1H, de-ArCH), 5.86 (m, 1H, de-ArCH), 5.65 (dd, 1H, de-ArCH), 4.90 (d, 1H, de-ArCH), 5.07 (s, 6H, benzene-H), 1.59/1.45/1.31/0.97 (s, 9H, N^*t*^Bu-H). ^13^C NMR (126 MHz, benzene-*d*_6_, ppm) δ 170.46 (N*C*N), 167.56 (N*C*N), 159.33–122.22 (18 signals; 6 C + 18 C; CCCHCHCHCH + C of 3 Ph), 78.46 (Fe-benzene), 53.23 (*C*Me_3_), 53.14 (*C*Me_3_), 52.67 (*C*Me_3_), 52.38 (*C*Me_3_), 32.12 (*C*H_3_), 32.01 (*C*H_3_), 31.15 (*C*H_3_), 31.09 (*C*H_3_). ^29^Si NMR (99 MHz, benzene-*d*_6_, ppm) δ 65.57, 33.66. UV–Vis (THF, λ(nm) (ε, M^–1^·cm^–1^)): 350 (4785), 435 (2782). IR-ATR (cm^–1^): 3052 (VW), 2965 (W), 2962 (VW). 1647 (VW), 1596 (VW), 1472 (W), 1421 (S), 1390 (m), 1358 (m), 1264 (m), 1204 (S), 1106 (VW), 1072 (W), 1009 (W), 970 (W), 923 (W), 865 (W), 790 (m), 745 (S), 722 (VW), 699 (VS), 607 (S), 544 (S). Anal. calcd for C_49_H_62_FeN_4_OSi_2_: C, 70.48; H, 7.48; N, 6.71. Found: C, 65.07; H, 7.09; N, 6.15.

## Supporting Information Available

General information; synthetic procedures of new compounds, including NMR, IR, and UV–Vis spectra; reaction of **3** with TMS-azide followed by NMR; EDA and DFT calculations; single-crystal X-ray data diffraction (PDF)
Computational details (ZIP)
