# Supplementary material for: Reactivity of a Unique Si(I)–Si(I)-Based η2-Bis(silylene) Iron Complex
Source: Inorg Chem. 2022 Jul 20;61(30):11725–33. doi: 10.1021/acs.inorgchem.2c01369 (PMC9377512; doi:10.1021/acs.inorgchem.2c01369)
Supplement: Supplementary file 1 — ic2c01369_si_001.pdf [file ic2c01369_si_001.pdf]

**SUPPORTING INFORMATION**

For

# Reactivity of a Unique Si(I)-Si(I)-based $\eta^2$-bis(silylene) Iron Complex


Zhiyuan He,[a, b‡] Lingyu Liu,[a‡] Felix J. de Zwart,[b] Xiaolian Xue,[a] Andreas W. Ehlers,[b,d] Kaking Yan,[a] Serhiy Demeshko,[e] Jarl Ivar van der Vlugt,[b,c*] Bas de Bruin,[b*] and Jeremy Krogman[a*]

[a] School of Physical Science and Technology, ShanghaiTech University, Shanghai 201210, China. [b] van 't Hoff Institute for Molecular Sciences, University of Amsterdam, Science Park 904, 1098 XH Amsterdam, The Netherlands. [c] Institute of Chemistry, Carl von Ossietzky University, Carl-von-Ossietzky-Straße 9-11, 12629 Oldenburg, Germany. [d] Department of Chemistry, University of Johannesburg, Auckland Park, Johannesburg, South Africa. [e] Department of Chemistry, Georg August University, Tammanstraße 4, 37077 Göttingen, Germany.

*Prof. Dr. J. Krogman. E-mail: jkrogman@shanghaitech.edu.cn

*Prof. Dr. B. de Bruin. E-mail: b.debruin@uva.nl

*Prof. Dr. J.I. van der Vlugt. E-mail: jarl.ivar.van.der.vlugt@uni-oldenburg.de


## Table of Contents


General information....................2

Synthetic procedures of new compounds, including NMR, IR and UV-vis spectra....................3

Reaction of 3 with TMS-azide followed by NMR....................23

EDA and DFT calculations....................26

Single crystal X-ray data diffraction....................49

Supplementary References....................54

# General information

Unless otherwise stated, all manipulations were performed under a nitrogen atmosphere using Schlenk techniques or in a Vigor glovebox maintained at or below 1 ppm of $O_2$ and $H_2O$. All new metal complexes were prepared and handled in the glovebox under $N_2$ atmosphere. Anhydrous $FeCl_2$ (98%) was purchased from Strem Chemicals. $PhC(N^tBu)_2SiHCl_2$,[S1] $LiN(SiMe_3)_2(Et_2O)$[S1] and $Fe(N(SiMe_3)_2)_2$[S2] and complex **1**, $FeCl_2\{PhC(N^tBu)_2SiCl\}_2$,[S3] were synthesized according to reported procedures. Other reagents were purchased from J&K Chemical and SCRC. Glassware was dried at 150 °C overnight. Celite and molecular sieves were dried at 200 °C under vacuum. Benzene, pentane, hexanes, and diethyl ether were degassed with nitrogen and dried over activated molecular sieves, and kept over 4 Å molecular sieves in a $N_2$-filled glovebox. NMR data were recorded either on a Bruker 400 or a 500 MHz spectrometer, and are internally referenced to residual proton solvent signals in $C_6D_6$ (7.16 ppm). Data for $^1$H NMR are reported as follows: chemical shift (δ ppm), multiplicity (s = singlet, d = doublet, t = triplet, m = multiplet, br = broad), IR data were recorded on a Thermo Scientific Nicolet iS5 FTIR and signal strength is represented as follows: VS=very strong, W=weak, S=strong, VW=very weak, m=middle, w=wide. UV-vis spectra were recorded using a StellarNet BLACK Comet C-SR diode array miniature spectrophotometer connected to deuterium and halogen lamp by optical fiber using 1 cm matched quartz cuvettes at room temperature. Elemental analysis was performed by the Analytical Laboratory of Shanghai Institute of Organic Chemistry (CAS).

**X-ray crystallography**:

Crystals were coated with Paratone-N oil and mounted on a Bruker D8 Venture diffractometer equipped with an APEX-II CCD diffractometer. The crystal was kept at 150 K during data collection. Using Olex2,[S4] the structure was solved with the ShelXT[S5] structure solution program using Intrinsic Phasing and refined with the XL[S6] refinement package using least squares minimization. CCDC 2157512-2157516 contain the supplementary crystallographic data for this paper. These data can be obtained free of charge from The Cambridge Crystallographic Data Centre via www.ccdc.cam.ac.uk/data_ request/cif.

**Mössbauer spectroscopy**:

Mössbauer spectra were recorded with a $^{57}$Co source in a Rh matrix using an alternating constant acceleration *Wissel* Mössbauer spectrometer operated in the transmission mode and equipped with a *Janis* closed-cycle helium cryostat. Isomer shifts are given relative to iron metal at ambient temperature.

Simulation of the experimental data was performed with the *Mfit* program (developed by Dr. E. Bill, Max-Planck Institute for Chemical Energy Conversion, Mülheim/Ruhr, Germany) using *Lorentzian* line doublets.

# Synthetic procedures of new compounds, including NMR, IR and UV-vis spectra

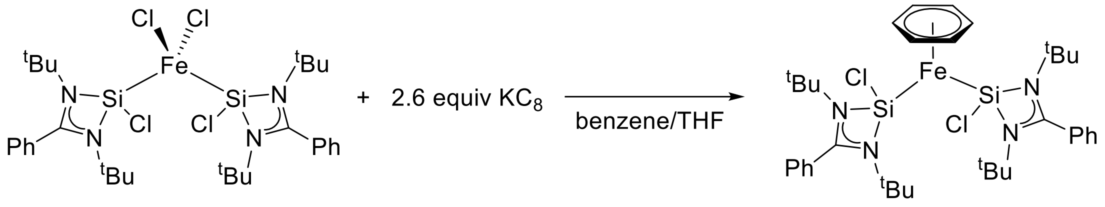


**1** **2**

**{PhC(N$^t$Bu)$_2$SiCl}$_2$Fe(C$_6$H$_6$)** (**2**).

A solution of **1** (110 mg, 0.153 mmol) in benzene (5 mL) was added dropwise to a solution of $KC_8$ (53.7 mg, 0.398 mmol) in THF (5 mL) in a vial whilst stirring. The color of the reaction mixture turned from yellow to dark red-brown. After stirring for 12 hours, volatile materials were removed under vacuum and compound **2** was extracted with pentane solution. The solid was crystallized in pentane solution in a −30 °C freezer for 2 days, and only crystalline material was used for subsequent reactions (Yield: 40 mg, 40%).

$^1$H NMR (500 MHz, benzene-$d_6$, ppm) δ 8.04 (m, 1H, Ar-H), 7.26 (d, 1H, Ar-H), 7.09 (d,1H, Ar-H), 7.00~6.9 (m, 6H, Ar-H), 6.86 (t, 1H, Ar-H), 5.15 (s, 6H, benzene-H), 1.52 (s, 18H, N$^t$Bu-H), 1.31 (s, 18H, N$^t$Bu-H).

$^{13}$C NMR (126 MHz, benzene-$d_6$, ppm) δ 171.84 (N*C*N), 170.75 (N*C*N), 132.55, 132.44, 129.80, 129.70, 129.47, 129.24, 129.02, 128.84, 128.55, 127.63, 127.36 (132.55 ~127.36: Ph), 80.23 (Fe-benzene), 54.24 (*C*Me$_3$), 53.64 (*C*Me$_3$), 31.86 (*C*H$_3$), 31.39 (*C*H$_3$).

$^{29}$Si NMR (99 MHz, benzene-$d_6$, ppm) δ 45.12, 42.45.

UV-Vis (THF, λ(nm) (ε, $M^{-1}cm^{-1}$)): 410 (1604).

IR-ATR ($cm^{-1}$): 3059 (VW), 2970 (w), 2928 (VW), 2868 (VW), 1640 (VW), 1577 (VW), 1519 (VW), 1472 (m), 1443 (m), 1415.63 (S), 1389 (S), 1361 (S), 1272 (m), 1203 (S), 1085 (m), 1022 (m), 972 (VW), 926 (W), 882 (W), 789 (W), 753 (S), 726 (W), 708 (S), 636 (m), 617 (S).

Anal. Calcd for $C_{36}H_{52}Cl_2FeN_4Si_2$: C, 59.74; H, 7.24; N, 7.74. Found: C, 57.43; H, 7.29; N, 7.77. Due to the formation of silicon carbide the carbon values in the elemental analyses were consistently too low for the disilylene Fe compounds reported in this paper.

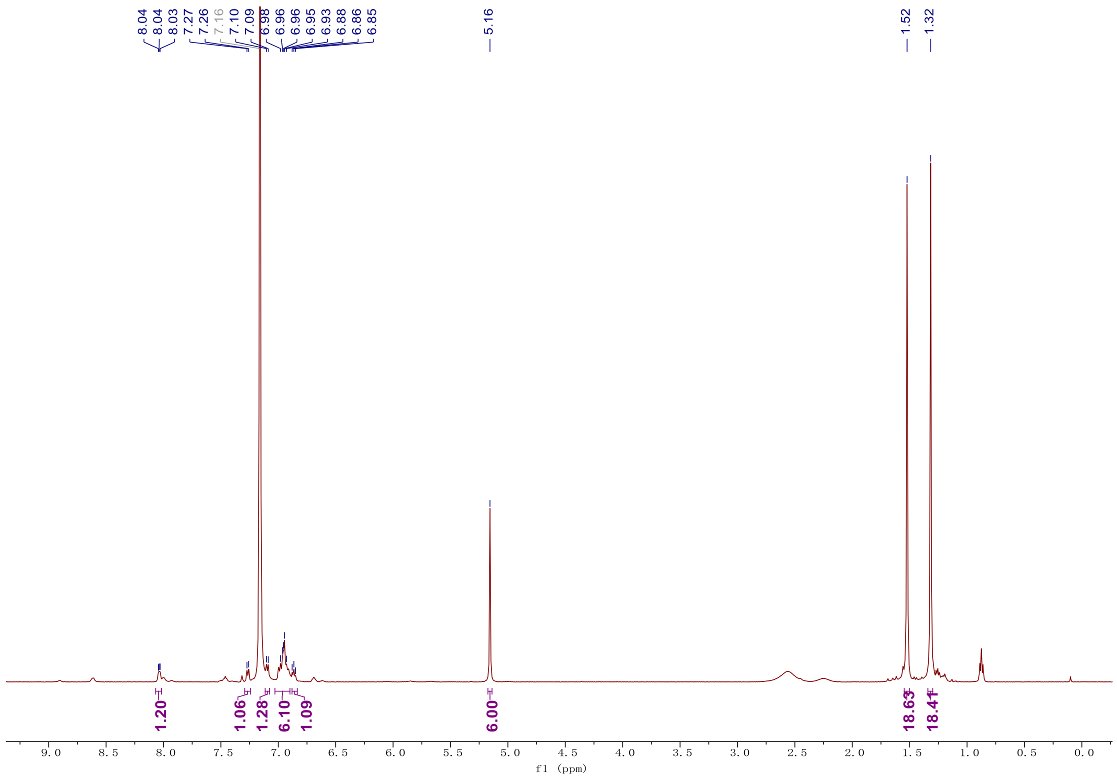

Figure S1. $^{1}$H NMR spectrum of $\{PhC(N^{t}Bu)_2SiCl\}_2Fe(C_6H_6)$ (**2**) (500 MHz, $C_6D_6$).

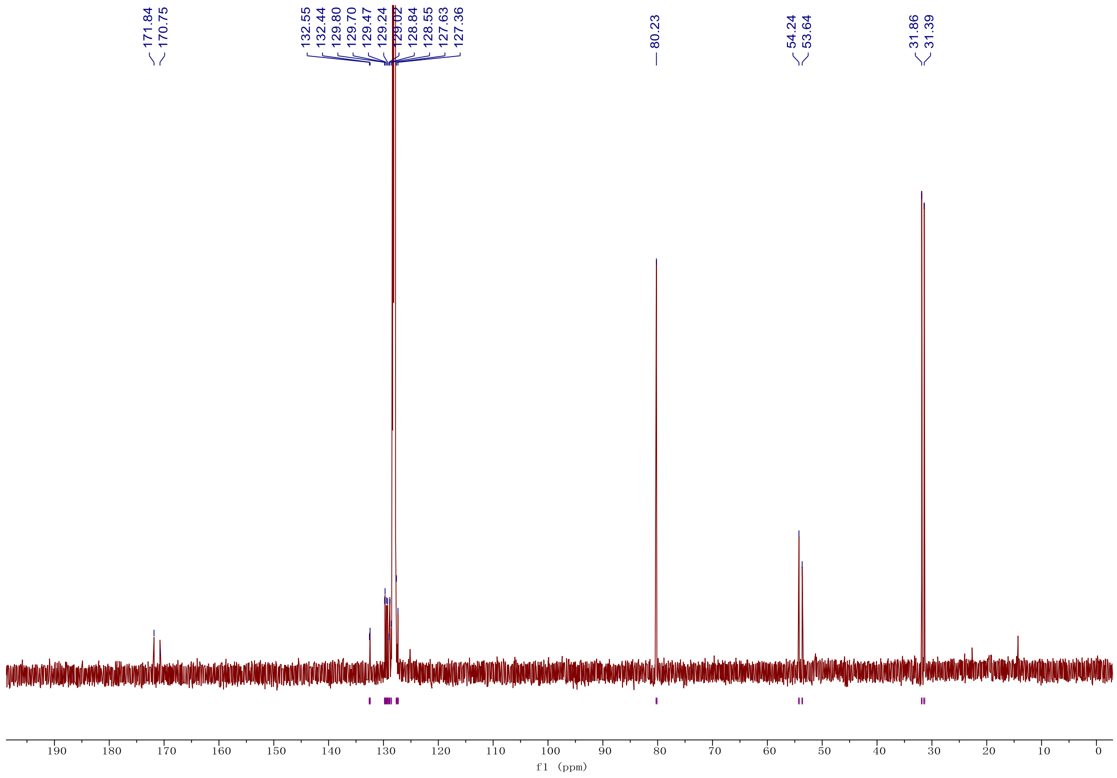

Figure S2. $^{13}$C NMR spectrum of $\{PhC(N^{t}Bu)_2SiCl\}_2Fe(C_6H_6)$ (**2**) (126 MHz, $C_6D_6$).

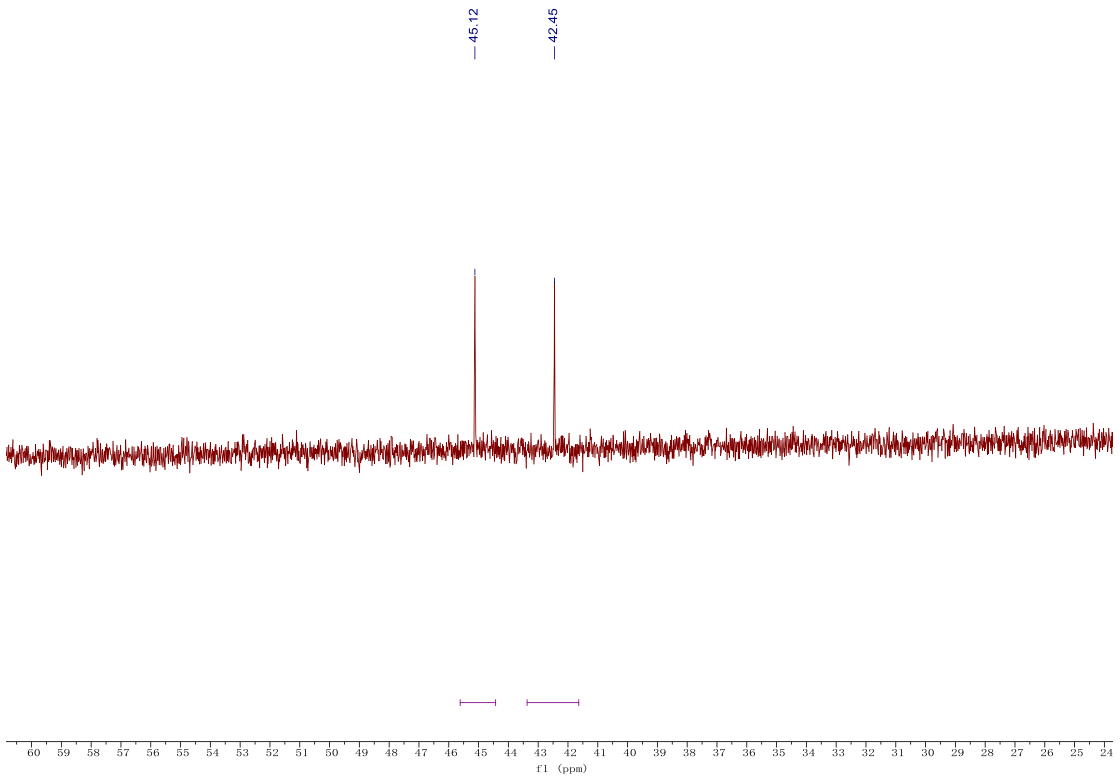


Figure S3. $^{29}$Si NMR spectrum of {PhC(N$^t$Bu)$_2$SiCl}$_2$Fe($C_6H_6$) (**2**) (99 MHz, $C_6D_6$).

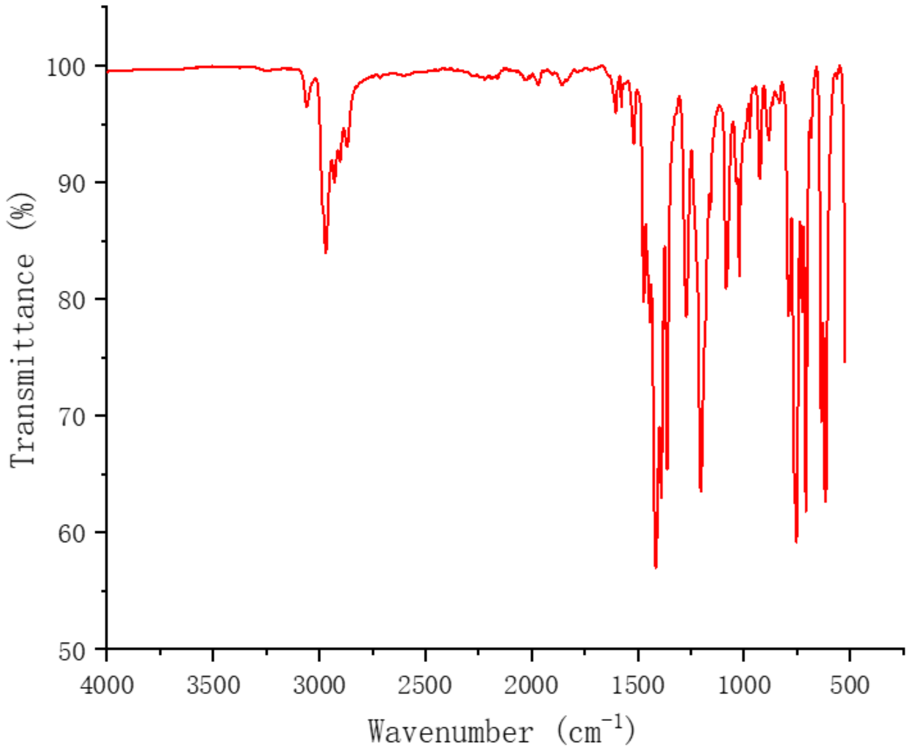


Figure S4. IR spectrum of {PhC(N$^t$Bu)$_2$SiCl}$_2$Fe($C_6H_6$) (**2**).

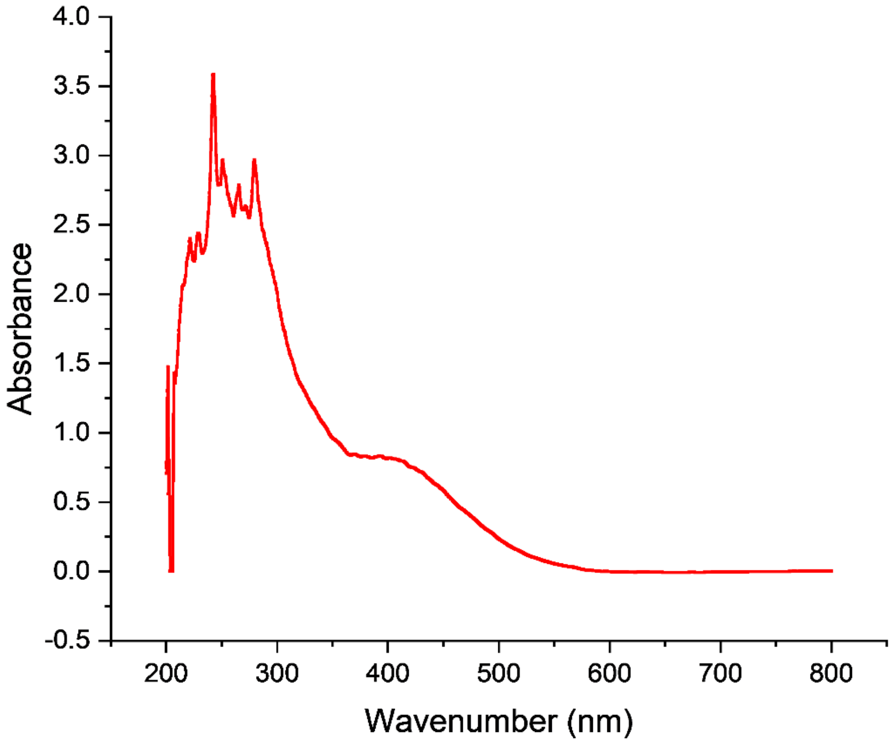

Figure S5. UV-vis spectrum of {PhC(N$^t$Bu)$_2$SiCl}$_2$Fe($C_6H_6$) (**2**).

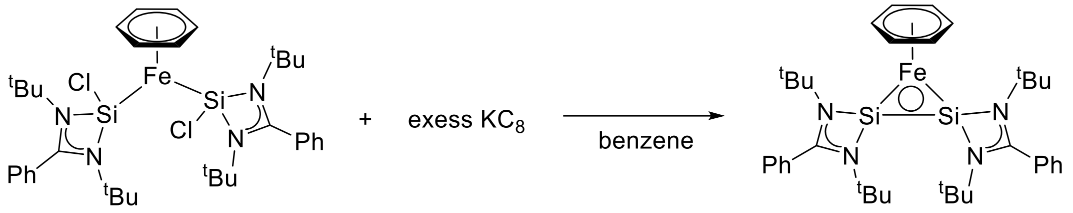


**2** **3**

**{PhC(N$^{t}$Bu)$_2$Si}$_2$Fe(C$_6$H$_6$) (3)**.

A solution of {PhC(N$^{t}$Bu)$_2$SiCl}$_2$Fe(C$_6$H$_6$) (37 mg, 0.051 mmol) in benzene (10 mL) was added dropwise to KC$_8$ (42 mg, 0.311 mmol) in a vial with stirring. About 20 mg KC$_8$ was added every three hours until all material was converted to {PhC(N$^{t}$Bu)$_2$Si}$_2$Fe(C$_6$H$_6$), which can be determined by $^1$H NMR monitoring. The color of the reaction mixture turned from red-brown to black. Compound **3** (Yield: 29 mg, 89.3%) was collected by removing solvents and volatile materials under vacuum. The solid was stored in pentane solution in a –30 °C freezer for 1 month to give X-ray quality crystals.

$^1$H NMR (500 MHz, benzene-*d*$_6$) δ 7.14 (m, 2H, Ar-H), 7.00~6.9 (m, 6H, Ar-H), 6.80 (td, 2H, Ar-H), 5.34 s, 6H, benzene-H), 1.47 (s, 36H, N$^{t}$Bu-H).

$^{13}$C NMR (126 MHz, benzene-*d*$_6$, ppm) δ 162.70 (N*C*N), 135.72, 129.91, 128.98, 128.93, 127.77, 127.56 (135.72~127.56: Ph), 76.74 (Fe-benzene), 54.62 (*C*Me$_3$), 32.78 (*C*H$_3$).

$^{29}$Si NMR (99 MHz, benzene-*d*$_6$, ppm) δ 34.49.

UV-Vis (THF, λ(nm) (ε, M$^{-1}$cm$^{-1}$)): 390 (4280).

IR-ATR (cm$^{-1}$): 3047 (W), 2961 (m), 2922 (m), 2855 (W), 1957 (VW), 1598 (W), 1442 (VW), 1403 (W), 1387 (VS), 1356 (S), 1266 (m), 1203 (S), 1071(m), 1029 (W), 965 (W), 925 (W), 889 (VW), 836 (VW), 791 (W), 752 (S), 704 (VS), 654 (VW), 610 (m), 560 (VW).

Anal. Calcd for $C_{36}H_{52}FeN_4Si_2$: C, 66.23; H, 8.03; N, 8.58. Found: C, 65.11; H, 8.17; N, 8.57. Due to the formation of silicon carbide the carbon values in the elemental analyses were consistently too low for the disilylene Fe compounds reported in this paper.

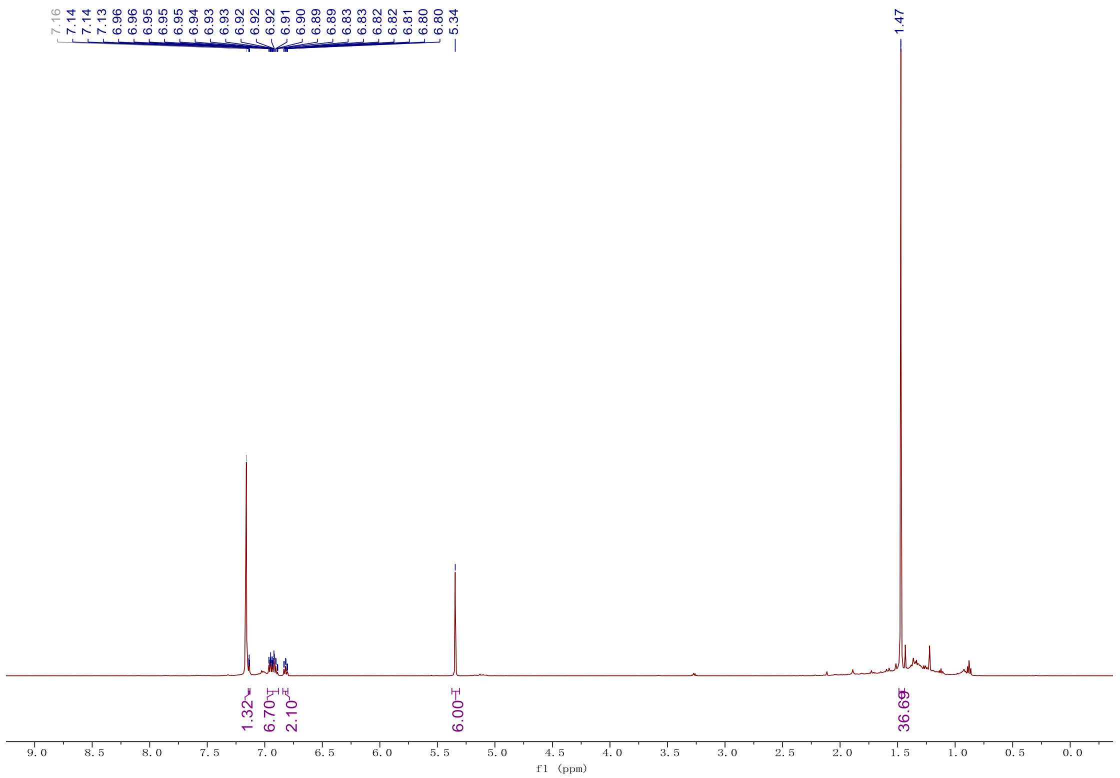


Figure S6. $^{1}H$ NMR spectrum of $\{PhC(N^{t}Bu)_2Si\}_2Fe(C_6H_6)$ (**3**) (500 MHz, $C_6D_6$).

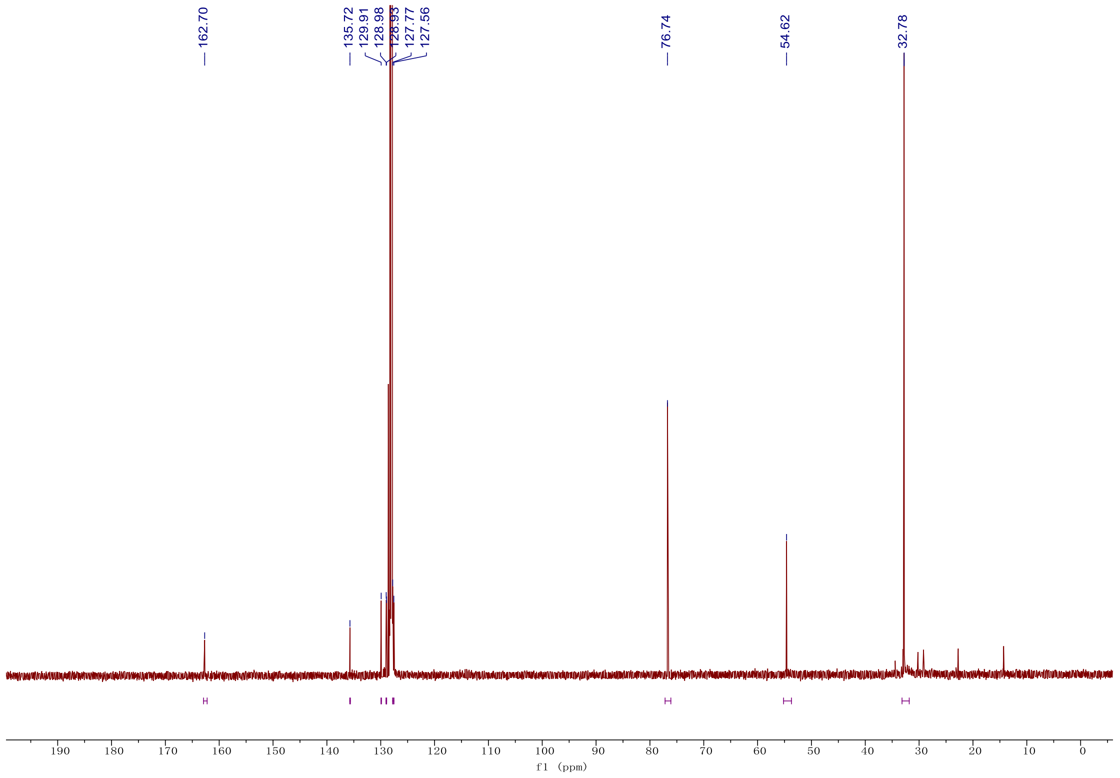


Figure S7. $^{13}C$ NMR spectrum of $\{PhC(N^{t}Bu)_2Si\}_2Fe(C_6H_6)$ (**3**) (126 MHz, $C_6D_6$).

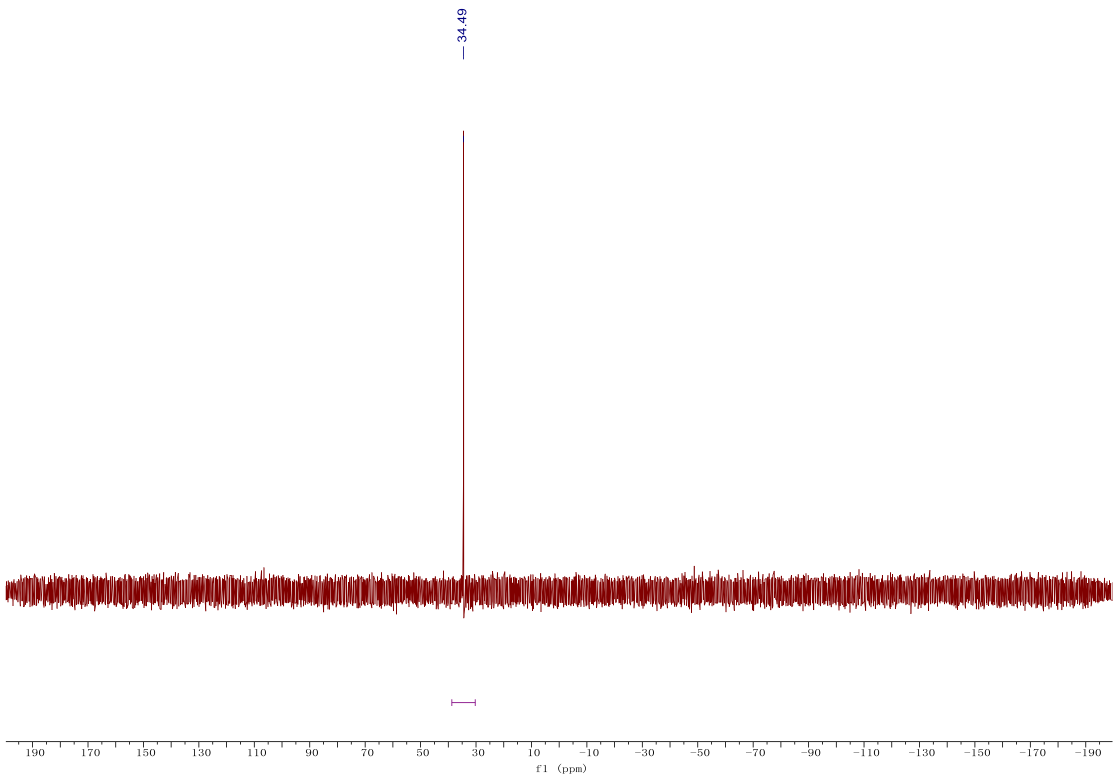


Figure S8. $^{29}$Si NMR spectrum of {PhC(N$^t$Bu)$_2$Si}$_2$Fe($C_6H_6$) (**3**) (99 MHz, $C_6D_6$).

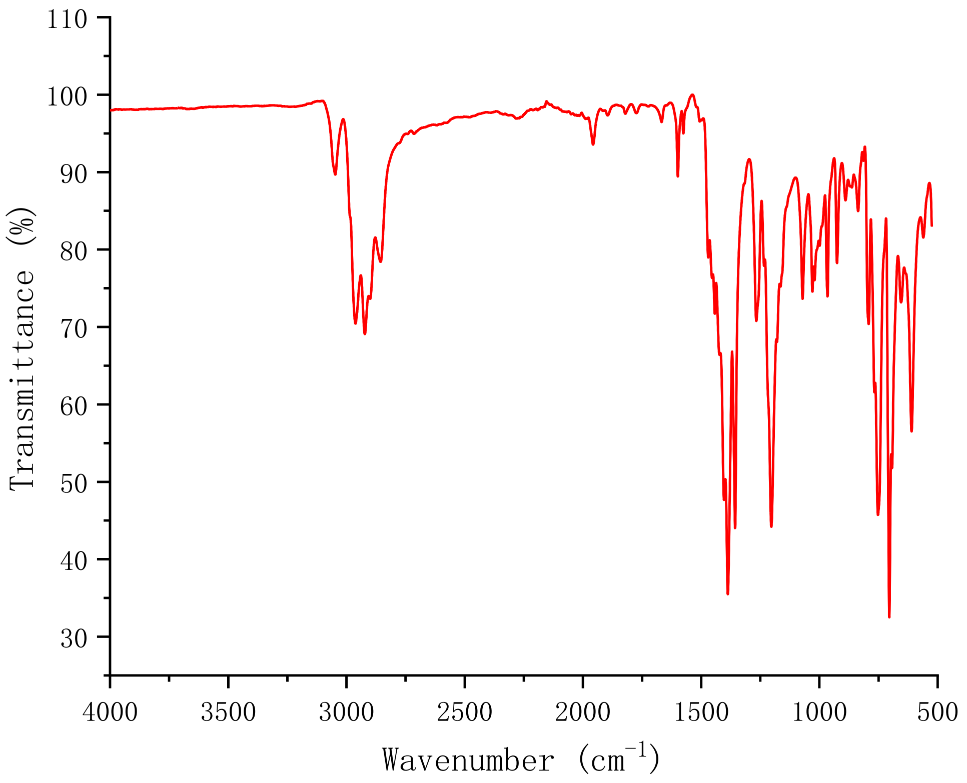


Figure S9. IR spectrum of {PhC(N$^t$Bu)$_2$Si}$_2$Fe($C_6H_6$) (**3**).

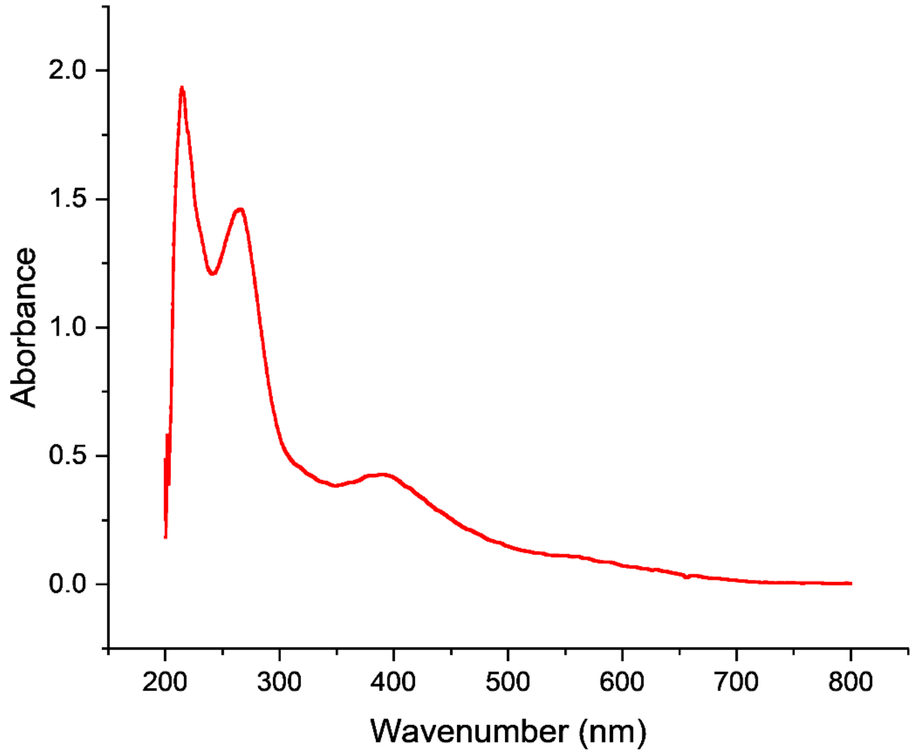


Figure S10. UV-vis spectrum of {PhC(N$^t$Bu)$_2$Si}$_2$Fe($C_6H_6$) (**3**).

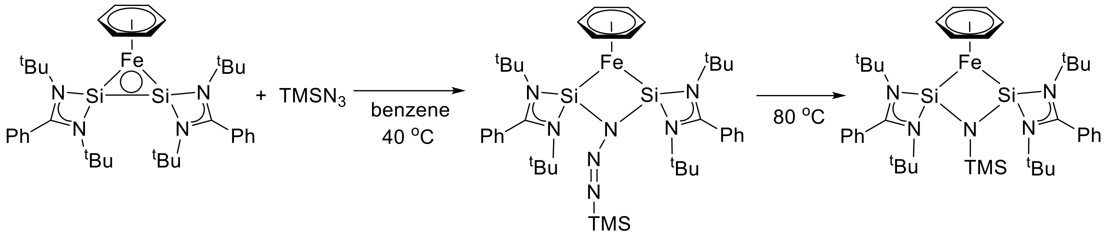

3 4 5

**{PhC(N$^t$Bu)$_2$Si}$_2$Fe(C$_6$H$_6$)(N$_3$SiMe$_3$) (4).**

Compound **4**, which is also formed during the formation of species **5** (*vide infra*), can be obtained as an isolable species upon reaction of **3** with TMSN$_3$ (7.5 µL, 0.153 mmol) for 1 hour at 40 °C in benzene solution in a J-Young tube (quantitative conversion). After 40 minutes of reaction, the solution was evaporated to dryness under vacuum and redissolved in pentane. X-ray quality crystals were grown from a pentane solution stored in a -30 °C freezer.

$^1$H NMR (500 MHz, benzene-*d$_6$*) δ 7.32 (dt, 2H, Ar-H), 7.22 (m, 1H, Ar-H), 7.11 (m, 2H, Ar-H), 7.04 (t, 2H, Ar-H), 7.00 (t, 2H, Ar-H), 6.97 (d, 1H, Ar-H), 5.26 (s, 6H, benzene-H), 1.33 (s, 36H, N$^t$Bu-H), 0.47 (s, 9H, Si(CH$_3$)$_3$).

$^{13}$C NMR (126 MHz, benzene-*d$_6$*, ppm) δ 159.33~127.76 (5 signals; 2 Ph), 76.66 (Fe-benzene), 53.68 (*C*Me$_3$), 32.04 (*C*H$_3$), –0.08 (Si(*C*H$_3$)$_3$), –1.23 (C: TMSN$_3$).

$^{29}$Si NMR (99 MHz, benzene-*d$_6$*, ppm) δ 15.41, 7.05.

UV-Vis (THF, λ(nm) (ε, M$^{-1}$cm$^{-1}$)): 485 (2343).

IR-ATR (cm$^{-1}$): 3048 (VW), 2962 (W), 2033 (W), 1523 (VW), 1472 (W), 1422 (S), 1391 (W), 1358 (W), 1274 (W), 1237 (W), 1207 (S), 1148 (m), 1078 (W), 1021 (W), 989 (m), 966 (W), 923 (W), 832 (S), 789 (W), 751 (S), 724 (W), 704 (S), 641 (W), 609 (W).

Elemental analysis of this species did not yield satisfactory results, which are attributed to the demonstrated thermal instability of this complex with 'decomposition' to species **5**.

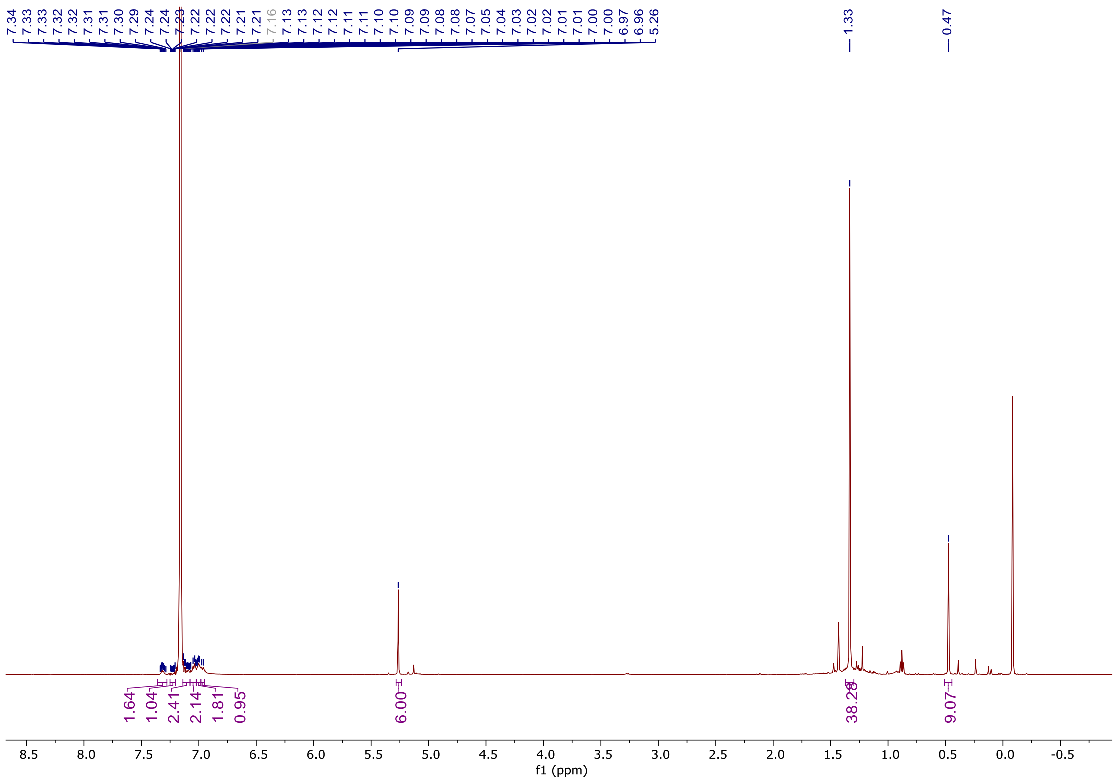


Figure S11. $^{1}$H NMR spectrum of {PhC(N$^t$Bu)$_2$Si}$_2$Fe($C_6H_6$)($N_3SiMe_3$) (**4**) (500 MHz, $C_6D_6$).

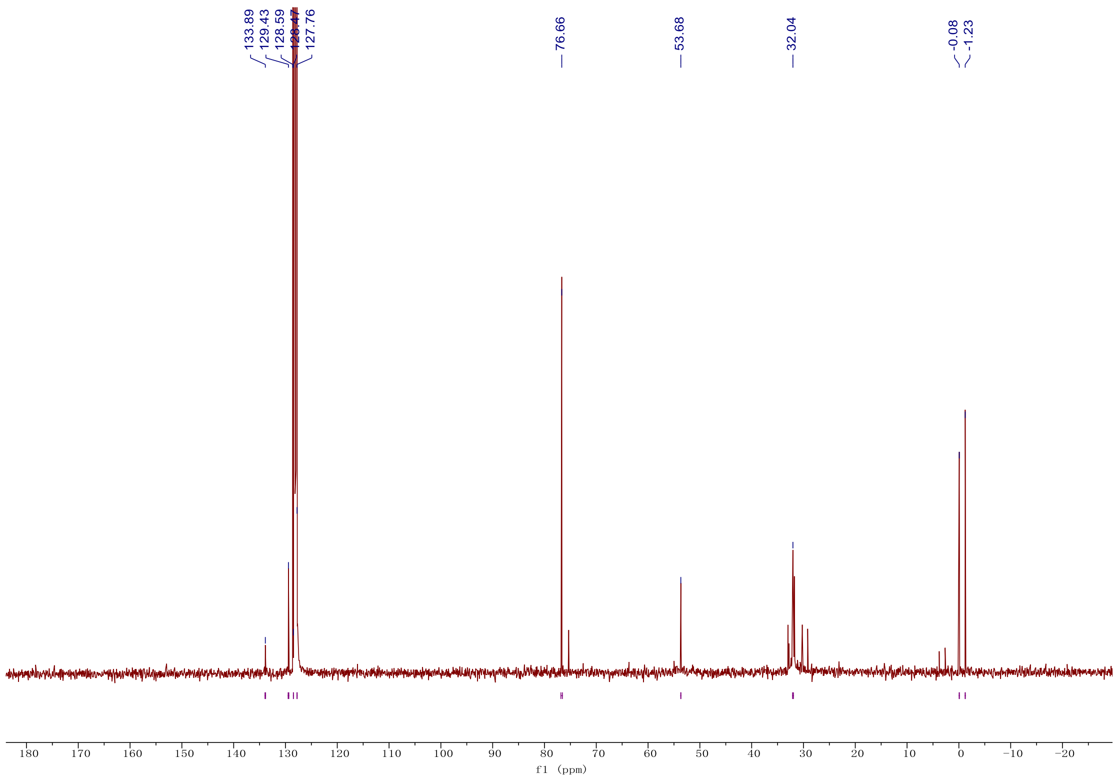


Figure S12. $^{13}$C NMR spectrum of {PhC(N$^t$Bu)$_2$Si}$_2$Fe($C_6H_6$)($N_3SiMe_3$) (**4**) (126 MHz, $C_6D_6$).

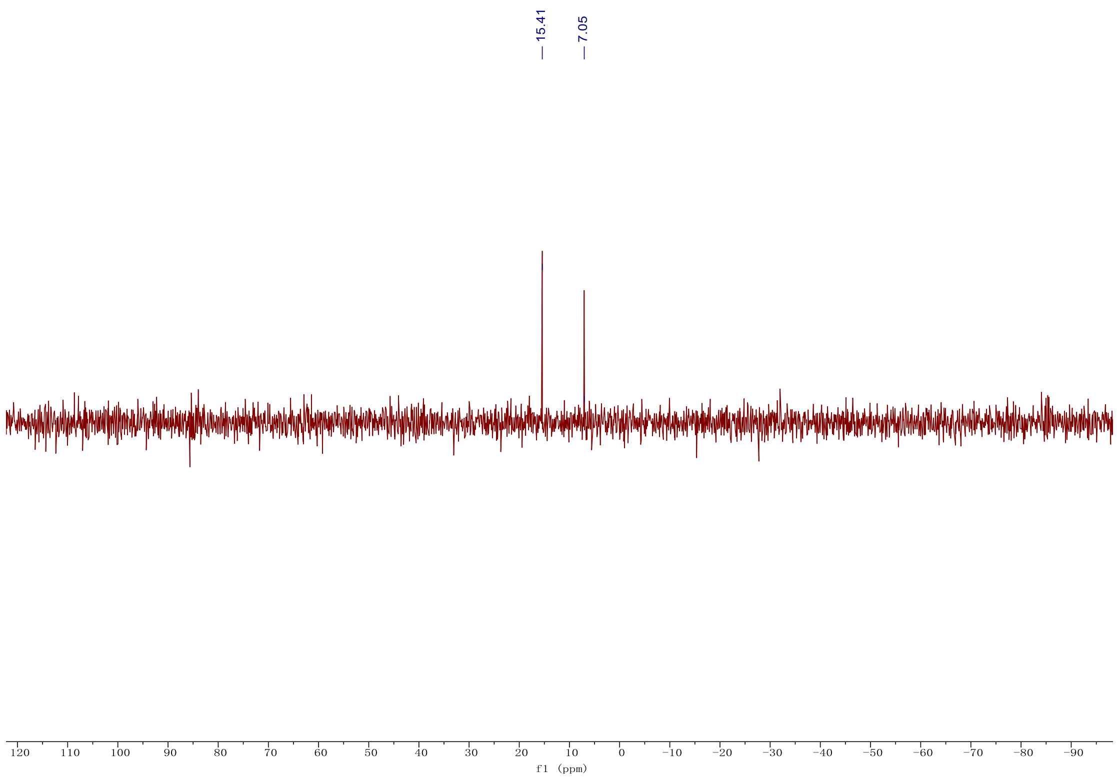


Figure S13. $^{29}$Si NMR spectrum of $\{PhC(N^tBu)_2Si\}_2Fe(C_6H_6)(N_3SiMe_3)$ (**4**) (99 MHz, $C_6D_6$).

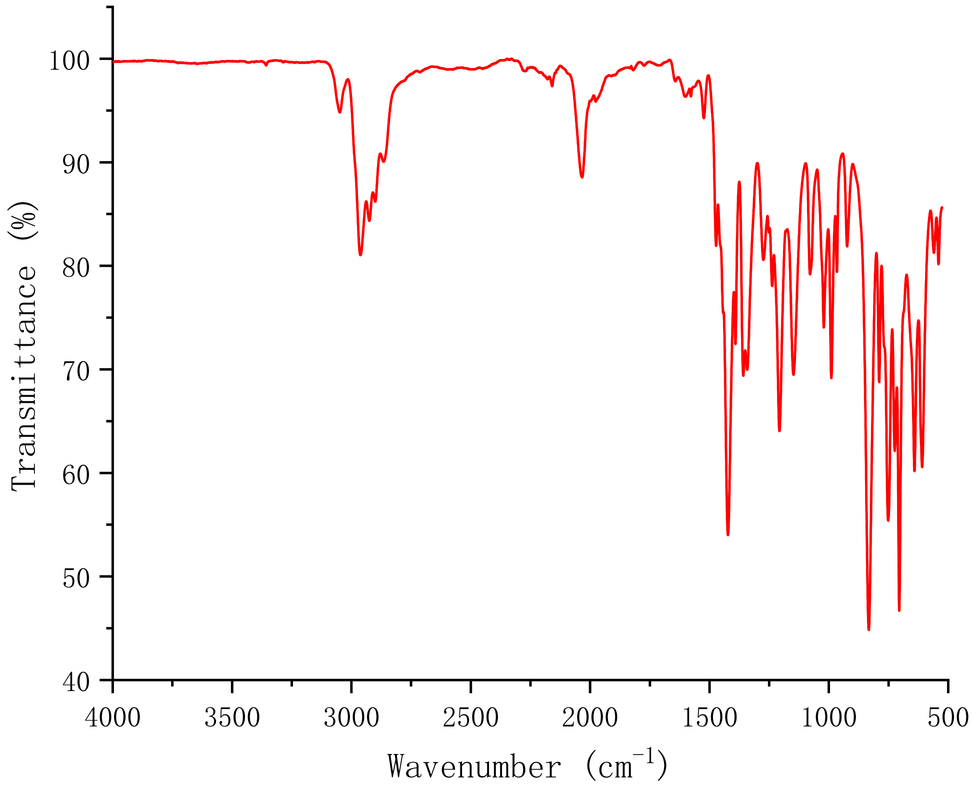


Figure S14. IR spectrum of $\{PhC(N^tBu)_2Si\}_2Fe(C_6H_6)(N_3SiMe_3)$ (**4**).

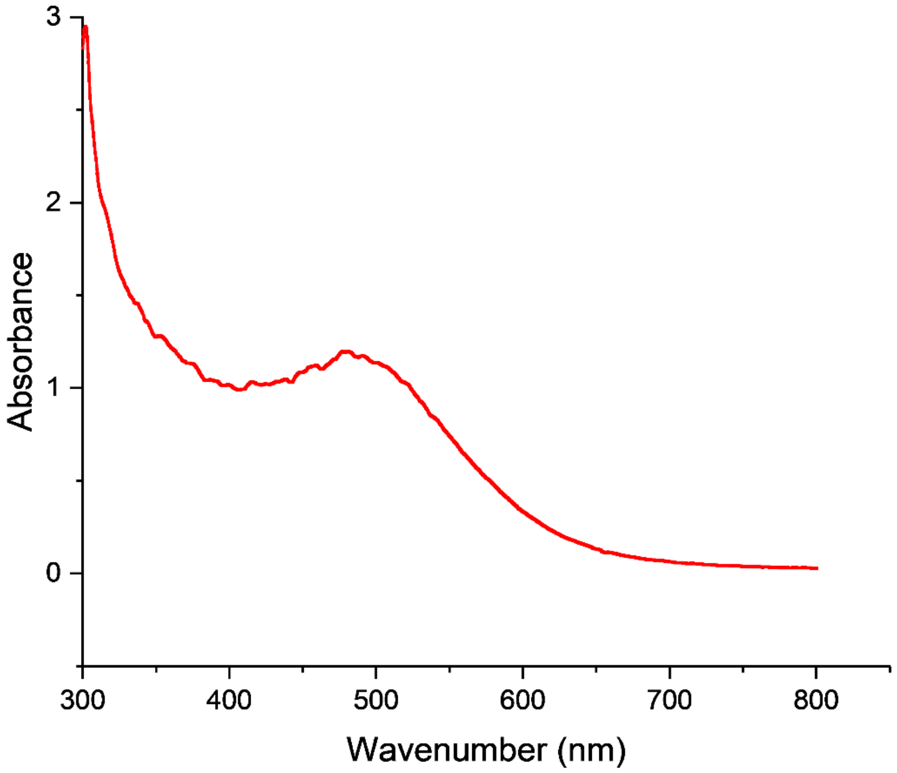


Figure S15. UV-vis spectrum of $\{PhC(N^tBu)_2Si\}_2Fe(C_6H_6)(N_3SiMe_3)$ (**4**).

**{PhC(N*^t*Bu)$_2$Si}$_2$Fe(C$_6$H$_6$)(NSiMe$_3$) (5).**

$TMSN_3$ (7.5 μL, 0.153 mmol) was added by pipette to a solution of **3** {PhC(N$^t$Bu)$_2$Si}$_2$Fe(C$_6$H$_6$) (36 mg, 0.055 mmol) in a J-Young tube or Schlenk tube with stirring at 80 °C. After 90 minutes heating, the product was isolated (Yield: 20.3 mg, 50%) as purple-red solid by washing with cold pentane after removing all solvents and other volatile materials. At shorter reaction times, this product is accompanied by complex **4** {PhC(N$^t$Bu)$_2$Si}$_2$Fe(C$_6$H$_6$)(N$_3$SiMe$_3$), according to NMR spectroscopy. X-ray quality crystals were collected by dissolving the obtained solid in benzene solution and slow evaporation of the solution at room temperature.

$^1$H NMR (500 MHz, benzene-*d*$_6$) δ 7.36 (s, 1H, Ar-H), 7.31 (d, 3H, Ar-H), 7.01~6.99 (m, 4H, Ar-H), 6.96 (m, 2H, Ar-H), 5.12 (s, 6H, benzene-H), 1.43 (s, 36H, N$^t$Bu-H), 0.39 (s, 9H, Si(CH$_3$)$_3$).

$^{13}$C NMR (126 MHz, benzene-*d*$_6$, ppm) δ 169.61 (N*C*N), 133.69, 129.94, 128.47, 127.67 (133.69~127.67: Ph), 75.31 (Fe-benzene), 53.61 (*C*Me$_3$), 31.75 (*C*H$_3$), 3.79 (Si(*C*H$_3$)$_3$).

$^{29}$Si NMR (99 MHz, benzene-*d*$_6$, ppm) δ 7.42, −20.71.

UV-Vis (THF, λ(nm) (ε, M$^{-1}$cm$^{-1}$)): 530 (592).

IR-ATR (cm$^{-1}$): 3357 (VW), 2961 (w), 2029 (S), 1597 (W), 1473 (VW), 1419 (S), 1392 (W), 1358 (m), 1237 (W), 1204 (S), 1074 (W), 1019 (S), 924(VW), 832 (S), 787 (VW), 746 (S), 704 (S), 649 (VW), 641 (VW), 609 (W).

Anal. Calcd for $C_{39}H_{61}FeN_5Si_3$: C, 63.30; H, 8.31; N, 9.46. Found: C, 60.19; H, 7.99; N, 8.82. Due to the formation of silicon carbide the carbon values in the elemental analyses were consistently too low for the disilylene Fe compounds reported in this paper.

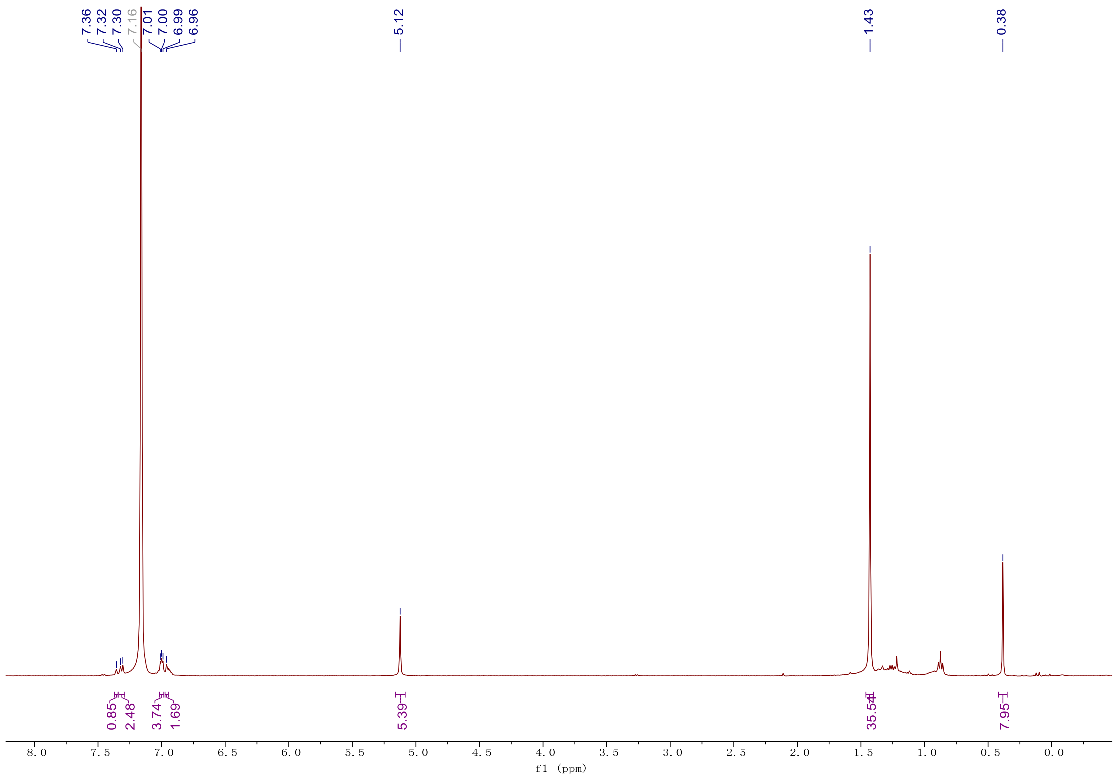


Figure S16. $^{1}$H NMR spectrum of {PhC(N$^t$Bu)$_2$Si}$_2$Fe(C$_6$H$_6$)(NSiMe$_3$) (**5**) (500 MHz, C$_6$D$_6$).

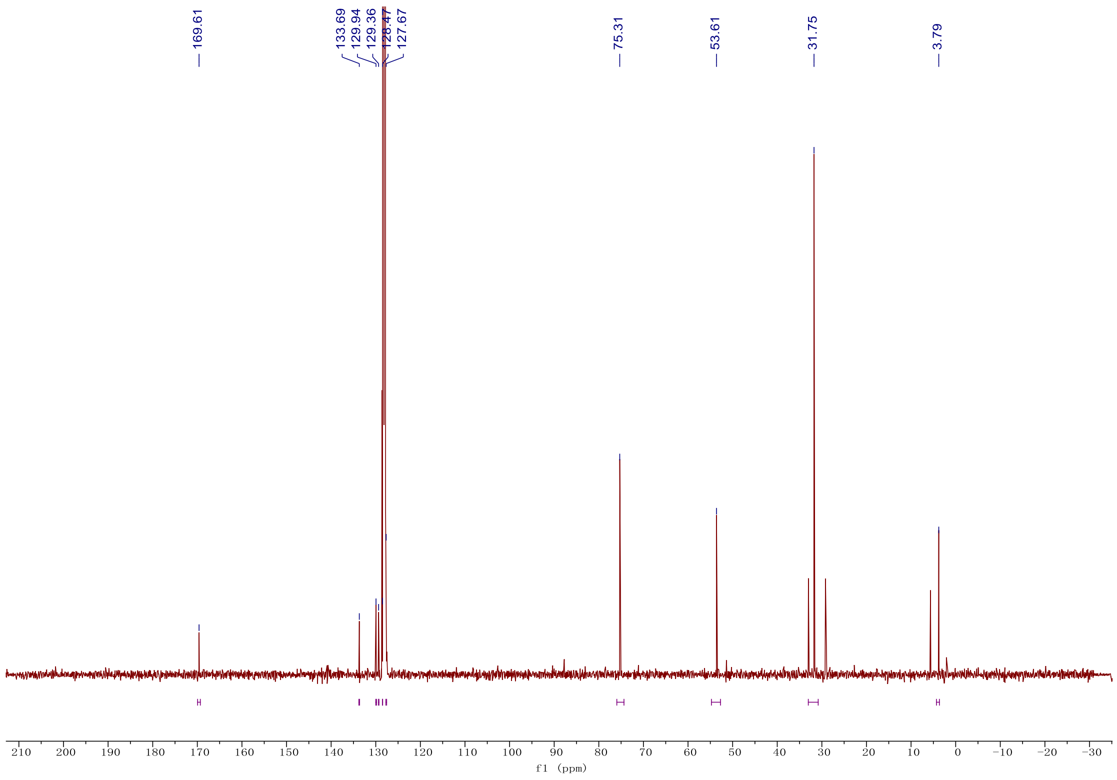


Figure S17. $^{13}$C NMR spectrum of {PhC(N$^t$Bu)$_2$Si}$_2$Fe(C$_6$H$_6$)(NSiMe$_3$) (**5**) (126 MHz, C$_6$D$_6$).

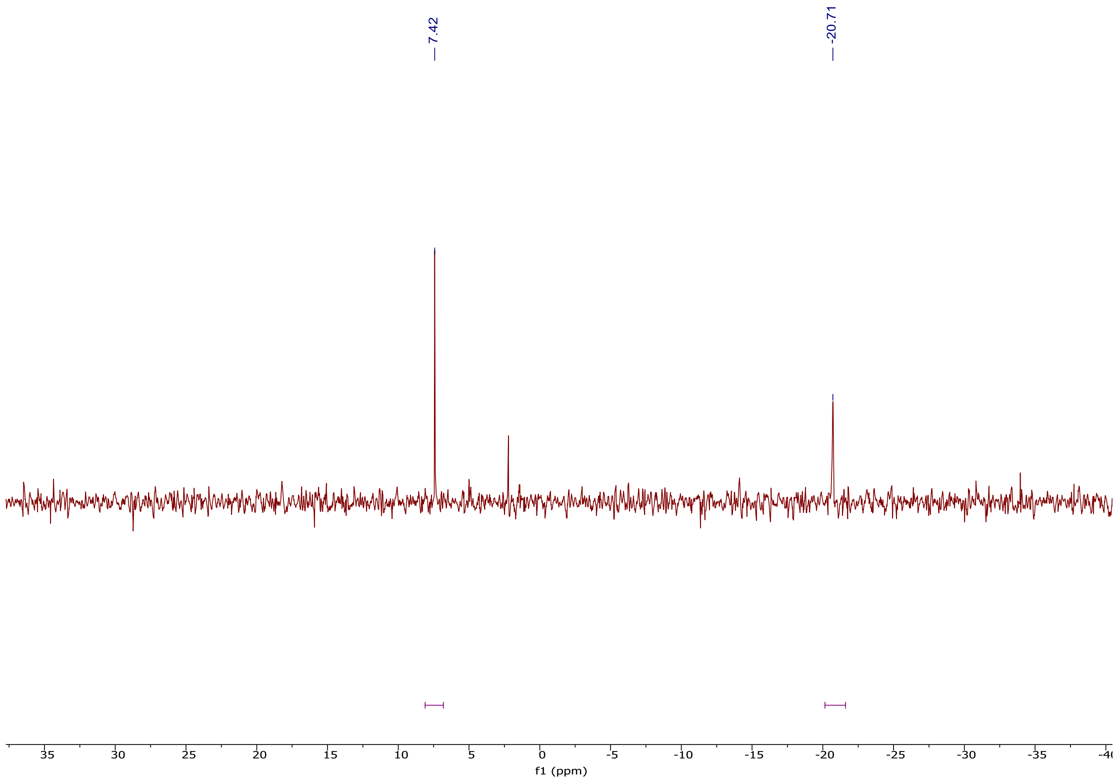


Figure S18. $^{29}$Si NMR spectrum of {PhC(N$^t$Bu)$_2$Si}$_2$Fe($C_6H_6$)($NSiMe_3$) (**5**) (99 MHz, $C_6D_6$).

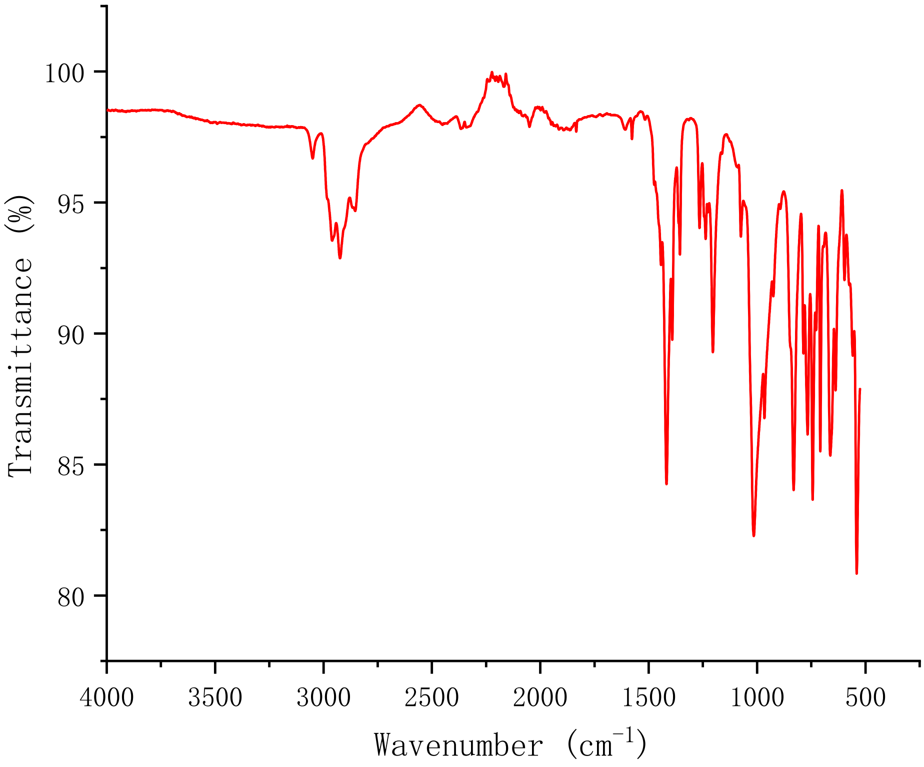


Figure S19. IR spectrum of {PhC(N$^t$Bu)$_2$Si}$_2$Fe($C_6H_6$)($NSiMe_3$) (**5**).

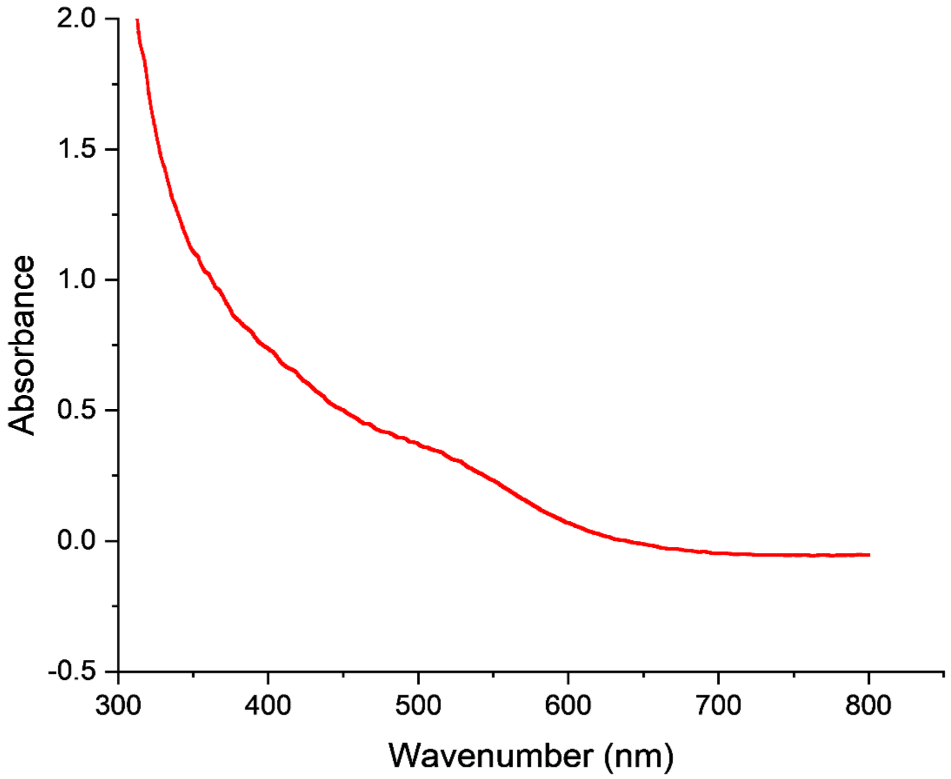

Figure S20. UV-vis spectrum of {PhC(N$^t$Bu)$_2$Si}$_2$Fe($C_6H_6$)($NSiMe_3$) (**5**).

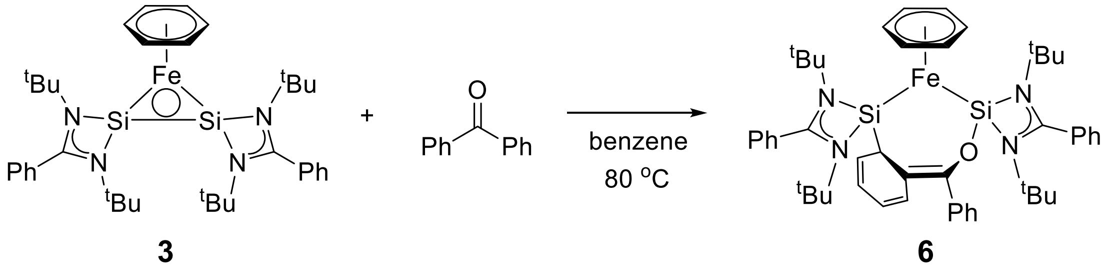


**{PhC(N$^t$Bu)$_2$Si}$_2$Fe(C$_6$H$_6$)(Ph$_2$CO) (6).**

Benzophenone (437 μL of a 100-mg-in-10-mL stock solution in benzene) was added dropwise to a solution of **3** (15.6 mg) in benzene (5 mL) in a J-Young tube or Schlenk tube at 80 °C with stirring. The product was isolated as dark greenish-black solid by washing with cold pentane after removing all solvents and other volatile materials. Yield: 12 mg, 60 %. X-ray quality crystals were collected by dissolving solid in diethyl ether solution and then placing the sample in a −30 °C freezer.

$^1$H NMR (500 MHz, benzene-$d_6$) δ 8.36 (d, 0.5H, Ar-H), 7.76 (d, 2H, Ar-H), 7.52 (d, 1H, Ar-H), 7.31 (d, 0.5H, Ar-H), 7.25 (t, 2H, Ar-H), 7.06~6.95 (m, 9H, Ar-H), 6.98(d,1H, de-ArCH), 6.17 (dd, 1H, de-ArCH), 5.86 (m, 1H, de-ArCH), 5.65 (dd, 1H, de-ArCH), 4.90 (d,1H, de-ArCH), 5.07 (s, 6H, benzene-H), 1.59/ 1.45/ 1.31/ 0.97 (s, 9H, N$^t$Bu-H).

$^{13}$C NMR (126 MHz, benzene-$d_6$, ppm) δ 170.46 (N*C*N), 167.56 (N*C*N), 159.33~122.22 (18 signals; 6 C + 18 C; CCCHCHCHCH + C of 3 Ph), 78.46 (Fe-benzene), 53.23 (*C*Me$_3$), 53.14 (*C*Me$_3$), 52.67 (*C*Me$_3$), 52.38 (*C*Me$_3$), 32.12 (*C*H$_3$), 32.01 (*C*H$_3$), 31.15 (*C*H$_3$), 31.09 (*C*H$_3$).

$^{29}$Si NMR (99 MHz, benzene-$d_6$, ppm) δ 65.57, 33.66.

UV-Vis (THF, λ(nm) (ε, M$^{-1}$cm$^{-1}$)): 350 (4785), 435 (2782).

IR-ATR (cm$^{-1}$): 3052 (VW), 2965 (W), 2962 (VW). 1647 (VW), 1596 (VW), 1472 (W), 1421 (S), 1390 (m), 1358 (m), 1264 (m), 1204 (S), 1106 (VW), 1072 (W), 1009 (W), 970 (W), 923 (W), 865 (W), 790 (m), 745 (S), 722 (VW), 699 (VS), 607 (S), 544 (S).

Anal. Calcd for $C_{49}H_{62}FeN_4OSi_2$: C, 70.48; H, 7.48; N, 6.71. Found: C, 65.07; H,7.09; N, 6.15. Due to the formation of silicon carbide the carbon values in the elemental analyses were consistently too low for the disilylene Fe compounds reported in this paper.

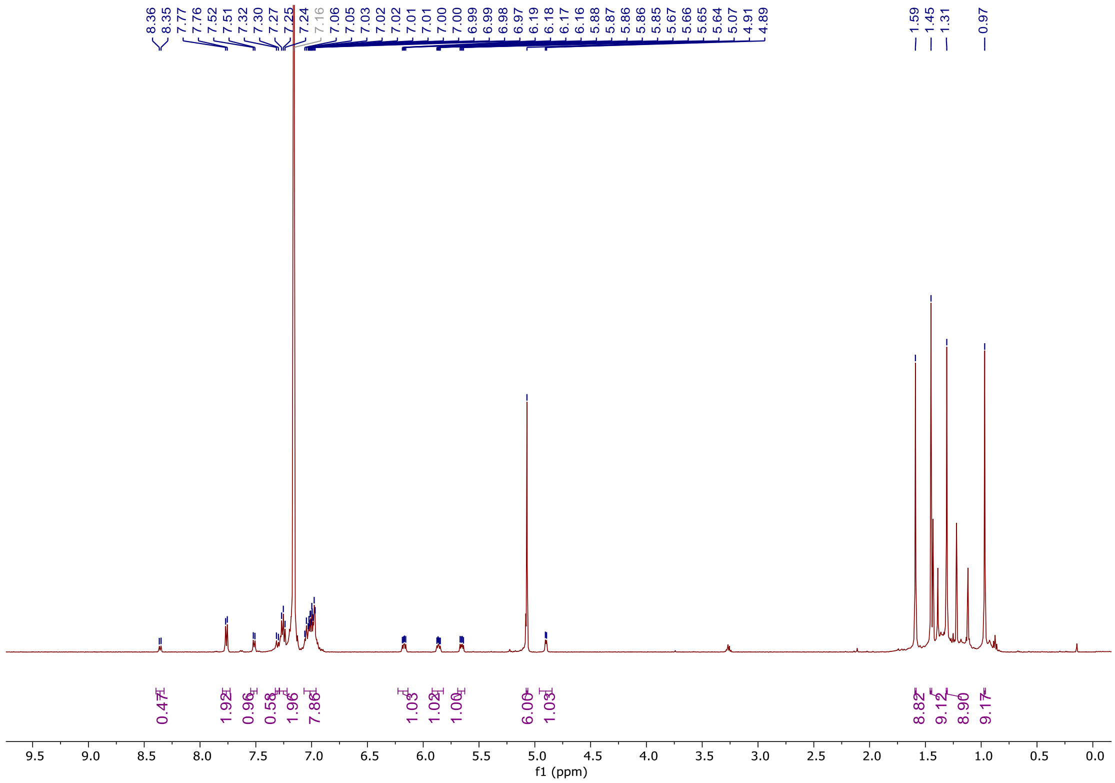


Figure S21. $^{1}$H NMR spectrum of {PhC(N$^{t}$Bu)$_2$Si}$_2$Fe(C$_6$H$_6$)(Ph$_2$CO) (**6**) (500 MHz, C$_6$D$_6$).

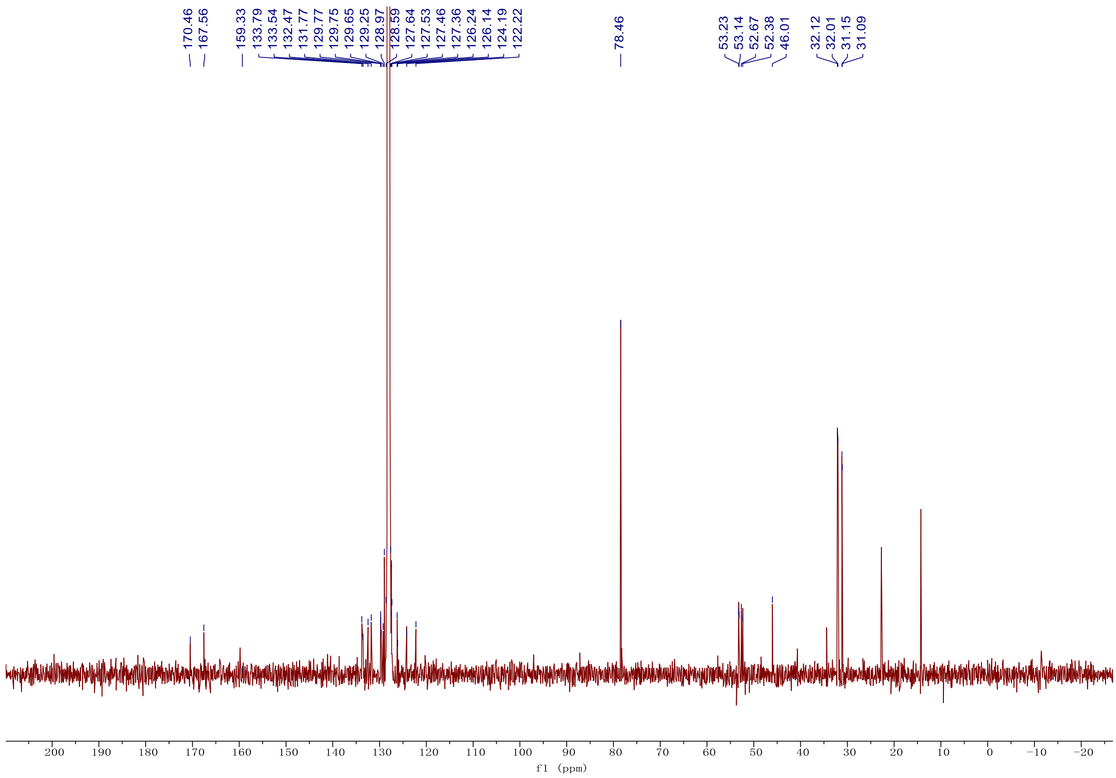


Figure S22. $^{13}$C NMR spectrum of {PhC(N$^{t}$Bu)$_2$Si}$_2$Fe(C$_6$H$_6$)(Ph$_2$CO) (**6**) (126 MHz, C$_6$D$_6$).

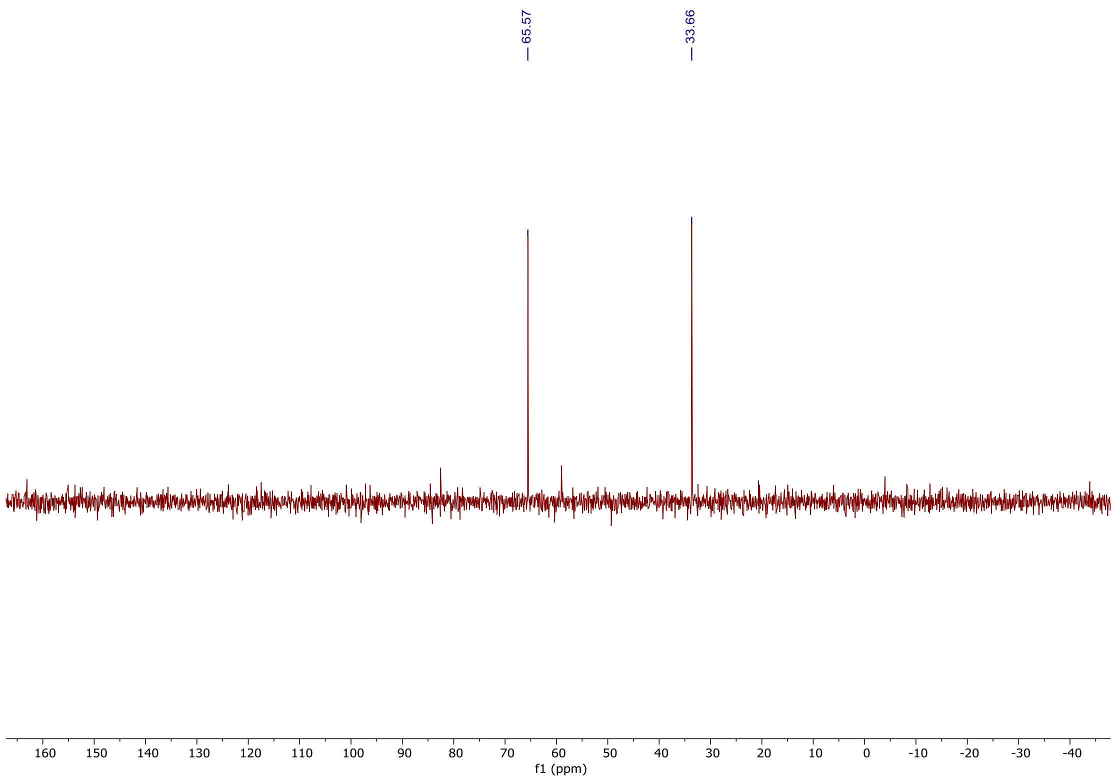


Figure S23. $^{29}$Si NMR spectrum of {PhC(N$^t$Bu)$_2$Si}$_2$Fe(C$_6$H$_6$)(Ph$_2$CO) (**6**) (99 MHz, C$_6$D$_6$).

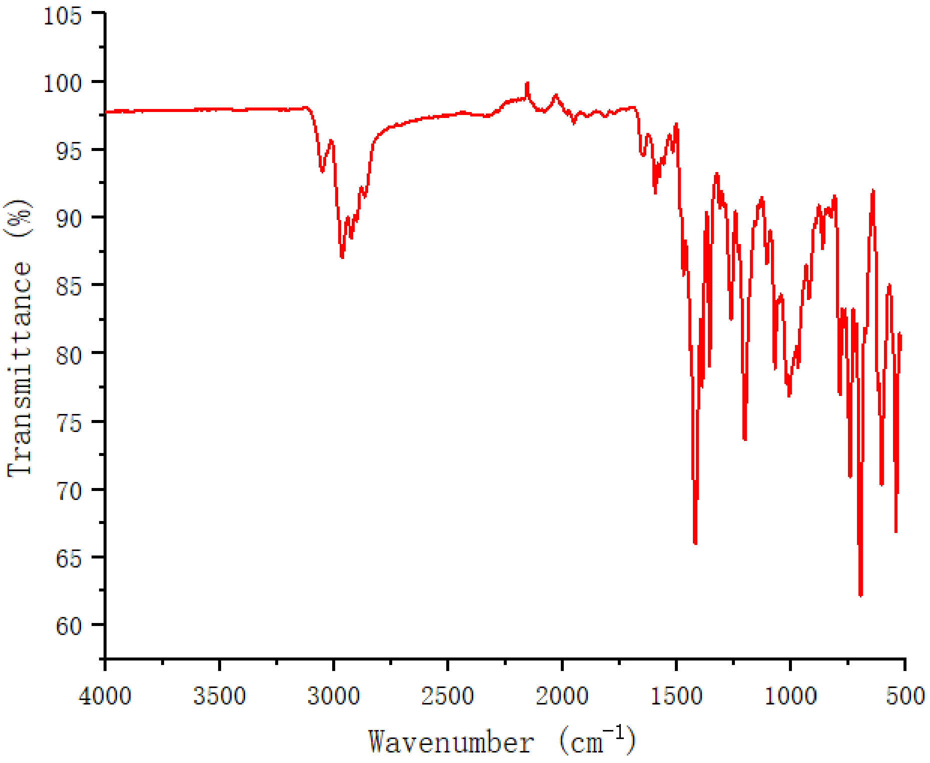


Figure S24. IR spectrum of {PhC(N$^t$Bu)$_2$Si}$_2$Fe(C$_6$H$_6$)(Ph$_2$CO) (**6**).

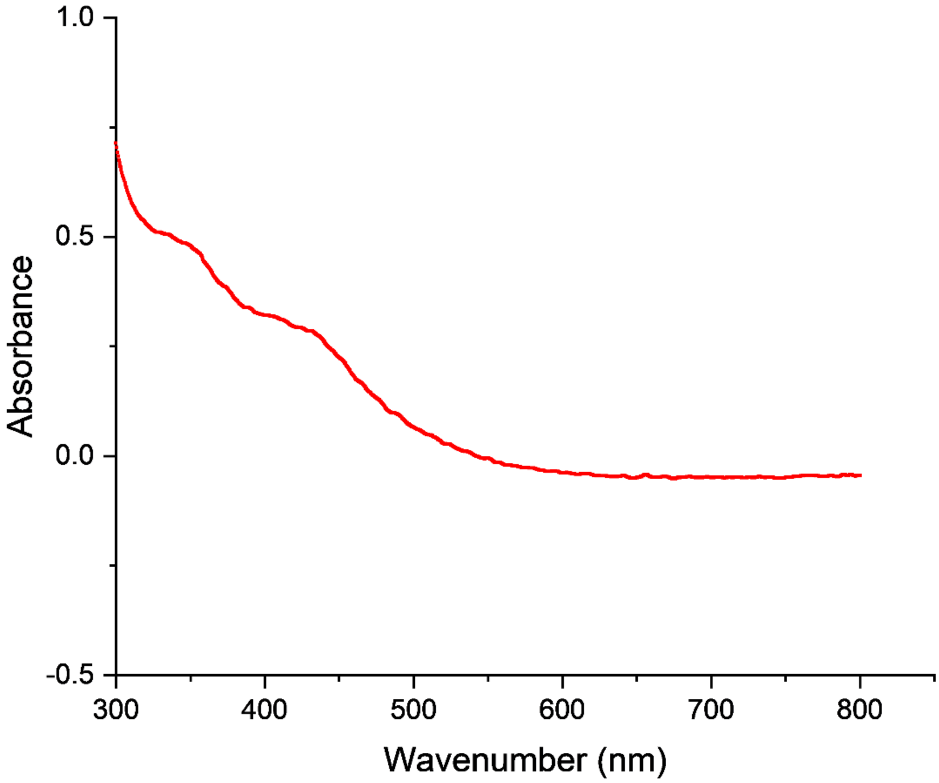


Figure S25. UV-vis spectrum of {PhC(N$^t$Bu)$_2$Si}$_2$Fe($C_6H_6$)($Ph_2CO$) (**6**).

# Reaction of 3 with TMS-azide followed by NMR

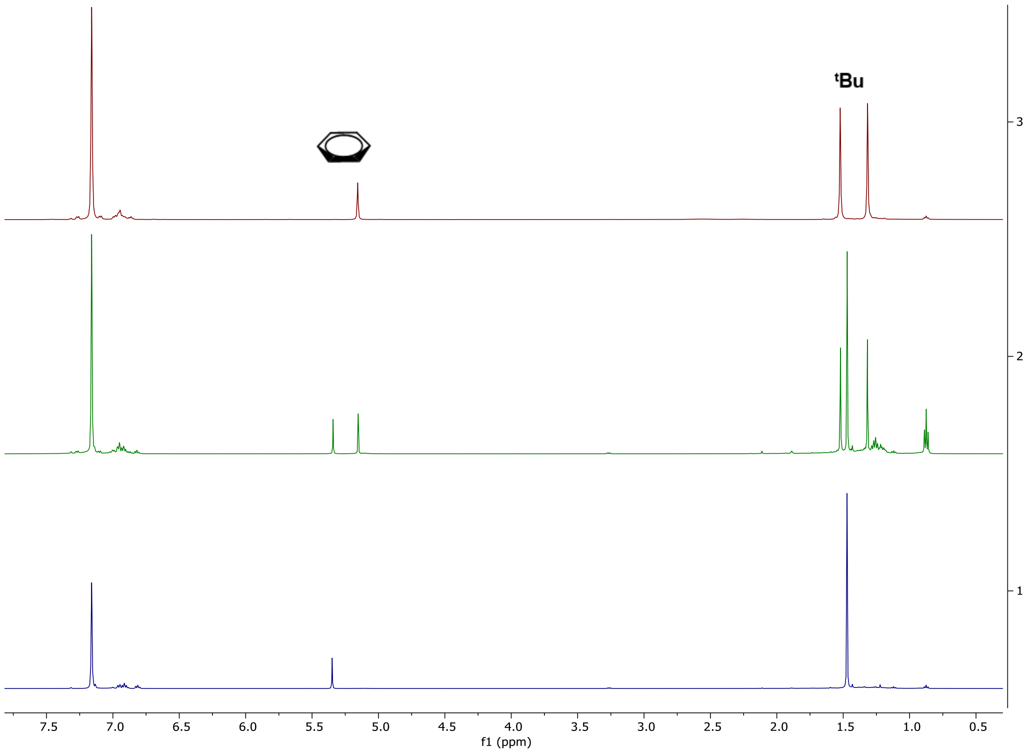


Figure S26. Reaction process of compound **3** under the $^{1}$H NMR monitoring experiment.

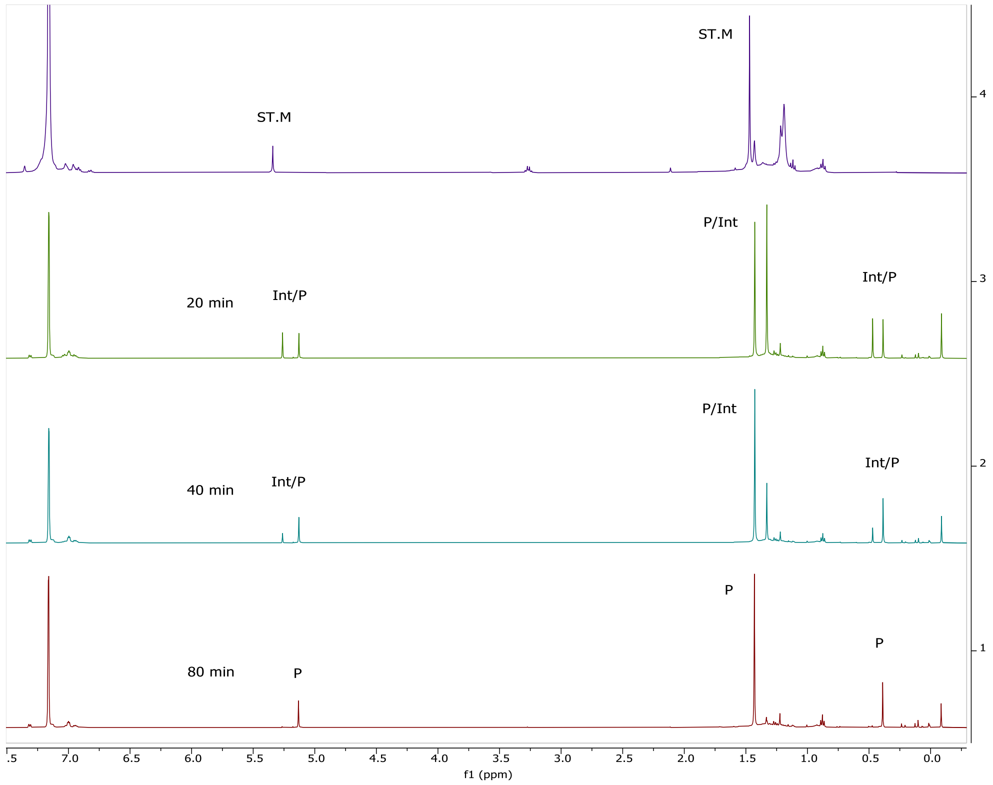


Figure S27. Reaction process of compound **3** with $TMSN_3$ to form compound **4** (Int) and compound **5** (P) via $^{1}$H NMR monitoring.

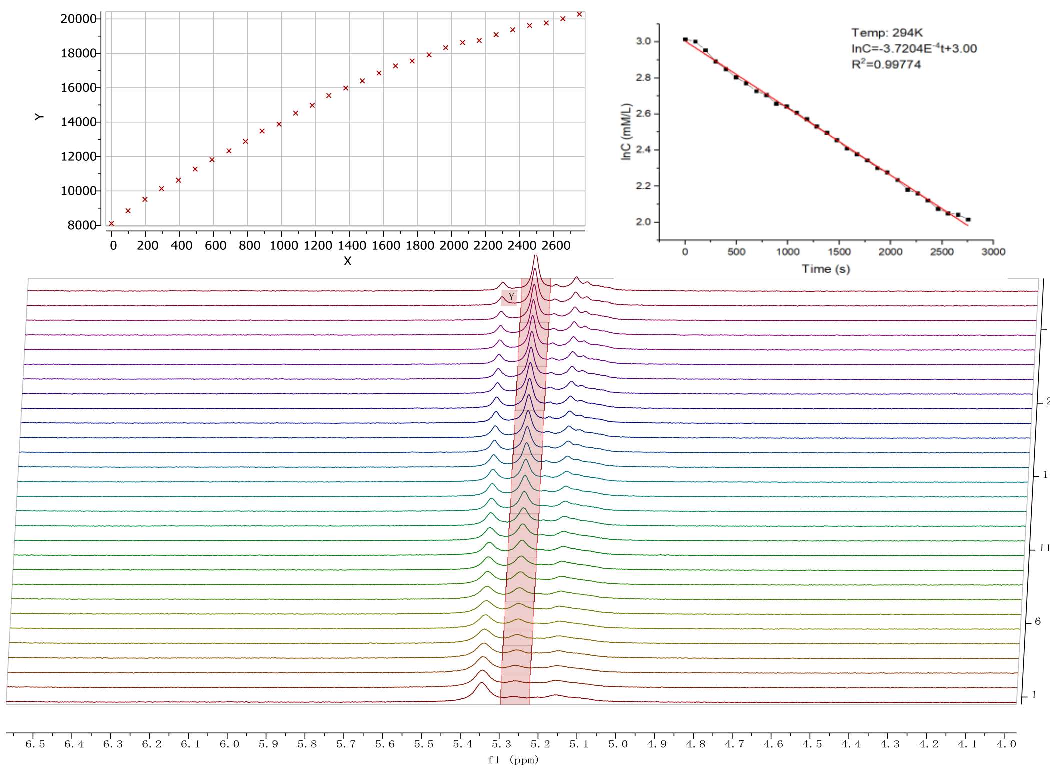


Figure S28. $^{1}$H NMR monitoring of the reaction progress from **3** to **4** at 294 K.

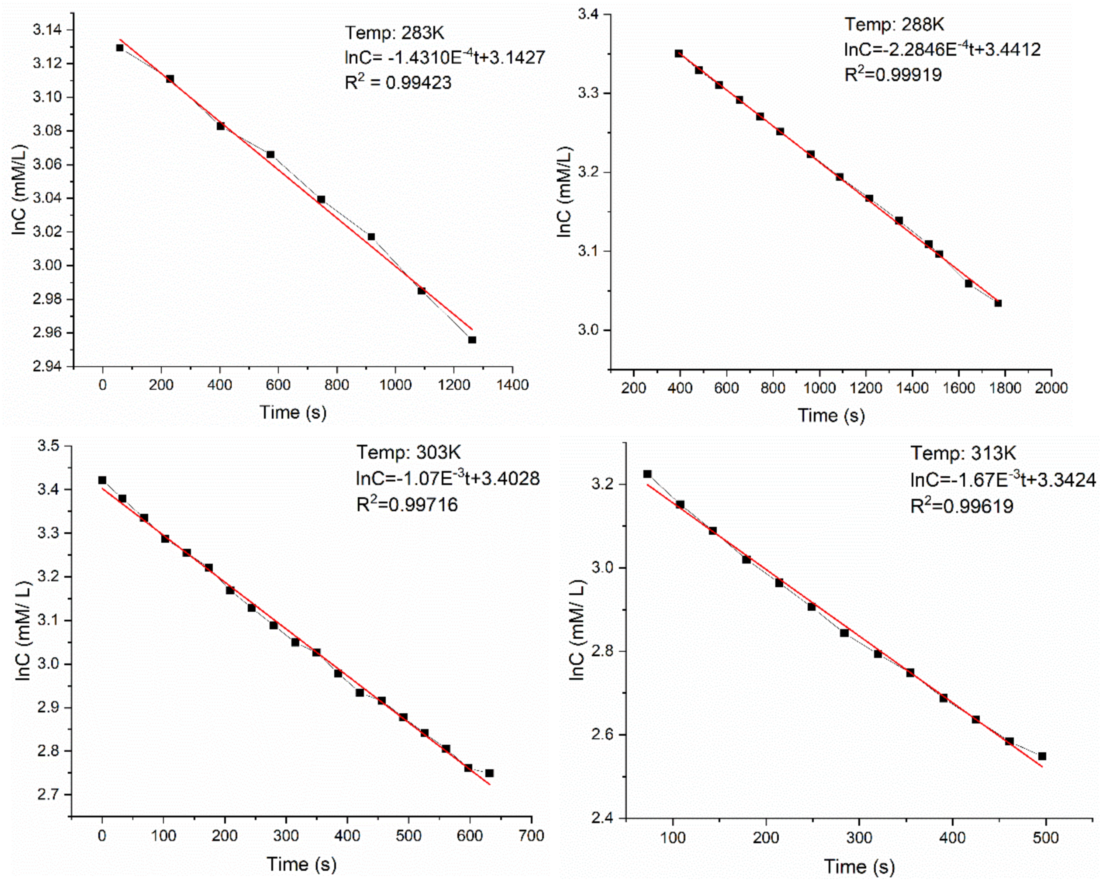


Figure S29. Reaction progress monitoring by $^{1}$H NMR for conversion of **3** into intermediate **4** at different temperatures: 283 K (top left), 288 K (top right), 303 K (bottom left), 313 K (bottom right).

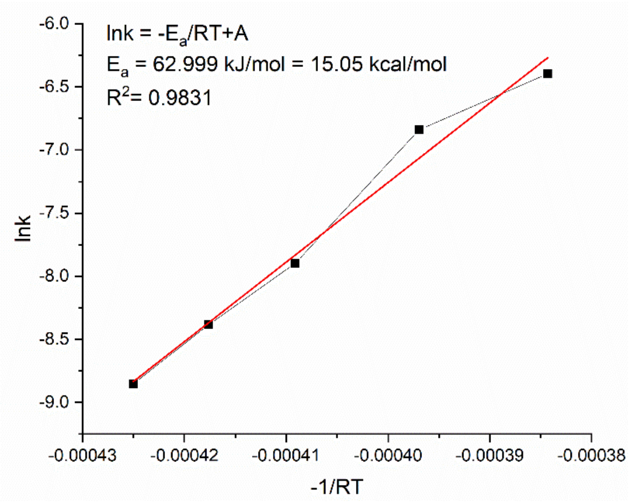


Figure S30. Arrhenius plot for conversion of compound **3** into intermediate **4**

# EDA and DFT calculations

**Computational details:**

Geometries were fully optimized as minima or transition states using the Turbomole program package,[S7] coupled to the PQS Baker optimizer[S8] via the BOpt package.[S9] We used unrestricted ri-DFT-D3 calculations at the B3LYP level,[S10] in combination with the def2-TZVP basis set[S11] and a small (m4) grid size. Grimme's dispersion corrections[S12] (version 3, disp3, 'zero damping') were used to include Van der Waals interactions. All minima (no imaginary frequencies) and transition states (one imaginary frequency) were characterized by calculating the Hessian matrix. Thermochemical parameters such as the zero-point energy (ZPE), Gibbs free energy and gas-phase thermal corrections (entropy and enthalpy, 298 K, 1 bar) were obtained from these analyses. The nature of the transition states was confirmed by following the intrinsic reaction coordinate (IRC). The relative free energies ($\Delta G°_{298K}$ in kcal·mol$^{-1}$ ) obtained from these calculations are reported in the main text. For every transition state, the imaginary eigenvalue was followed in both directions to confirm its connection to the relative reactant and product states. A separate archive file is provided, containing an Excel sheet with free energies ($\Delta G°_{298K}$) and negative eigenvalues of the transition states, and all optimized geometries. Optimized geometries of all stationary states and transition states are supplied in .pdb and .xyz format.

The energy decomposition analysis (EDA)[S13] has been performed on the TZ2P/OPBE[S14] optimized geometry constrained to $C_{2v}$ symmetry (+3.9 kcal·mol$^{-1}$) since it is most informative if the orbital interactions can be dissected by different irreducible representation. The total bonding energy of −57.6 kcal mol$^{-1}$ consists of the preparation energy of the fragments into the geometry and electronic state they possess in the complex as well as their orbital interactions and the steric interaction (+31.7 kcal·mol$^{-1}$), which is the balance between the Pauli repulsion of the electron densities of the interacting fragments and the attraction of the electron density of one fragment and the nuclei of the other and vice versa. The contribution of the orbital interaction is usually dominated by the stabilization of the high lying occupied orbitals of one fragment by the low-lying empty orbitals of the other fragment (donor-acceptor interaction) but also contains the mixing of occupied and empty orbitals at the same fragment (polarization).

The Fe(benzene) fragment (+42.3 kcal·mol$^{-1}$) has been calculated in the singlet state (Fe(0)-$d^8$) with a doubly occupied $d_{yz}$ (perpendicular to the Si−Fe−Si plane) and an empty $d_{xz}$ orbital. The electronic structure of the Si(I)−Si(I) fragment (+52.1 kcal·mol$^{-1}$) is best described as having a Si−Si single bond with a lone pair on each silicon and an empty π-orbital between them. The two lone pairs are forming a bonding (A1) and antibonding (B1) combination. The former donate electron density into the empty $d_{xz}$ (A1: −30.3 kcal·mol$^{-1}$, 0.4 e; B1: −74.4 kcal·mol$^{-1}$, 0.8 e) orbital at iron. The metal itself donates charge density back from the occupied $d_{yz}$ orbital into the empty π-symmetric orbital of the Si−Si bond (B2: −67.8 kcal·mol$^{-1}$, 0.9 e), giving rise to a considerable degree of π-backbonding.

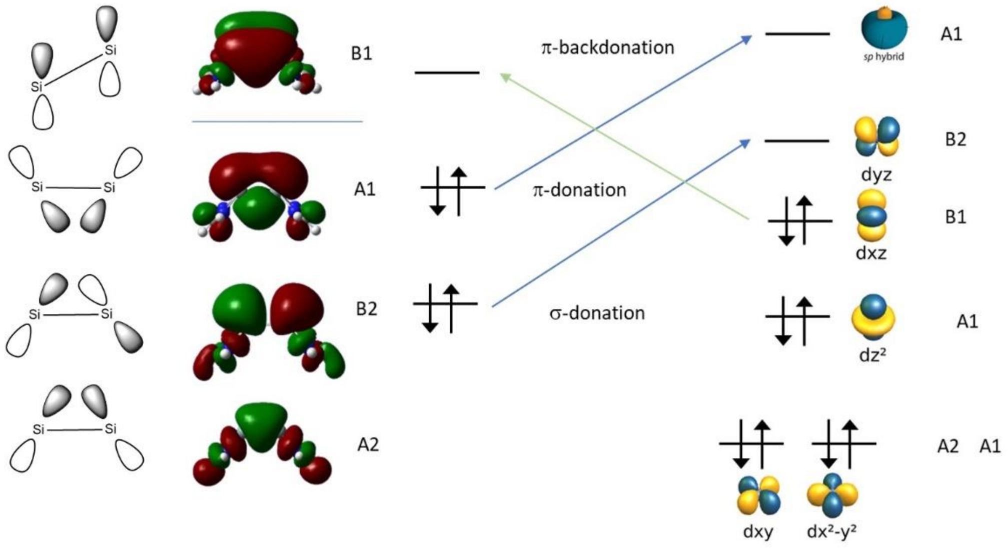


Figure S31. Results of the EDA analysis (interaction between Si(I)–Si(I) and Fe).

The Nucleus Independent Chemical Shift (NICS) [S15] was used as a diagnostic probe for quantitative measure for aromaticity at the B3LYP/6-11+G(d,p) level.[S16,S17] The NICS index is the negative value of the isotropic magnetic shielding computed at ring centers and points above and below[S18] in and around the molecule.[S19] We used the refined method by Stanger, following the out of plane component to the shielding tensor along a trajectory orthogonal to the plane of the ring ($NICS_{zz}$),[S20] as this can be used to characterize whether an inorganic system is aromatic, non-aromatic or anti-aromatic.[S22] A minimum in $NICS_{zz}$ (out of plane eigenvalue) at a nonzero r value is indicative of aromatic π-delocalization.[S23] Arguably, this is due to the π-electron density above (and below) the aromatic ring. A similar behaviour is calculated for the benzene ring in **3** (scanning the axis opposite to iron), again a minimum is found about 1 Å above the ring for the out of plane (zz) component (red line, left figure below). For the Fe–Si–Si ring in **3** (Figure S33), there is no real minimum for the out of plane component (red line) (note that the zz-eigenvalue of –60.4 at 0.2Å is slightly lower than that of –59.8 at the centre).

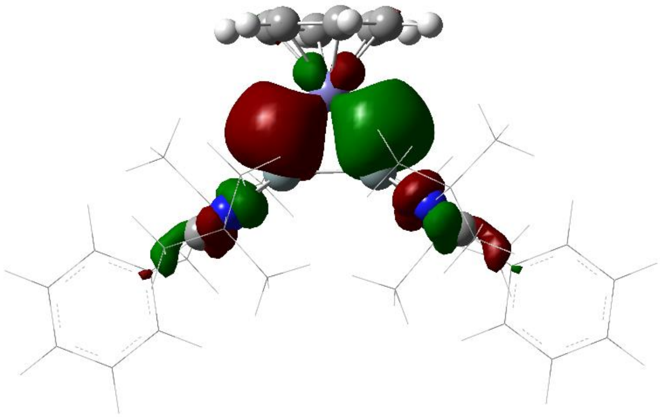

Figure S32. Representation of the HOMO-5 in complex **3**.

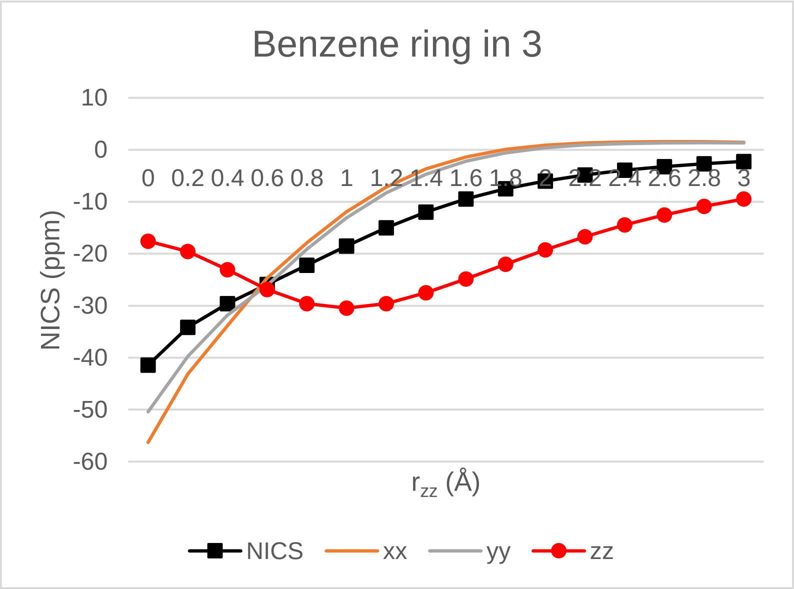


Figure S33. NICS perpendicular to the ring plane of the benzene ring in compound **3**.

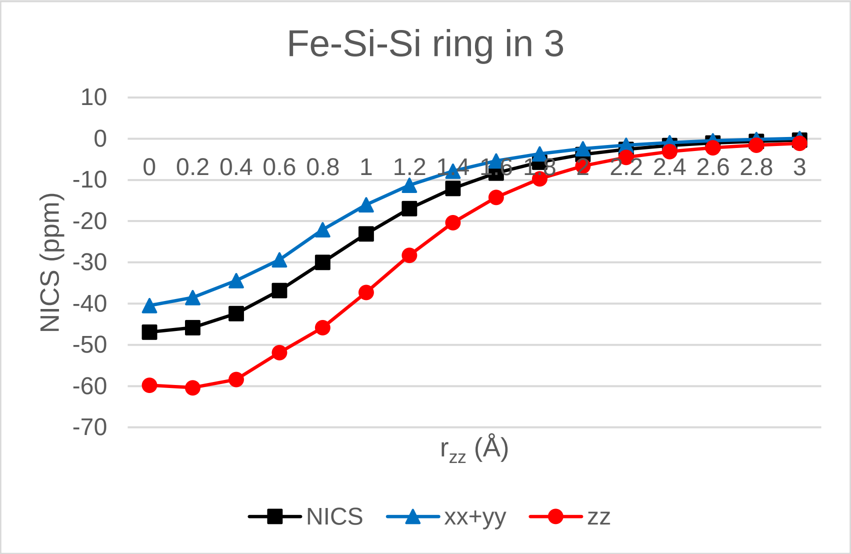


Figure S34. NICS perpendicular to the ring plane of the Fe-Si-Si ring in compound **3**.

Therefore we resorted to the CMO analysis of the NICS(0), which separates the total shieldings into contributions from canonical molecular orbitals.[S21] Indeed, there is a sizable contribution of −13.8 ppm from the Fe-$d_{xy}$ orbital (HOMO-5) that is pointing toward the center of the three membered ring. More importantly, the major contribution of −16.6 ppm originates from the delocalized π-orbital shown in Fig. 3 (main text) proving definitely the 2π-aromaticity of the $Si_2Fe$-three-membered ring in complex **3**. It was also established that the shapes of the NICS-scan profiles provide a clear picture of the type of the ring current in aromatic and antiaromatic systems

Optimized coordinates

Compound **3**

95

E= 3463,66282

| | | | |
|---|---|---|---|
| Fe | 10.3351558 | 5.6647774 | 11.8737848 |
| Si | 9.2315091 | 3.7691544 | 11.7238373 |
| N | 8.2179792 | 2.8964234 | 10.3951047 |
| N | 8.1085331 | 2.4629532 | 12.4949055 |
| C | 7.6121262 | 2.1155942 | 11.3071703 |
| C | 7.6698782 | 3.3618082 | 9.1008354 |
| C | 6.4637921 | 4.2789570 | 9.3688814 |
| C | 7.2568080 | 2.2033103 | 8.1799861 |
| C | 8.7709090 | 4.1658316 | 8.3993657 |
| C | 8.1688582 | 1.7144469 | 13.7641206 |
| C | 8.4341272 | 2.7599946 | 14.8536881 |
| C | 6.8652141 | 0.9774390 | 14.1015066 |
| C | 9.3292312 | 0.7035906 | 13.7001415 |
| C | 6.6434094 | 1.0321192 | 11.0209545 |
| C | 5.2753247 | 1.2117313 | 11.2208302 |
| C | 4.3847454 | 0.1858481 | 10.9303274 |
| C | 4.8549927 | -1.0308270 | 10.4456184 |
| C | 6.2201880 | -1.2167970 | 10.2496354 |
| C | 7.1104600 | -0.1886228 | 10.5317068 |
| C | 9.1397526 | 7.2256122 | 11.1238140 |
| C | 9.0917043 | 7.2189544 | 12.5365966 |
| C | 10.2861143 | 7.2025952 | 13.2938635 |
| H | 5.6624801 | 3.7307091 | 9.8668589 |
| H | 6.0687047 | 4.6790293 | 8.4324063 |
| H | 6.7605747 | 5.1131528 | 10.0067935 |
| H | 8.0692513 | 1.4808615 | 8.0863227 |
| H | 7.0358716 | 2.5996311 | 7.1865854 |
| H | 6.3708191 | 1.6806577 | 8.5346595 |
| H | 9.1953853 | 4.9147532 | 9.0693335 |
| H | 8.3593613 | 4.6640622 | 7.5194523 |
| H | 9.5746556 | 3.5084266 | 8.0701209 |
| H | 9.2879259 | 3.3802730 | 14.5892148 |
| H | 8.6328075 | 2.2710443 | 15.8095214 |
| H | 7.5695012 | 3.4159852 | 14.9702153 |
| H | 6.0086185 | 1.6499226 | 14.0382598 |
| H | 6.9297887 | 0.6047769 | 15.1258302 |
| H | 6.6849241 | 0.1266062 | 13.4476925 |
| H | 9.1646465 | -0.0091984 | 12.8891201 |
| H | 9.3994037 | 0.1452273 | 14.6370367 |
| H | 10.2720226 | 1.2206076 | 13.5230106 |

| | | | |
|---|---|---|---|
| H | 4.9125164 | 2.1551735 | 11.6076975 |
| H | 3.3233036 | 0.3354458 | 11.0838220 |
| H | 4.1603660 | -1.8304858 | 10.2209753 |
| H | 6.5911502 | -2.1618286 | 9.8727517 |
| H | 8.1724239 | -0.3233710 | 10.3690867 |
| H | 8.2215673 | 7.1597960 | 10.5569136 |
| H | 8.1379723 | 7.1353053 | 13.0378685 |
| H | 10.2471624 | 7.1515040 | 14.3731097 |
| Si | 11.4387734 | 3.7691333 | 12.0237883 |
| N | 12.4522738 | 2.8963836 | 13.3525329 |
| N | 12.5617060 | 2.4628549 | 11.2527562 |
| C | 13.0580890 | 2.1154973 | 12.4405000 |
| C | 13.0003941 | 3.3618148 | 14.6467716 |
| C | 14.2063528 | 4.2791123 | 14.3786763 |
| C | 13.4136648 | 2.2033741 | 15.5675937 |
| C | 11.8992651 | 4.1656795 | 15.3482891 |
| C | 12.5014079 | 1.7143558 | 9.9835078 |
| C | 12.2359681 | 2.7599474 | 8.8939911 |
| C | 13.8051492 | 0.9775570 | 9.6459830 |
| C | 11.3411527 | 0.7033136 | 10.0474787 |
| C | 14.0267808 | 1.0320138 | 12.7267514 |
| C | 15.3948620 | 1.2115326 | 12.5267869 |
| C | 16.2854074 | 0.1856285 | 12.8173187 |
| C | 15.8151291 | -1.0309716 | 13.3021553 |
| C | 14.4499375 | -1.2168433 | 13.4982373 |
| C | 13.5596995 | -0.1886527 | 13.2161300 |
| C | 11.5305515 | 7.2256581 | 12.6236780 |
| C | 11.5786002 | 7.2189516 | 11.2108957 |
| C | 10.3841904 | 7.2025443 | 10.4536299 |
| H | 15.0076856 | 3.7309765 | 13.8806097 |
| H | 14.6014704 | 4.6791853 | 15.3151388 |
| H | 13.9094307 | 5.1133019 | 13.7408257 |
| H | 12.6013587 | 1.4807935 | 15.6613619 |
| H | 13.6346752 | 2.5997439 | 16.5609587 |
| H | 14.2997313 | 1.6808740 | 15.2128869 |
| H | 11.4748180 | 4.9146454 | 14.6783575 |
| H | 12.3108285 | 4.6638175 | 16.2282458 |
| H | 11.0956132 | 3.5080969 | 15.6773978 |
| H | 11.3821207 | 3.3801300 | 9.1585197 |
| H | 12.0373391 | 2.2710124 | 7.9381151 |
| H | 13.1005270 | 3.4160593 | 8.7774815 |
| H | 14.6616309 | 1.6501911 | 9.7092905 |
| H | 13.7405569 | 0.6050113 | 8.6216045 |
| H | 13.9855343 | 0.1266729 | 10.2997237 |

| | | | |
|---|---|---|---|
| H | 11.5059147 | -0.0095532 | 10.8584126 |
| H | 11.2709160 | 0.1450943 | 9.1104896 |
| H | 10.3983258 | 1.2202172 | 10.2247749 |
| H | 15.7577027 | 2.1549192 | 12.1398347 |
| H | 17.3468488 | 0.3351425 | 12.6637528 |
| H | 16.5097219 | -1.8306538 | 13.5268157 |
| H | 14.0789412 | -2.1618181 | 13.8752109 |
| H | 12.4977367 | -0.3233262 | 13.3788106 |
| H | 12.4487350 | 7.1599082 | 13.1905721 |
| H | 12.5323262 | 7.1353112 | 10.7096340 |
| H | 10.4231377 | 7.1514005 | 9.3743887 |

$TMSN_3$

16

E= -573,33931

| | | | |
|---|---|---|---|
| Si | 0.0478034 | -0.0105189 | 1.5467950 |
| C | -0.0963802 | -0.0884830 | -0.3187375 |
| H | 0.8927987 | -0.1426692 | -0.7815102 |
| H | -0.6051191 | 0.7944088 | -0.7117395 |
| H | -0.6587082 | -0.9706513 | -0.6352391 |
| C | 0.8991746 | -1.5426172 | 2.2063985 |
| H | 1.9105823 | -1.6284123 | 1.7997715 |
| H | 0.3534382 | -2.4484181 | 1.9302317 |
| H | 0.9746454 | -1.5133137 | 3.2955377 |
| C | 0.8838015 | 1.5547942 | 2.1186729 |
| H | 1.9089249 | 1.6045670 | 1.7430985 |
| H | 0.9198639 | 1.6022840 | 3.2090921 |
| H | 0.3484756 | 2.4365151 | 1.7601224 |
| N | -1.5800453 | 0.0777641 | 2.2391161 |
| N | -2.4546883 | -0.7546694 | 2.1034327 |
| N | -3.3175673 | -1.4815801 | 2.0239572 |

Compound TS**1**

111

E= -4036,97761

| | | | |
|---|---|---|---|
| Fe | 5.2058049 | 17.3971531 | 8.2093694 |
| Si | 3.8439645 | 16.9257711 | 9.8290533 |
| Si | 4.7902081 | 19.0143587 | 9.5274523 |
| Si | 3.5072936 | 18.7502000 | 14.8892969 |
| N | 2.7394025 | 18.4956017 | 12.4211176 |
| N | 3.7692948 | 18.6204712 | 13.1585886 |
| C | 5.1095038 | 18.1950092 | 15.6897653 |
| H | 5.9388320 | 18.8372834 | 15.3859618 |
| H | 5.3608551 | 17.1706451 | 15.4095352 |
| H | 5.0334632 | 18.2399477 | 16.7794351 |

| | | | |
|---|---|---|---|
| C | 3.1443946 | 20.5177122 | 15.4370989 |
| H | 2.8877323 | 20.5411074 | 16.5000618 |
| H | 2.3045670 | 20.9435460 | 14.8821719 |
| H | 4.0118560 | 21.1656202 | 15.2936671 |
| C | 2.0662751 | 17.6651993 | 15.4306765 |
| H | 1.8919616 | 17.7481755 | 16.5068072 |
| H | 2.2438954 | 16.6128523 | 15.1991370 |
| H | 1.1499709 | 17.9697268 | 14.9181052 |
| N | 3.8983534 | 15.6590238 | 11.1961087 |
| N | 2.3009422 | 15.8445029 | 9.7730212 |
| N | 5.7452462 | 20.2016482 | 10.6154054 |
| N | 3.8079005 | 20.5854311 | 9.7843691 |
| N | 2.3908181 | 18.2454417 | 11.3261450 |
| C | 2.7045053 | 15.1671540 | 10.8483794 |
| C | 1.9314096 | 14.1534492 | 11.6084702 |
| C | 1.1168840 | 14.6020737 | 12.6493186 |
| H | 1.0726489 | 15.6611844 | 12.8692805 |
| C | 0.3857939 | 13.6937505 | 13.4040948 |
| H | -0.2407868 | 14.0495104 | 14.2123278 |
| C | 0.4638967 | 12.3324739 | 13.1256153 |
| H | -0.1042561 | 11.6241299 | 13.7154250 |
| C | 1.2731905 | 11.8833118 | 12.0868537 |
| H | 1.3340755 | 10.8254724 | 11.8637614 |
| C | 2.0042078 | 12.7906533 | 11.3294356 |
| H | 2.6210952 | 12.4428874 | 10.5113600 |
| C | 4.9329500 | 15.0877681 | 12.0809527 |
| C | 4.4128475 | 14.8833539 | 13.5104733 |
| H | 3.9753111 | 15.8089843 | 13.8788142 |
| H | 5.2428557 | 14.6081702 | 14.1644624 |
| H | 3.6654466 | 14.0946743 | 13.5696678 |
| C | 6.0893199 | 16.0904161 | 12.1118973 |
| H | 6.4917364 | 16.2313787 | 11.1087045 |
| H | 6.8848178 | 15.7220030 | 12.7618290 |
| H | 5.7403168 | 17.0525558 | 12.4840480 |
| C | 5.4419996 | 13.7576012 | 11.5016491 |
| H | 4.6638983 | 12.9953924 | 11.5127110 |
| H | 6.2826556 | 13.3869312 | 12.0921527 |
| H | 5.7785367 | 13.8995325 | 10.4735446 |
| C | 1.0211514 | 15.8161193 | 9.0419312 |
| C | -0.0613321 | 16.5906043 | 9.8107515 |
| H | 0.2617717 | 17.6080342 | 10.0191588 |
| H | -0.2775617 | 16.1056811 | 10.7632397 |
| H | -0.9844322 | 16.6204341 | 9.2273025 |
| C | 0.5390646 | 14.3794471 | 8.7798760 |

| | | | |
|---|---|---|---|
| H | 0.1890249 | 13.8877293 | 9.6846505 |
| H | 1.3382336 | 13.7779588 | 8.3421680 |
| H | -0.2928552 | 14.4063122 | 8.0735532 |
| C | 1.2781310 | 16.4806465 | 7.6824663 |
| H | 0.3428569 | 16.5521773 | 7.1245281 |
| H | 1.9892002 | 15.8949272 | 7.0991081 |
| H | 1.6934204 | 17.4782302 | 7.7973424 |
| C | 4.7741025 | 21.1160773 | 10.5383418 |
| C | 4.7287716 | 22.4177584 | 11.2476829 |
| C | 5.1270886 | 23.6134262 | 10.6539029 |
| H | 5.5059398 | 23.6105927 | 9.6403712 |
| C | 5.0559726 | 24.8026313 | 11.3697958 |
| H | 5.3697919 | 25.7285185 | 10.9044420 |
| C | 4.5863469 | 24.8035933 | 12.6796664 |
| H | 4.5304075 | 25.7318510 | 13.2343097 |
| C | 4.1899902 | 23.6098274 | 13.2747733 |
| H | 3.8208601 | 23.6014121 | 14.2926199 |
| C | 4.2622792 | 22.4206269 | 12.5617600 |
| H | 3.9491795 | 21.4865344 | 13.0063433 |
| C | 7.0677072 | 20.2516760 | 11.2680520 |
| C | 7.8128988 | 21.5579438 | 10.9489163 |
| H | 8.8419685 | 21.4818376 | 11.3056139 |
| H | 7.3583084 | 22.4197518 | 11.4320194 |
| H | 7.8375473 | 21.7331631 | 9.8713527 |
| C | 7.8870101 | 19.0846544 | 10.7016972 |
| H | 8.0275034 | 19.1950486 | 9.6258035 |
| H | 7.3892421 | 18.1342795 | 10.8736020 |
| H | 8.8663831 | 19.0589571 | 11.1823656 |
| C | 6.9200830 | 20.0847503 | 12.7872623 |
| H | 6.3436811 | 19.1901211 | 13.0107115 |
| H | 6.4027969 | 20.9380721 | 13.2255096 |
| H | 7.9044896 | 20.0080093 | 13.2545199 |
| C | 2.5482470 | 21.1766144 | 9.2910514 |
| C | 1.6515984 | 21.6433164 | 10.4487352 |
| H | 1.4948917 | 20.8266637 | 11.1534200 |
| H | 0.6810717 | 21.9517727 | 10.0541843 |
| H | 2.0797770 | 22.4913822 | 10.9797440 |
| C | 1.8148440 | 20.0800974 | 8.5137447 |
| H | 2.4360324 | 19.6937381 | 7.7047735 |
| H | 0.8945883 | 20.4833623 | 8.0879898 |
| H | 1.5591091 | 19.2582860 | 9.1775826 |
| C | 2.8455390 | 22.3441423 | 8.3357063 |
| H | 3.3022065 | 23.1827297 | 8.8583150 |
| H | 1.9178420 | 22.6996247 | 7.8822183 |

| | | | |
|---|---|---|---|
| H | 3.5159025 | 22.0192435 | 7.5376892 |
| C | 4.7610852 | 16.9213033 | 6.2148939 |
| H | 3.8295624 | 17.0163381 | 5.6754609 |
| C | 5.0466458 | 15.7422023 | 6.9419374 |
| H | 4.3195064 | 14.9433340 | 6.9782371 |
| C | 6.2315563 | 15.6435210 | 7.7101978 |
| H | 6.4082369 | 14.7651541 | 8.3144242 |
| C | 7.1391821 | 16.7250395 | 7.7540109 |
| H | 8.0172767 | 16.6751070 | 8.3826923 |
| C | 6.8706805 | 17.8891342 | 6.9984265 |
| H | 7.5309083 | 18.7417301 | 7.0738964 |
| C | 5.6841848 | 17.9897032 | 6.2368273 |
| H | 5.4496092 | 18.9148031 | 5.7297503 |

Compound **4**

111

E= -4037,07239

| | | | |
|---|---|---|---|
| Fe | 5.1106086 | 17.4673073 | 8.2439896 |
| Si | 3.9901390 | 16.5198715 | 9.8357445 |
| Si | 4.6997841 | 18.9203676 | 9.8001544 |
| Si | 2.5728612 | 19.2303088 | 14.3332185 |
| N | 3.2246963 | 17.9823058 | 12.1612506 |
| N | 3.2674098 | 19.1229699 | 12.7275423 |
| C | 3.9598864 | 19.7387642 | 15.5006897 |
| H | 4.4329450 | 20.6661462 | 15.1684231 |
| H | 4.7342913 | 18.9684287 | 15.5385175 |
| H | 3.5900705 | 19.8964944 | 16.5175012 |
| C | 1.2733498 | 20.5916252 | 14.2980543 |
| H | 0.8646593 | 20.7741335 | 15.2956925 |
| H | 0.4445681 | 20.3228389 | 13.6386185 |
| H | 1.6943911 | 21.5305354 | 13.9311179 |
| C | 1.7975125 | 17.6202295 | 14.9231110 |
| H | 1.3788110 | 17.7277473 | 15.9272260 |
| H | 2.5420291 | 16.8216305 | 14.9485505 |
| H | 0.9934645 | 17.3119349 | 14.2509936 |
| N | 4.1828141 | 15.0324284 | 10.9986751 |
| N | 2.3574840 | 15.5709536 | 10.0073692 |
| N | 5.7662640 | 20.0780512 | 10.8244506 |
| N | 3.9369651 | 20.6379518 | 9.8618650 |
| N | 3.7835323 | 17.8993813 | 10.9668959 |
| C | 2.8836619 | 14.7590524 | 10.9199986 |
| C | 2.1428921 | 13.7754827 | 11.7480541 |
| C | 1.6184838 | 14.1960045 | 12.9686719 |

| | | | |
|---|---|---|---|
| H | 1.7622666 | 15.2207291 | 13.2837441 |
| C | 0.9186683 | 13.2996054 | 13.7681083 |
| H | 0.5134365 | 13.6314011 | 14.7157304 |
| C | 0.7414165 | 11.9844183 | 13.3513340 |
| H | 0.1964694 | 11.2864601 | 13.9743938 |
| C | 1.2659924 | 11.5658711 | 12.1313107 |
| H | 1.1298002 | 10.5427323 | 11.8042613 |
| C | 1.9652641 | 12.4584603 | 11.3302758 |
| H | 2.3743295 | 12.1366850 | 10.3812909 |
| C | 5.2397828 | 14.5439843 | 11.8988606 |
| C | 5.1824393 | 15.3224572 | 13.2242947 |
| H | 5.2400431 | 16.3947657 | 13.0445488 |
| H | 6.0092748 | 15.0266591 | 13.8738678 |
| H | 4.2460782 | 15.1221855 | 13.7460898 |
| C | 6.5710200 | 14.8231083 | 11.1859897 |
| H | 6.6429104 | 14.2347720 | 10.2694320 |
| H | 7.4065331 | 14.5600242 | 11.8369642 |
| H | 6.6567185 | 15.8722614 | 10.9088661 |
| C | 5.1456102 | 13.0369112 | 12.1779801 |
| H | 4.2962611 | 12.7831454 | 12.8085024 |
| H | 6.0519739 | 12.7151623 | 12.6944980 |
| H | 5.0670980 | 12.4737820 | 11.2462692 |
| C | 0.9678247 | 15.8260029 | 9.5965054 |
| C | 0.3117420 | 16.7958477 | 10.5944918 |
| H | 0.9027313 | 17.7045529 | 10.6966464 |
| H | 0.2361935 | 16.3362419 | 11.5807070 |
| H | -0.6934338 | 17.0625910 | 10.2605583 |
| C | 0.1254131 | 14.5468002 | 9.4882230 |
| H | -0.0930363 | 14.1092750 | 10.4599916 |
| H | 0.6330890 | 13.7986407 | 8.8768266 |
| H | -0.8256436 | 14.7887369 | 9.0099584 |
| C | 1.0467381 | 16.4737066 | 8.2056771 |
| H | 0.0510827 | 16.7761881 | 7.8764312 |
| H | 1.4550785 | 15.7669048 | 7.4807737 |
| H | 1.6985357 | 17.3462336 | 8.2108827 |
| C | 4.9334296 | 21.0935736 | 10.6161084 |
| C | 5.0607194 | 22.4566933 | 11.1893922 |
| C | 5.7189369 | 23.4796136 | 10.5126671 |
| H | 6.1350788 | 23.2946629 | 9.5306334 |
| C | 5.8368639 | 24.7358241 | 11.0984626 |
| H | 6.3503736 | 25.5289314 | 10.5697100 |
| C | 5.2967923 | 24.9716278 | 12.3581035 |
| H | 5.3903547 | 25.9498345 | 12.8128297 |
| C | 4.6363778 | 23.9483970 | 13.0334749 |

| | | | |
|---|---|---|---|
| H | 4.2146992 | 24.1275305 | 14.0146009 |
| C | 4.5188982 | 22.6938068 | 12.4524257 |
| H | 4.0164115 | 21.8828726 | 12.9625309 |
| C | 7.1062386 | 20.0205435 | 11.4267453 |
| C | 7.1547187 | 20.6807398 | 12.8126097 |
| H | 8.1099287 | 20.4495321 | 13.2884500 |
| H | 6.3511488 | 20.2960796 | 13.4416111 |
| H | 7.0642796 | 21.7636647 | 12.7573498 |
| C | 8.1255674 | 20.6775605 | 10.4813949 |
| H | 7.9137737 | 21.7404950 | 10.3596012 |
| H | 8.0909166 | 20.2002292 | 9.5002774 |
| H | 9.1376108 | 20.5779054 | 10.8801028 |
| C | 7.4422732 | 18.5319491 | 11.5791857 |
| H | 7.3773512 | 18.0227536 | 10.6165226 |
| H | 6.7421232 | 18.0526549 | 12.2644648 |
| H | 8.4514879 | 18.4137336 | 11.9769252 |
| C | 2.6984110 | 21.2800577 | 9.3938801 |
| C | 1.8476411 | 21.7866714 | 10.5691306 |
| H | 1.7317311 | 20.9970407 | 11.3113990 |
| H | 0.8606654 | 22.0851463 | 10.2090491 |
| H | 2.3011414 | 22.6498956 | 11.0530151 |
| C | 1.9075474 | 20.1994966 | 8.6466826 |
| H | 2.5009158 | 19.7596470 | 7.8442969 |
| H | 0.9989807 | 20.6297808 | 8.2225979 |
| H | 1.6203606 | 19.3991893 | 9.3279880 |
| C | 3.0220571 | 22.4279555 | 8.4252034 |
| H | 3.5611825 | 23.2305187 | 8.9271485 |
| H | 2.0992404 | 22.8465979 | 8.0178149 |
| H | 3.6313995 | 22.0637477 | 7.5957421 |
| C | 4.5011048 | 17.2130532 | 6.2501723 |
| H | 3.5095496 | 17.1727665 | 5.8214790 |
| C | 5.1284095 | 16.0304352 | 6.7106369 |
| H | 4.5944453 | 15.0913357 | 6.6677550 |
| C | 6.4009729 | 16.0807318 | 7.3287739 |
| H | 6.8359338 | 15.1793227 | 7.7372829 |
| C | 7.0631964 | 17.3172564 | 7.4835595 |
| H | 8.0198365 | 17.3674525 | 7.9848614 |
| C | 6.4493049 | 18.4959447 | 6.9950098 |
| H | 6.9214868 | 19.4540974 | 7.1636515 |
| C | 5.1728687 | 18.4467611 | 6.3839506 |
| H | 4.6899862 | 19.3636396 | 6.0750775 |

Compound TS**2**

111

E= -4037,03353

| | | | |
|---|---|---|---|
| Fe | 5.1223520 | 17.5258050 | 9.3552989 |
| Si | 3.7872228 | 17.0733837 | 11.1232870 |
| Si | 4.5041266 | 19.4272478 | 10.1604310 |
| Si | 3.2753116 | 17.5431440 | 14.9234157 |
| N | 3.4327787 | 18.9948663 | 12.7355627 |
| N | 3.5422263 | 17.7800403 | 13.1887049 |
| C | 4.8377017 | 16.9403485 | 15.7770447 |
| H | 5.6705908 | 17.6164994 | 15.5686054 |
| H | 5.1364126 | 15.9379297 | 15.4713294 |
| H | 4.6826615 | 16.9239325 | 16.8598310 |
| C | 2.7458552 | 19.1623060 | 15.7086932 |
| H | 2.5333112 | 19.0206668 | 16.7721250 |
| H | 1.8489534 | 19.5566616 | 15.2274610 |
| H | 3.5290981 | 19.9167619 | 15.6127366 |
| C | 1.9014702 | 16.2702514 | 15.0819876 |
| H | 1.7258021 | 15.9916659 | 16.1243906 |
| H | 2.1476398 | 15.3658509 | 14.5253120 |
| H | 0.9702487 | 16.6696400 | 14.6731009 |
| N | 3.8269730 | 15.2119751 | 11.7985972 |
| N | 2.1026721 | 16.2160754 | 10.9920860 |
| N | 5.4905337 | 20.8473793 | 10.9504658 |
| N | 3.9413275 | 21.1016247 | 9.4852000 |
| N | 3.3797430 | 18.8949947 | 11.4352298 |
| C | 2.5894707 | 15.0281253 | 11.3779424 |
| C | 1.8931214 | 13.7183207 | 11.2558249 |
| C | 1.1689450 | 13.1819882 | 12.3183555 |
| H | 1.1048641 | 13.7269861 | 13.2507403 |
| C | 0.5144081 | 11.9647514 | 12.1761095 |
| H | -0.0497442 | 11.5584807 | 13.0063039 |
| C | 0.5792376 | 11.2726283 | 10.9709402 |
| H | 0.0674007 | 10.3248879 | 10.8604074 |
| C | 1.3037092 | 11.8028656 | 9.9079407 |
| H | 1.3600666 | 11.2688881 | 8.9676129 |
| C | 1.9573882 | 13.0205138 | 10.0498162 |
| H | 2.5219843 | 13.4347769 | 9.2245928 |
| C | 4.8306212 | 14.2636583 | 12.3087386 |
| C | 4.3568863 | 13.5793264 | 13.6025048 |
| H | 4.0724971 | 14.3163044 | 14.3509695 |
| H | 5.1655568 | 12.9716026 | 14.0148568 |
| H | 3.5070905 | 12.9234983 | 13.4212552 |
| C | 6.0827110 | 15.0964901 | 12.6235355 |
| H | 6.4427708 | 15.6098205 | 11.7344427 |
| H | 6.8734519 | 14.4519244 | 13.0121047 |
| H | 5.8610850 | 15.8606360 | 13.3667142 |

| | | | |
|---|---|---|---|
| C | 5.1871095 | 13.1774148 | 11.2786672 |
| H | 4.3509394 | 12.5016022 | 11.1060493 |
| H | 6.0278204 | 12.5829544 | 11.6433763 |
| H | 5.4720420 | 13.6209587 | 10.3271839 |
| C | 0.8291922 | 16.5538662 | 10.3212205 |
| C | 0.4733852 | 17.9969221 | 10.7081366 |
| H | 1.2517246 | 18.6914352 | 10.4089761 |
| H | 0.3528996 | 18.0837692 | 11.7894775 |
| H | -0.4617054 | 18.2895530 | 10.2267975 |
| C | -0.3473799 | 15.6638958 | 10.7533970 |
| H | -0.4251760 | 15.6217492 | 11.8410281 |
| H | -0.2750208 | 14.6484002 | 10.3726308 |
| H | -1.2707592 | 16.0964514 | 10.3637563 |
| C | 1.0221085 | 16.4632599 | 8.7980274 |
| H | 0.1218580 | 16.7940790 | 8.2743808 |
| H | 1.2320537 | 15.4344466 | 8.4994021 |
| H | 1.8645213 | 17.0845790 | 8.4939332 |
| C | 4.8592261 | 21.7485613 | 10.2089183 |
| C | 5.1397094 | 23.2056285 | 10.1647360 |
| C | 6.0240224 | 23.7200483 | 9.2184643 |
| H | 6.4972995 | 23.0527594 | 8.5096636 |
| C | 6.2878017 | 25.0834259 | 9.1811100 |
| H | 6.9767266 | 25.4777564 | 8.4447444 |
| C | 5.6660617 | 25.9395416 | 10.0848819 |
| H | 5.8708349 | 27.0022662 | 10.0540554 |
| C | 4.7813616 | 25.4271501 | 11.0287342 |
| H | 4.2970458 | 26.0893466 | 11.7352900 |
| C | 4.5198937 | 24.0633621 | 11.0713483 |
| H | 3.8433547 | 23.6588896 | 11.8131211 |
| C | 6.7756031 | 20.8945289 | 11.6709748 |
| C | 6.9405928 | 22.1713959 | 12.5062051 |
| H | 7.8238189 | 22.0691168 | 13.1397108 |
| H | 6.0736172 | 22.3227053 | 13.1514375 |
| H | 7.0732775 | 23.0590222 | 11.8903671 |
| C | 7.9263848 | 20.7649628 | 10.6588076 |
| H | 7.9380332 | 21.6154522 | 9.9744552 |
| H | 7.8098468 | 19.8488909 | 10.0782986 |
| H | 8.8881042 | 20.7315116 | 11.1756344 |
| C | 6.7807421 | 19.6809175 | 12.6125371 |
| H | 6.6301389 | 18.7569947 | 12.0504849 |
| H | 5.9792442 | 19.7655710 | 13.3470013 |
| H | 7.7357846 | 19.6204906 | 13.1376982 |
| C | 2.7471441 | 21.5886860 | 8.7764166 |
| C | 1.6627408 | 21.9496680 | 9.8064501 |

| | | | |
|---|---|---|---|
| H | 1.4672471 | 21.1038795 | 10.4661917 |
| H | 0.7328252 | 22.2258363 | 9.3043170 |
| H | 1.9814364 | 22.7950838 | 10.4183693 |
| C | 2.2597140 | 20.4290230 | 7.8964608 |
| H | 3.0117479 | 20.1711471 | 7.1495368 |
| H | 1.3393945 | 20.7127831 | 7.3831429 |
| H | 2.0637121 | 19.5352579 | 8.4873491 |
| C | 3.0391237 | 22.7951692 | 7.8716462 |
| H | 3.2755264 | 23.6924672 | 8.4397212 |
| H | 2.1578359 | 23.0079303 | 7.2632352 |
| H | 3.8734871 | 22.5808979 | 7.2014356 |
| C | 4.6248718 | 16.6472620 | 7.5254912 |
| H | 3.6604276 | 16.4830349 | 7.0669172 |
| C | 5.1937073 | 15.6686419 | 8.3741041 |
| H | 4.6410083 | 14.7669815 | 8.5971854 |
| C | 6.4375087 | 15.9025329 | 9.0029284 |
| H | 6.8395747 | 15.1709732 | 9.6871460 |
| C | 7.1227696 | 17.1204465 | 8.8054938 |
| H | 8.0551165 | 17.3123685 | 9.3184152 |
| C | 6.5749062 | 18.0818118 | 7.9264130 |
| H | 7.0644573 | 19.0375274 | 7.7989649 |
| C | 5.3341542 | 17.8450724 | 7.2909839 |
| H | 4.8888873 | 18.6223596 | 6.6859587 |

Compound Int**2**

111

E= -4037,03454

| | | | |
|---|---|---|---|
| Fe | 5.1437542 | 17.5423524 | 9.4868143 |
| Si | 3.8090572 | 17.0877811 | 11.3008491 |
| Si | 4.5042304 | 19.4700644 | 10.2050655 |
| Si | 3.3550789 | 17.4069500 | 14.8893492 |
| N | 3.5407877 | 19.0165274 | 12.7993458 |
| N | 3.6502196 | 17.7498117 | 13.1738540 |
| C | 4.8825981 | 16.7582324 | 15.7708335 |
| H | 5.7426109 | 17.3996722 | 15.5644889 |
| H | 5.1501726 | 15.7390443 | 15.4964839 |
| H | 4.7060789 | 16.7738684 | 16.8503179 |
| C | 2.8330767 | 19.0008387 | 15.7277721 |
| H | 2.5902629 | 18.8118826 | 16.7773441 |
| H | 1.9561158 | 19.4331794 | 15.2430998 |
| H | 3.6307995 | 19.7446698 | 15.6885481 |
| C | 1.9529542 | 16.1596796 | 14.9640157 |
| H | 1.7717676 | 15.8180109 | 15.9864869 |
| H | 2.1768757 | 15.2908110 | 14.3469025 |
| H | 1.0322766 | 16.6092771 | 14.5850904 |

| | | | |
|---|---|---|---|
| N | 3.8187419 | 15.1704415 | 11.9015690 |
| N | 2.1260996 | 16.2374831 | 11.0962202 |
| N | 5.4846561 | 20.9287138 | 10.9351463 |
| N | 3.9140518 | 21.1174298 | 9.4826275 |
| N | 3.4025001 | 18.9622733 | 11.5104766 |
| C | 2.5926739 | 15.0264462 | 11.4351174 |
| C | 1.8865981 | 13.7333556 | 11.2163177 |
| C | 1.1680179 | 13.1164903 | 12.2379477 |
| H | 1.1096723 | 13.5862926 | 13.2106467 |
| C | 0.5075897 | 11.9163489 | 12.0049622 |
| H | -0.0530422 | 11.4483637 | 12.8044391 |
| C | 0.5619615 | 11.3206104 | 10.7491376 |
| H | 0.0463943 | 10.3858730 | 10.5681934 |
| C | 1.2809325 | 11.9310534 | 9.7261267 |
| H | 1.3297645 | 11.4724254 | 8.7464799 |
| C | 1.9386691 | 13.1321144 | 9.9585574 |
| H | 2.4985142 | 13.6077926 | 9.1641777 |
| C | 4.8004747 | 14.1673217 | 12.3520110 |
| C | 4.3239764 | 13.4412136 | 13.6227529 |
| H | 4.0715858 | 14.1499069 | 14.4088259 |
| H | 5.1189794 | 12.7912152 | 13.9952388 |
| H | 3.4525040 | 12.8197724 | 13.4243423 |
| C | 6.0816938 | 14.9473080 | 12.6841516 |
| H | 6.4443982 | 15.4898014 | 11.8144399 |
| H | 6.8587690 | 14.2637616 | 13.0312959 |
| H | 5.8962304 | 15.6831350 | 13.4636786 |
| C | 5.1161451 | 13.1102451 | 11.2786932 |
| H | 4.2636937 | 12.4591984 | 11.0933782 |
| H | 5.9465821 | 12.4845006 | 11.6129343 |
| H | 5.4017842 | 13.5770117 | 10.3391891 |
| C | 0.8490690 | 16.6189736 | 10.4539594 |
| C | 0.4992648 | 18.0401989 | 10.9211066 |
| H | 1.2737313 | 18.7512229 | 10.6526595 |
| H | 0.3887764 | 18.0679347 | 12.0068139 |
| H | -0.4412566 | 18.3575181 | 10.4670078 |
| C | -0.3317386 | 15.7152445 | 10.8466373 |
| H | -0.4007670 | 15.6131031 | 11.9308725 |
| H | -0.2730069 | 14.7223562 | 10.4091611 |
| H | -1.2543483 | 16.1770086 | 10.4902969 |
| C | 1.0308157 | 16.6081323 | 8.9268732 |
| H | 0.1392114 | 16.9958771 | 8.4281597 |
| H | 1.2058751 | 15.5917328 | 8.5699360 |
| H | 1.8921877 | 17.2165868 | 8.6522319 |
| C | 4.8379112 | 21.7968942 | 10.1690852 |

| | | | |
|---|---|---|---|
| C | 5.1114123 | 23.2523315 | 10.0627523 |
| C | 5.9923373 | 23.7285564 | 9.0933561 |
| H | 6.4656554 | 23.0331039 | 8.4123042 |
| C | 6.2523338 | 25.0896529 | 8.9970841 |
| H | 6.9384447 | 25.4538101 | 8.2428321 |
| C | 5.6305673 | 25.9820514 | 9.8650021 |
| H | 5.8324314 | 27.0429556 | 9.7883707 |
| C | 4.7496650 | 25.5080458 | 10.8320301 |
| H | 4.2656160 | 26.1983703 | 11.5112355 |
| C | 4.4918400 | 24.1466646 | 10.9333173 |
| H | 3.8191691 | 23.7730584 | 11.6944149 |
| C | 6.7710446 | 21.0155563 | 11.6501371 |
| C | 6.9226556 | 22.3226562 | 12.4398676 |
| H | 7.8069083 | 22.2518956 | 13.0761264 |
| H | 6.0544959 | 22.4872746 | 13.0800346 |
| H | 7.0460157 | 23.1894094 | 11.7932403 |
| C | 7.9220716 | 20.8641130 | 10.6416542 |
| H | 7.9206716 | 21.6870430 | 9.9245720 |
| H | 7.8193449 | 19.9242522 | 10.0983050 |
| H | 8.8844089 | 20.8655001 | 11.1583027 |
| C | 6.7926485 | 19.8357288 | 12.6333555 |
| H | 6.6478367 | 18.8914465 | 12.1041563 |
| H | 5.9948006 | 19.9387963 | 13.3691200 |
| H | 7.7516342 | 19.8021999 | 13.1536746 |
| C | 2.7197620 | 21.5761666 | 8.7553299 |
| C | 1.6495338 | 22.0223295 | 9.7665507 |
| H | 1.4454523 | 21.2241614 | 10.4810072 |
| H | 0.7202121 | 22.2786285 | 9.2530162 |
| H | 1.9840256 | 22.9015359 | 10.3189658 |
| C | 2.2022689 | 20.3693731 | 7.9611894 |
| H | 2.9466930 | 20.0342790 | 7.2380657 |
| H | 1.2907273 | 20.6397220 | 7.4257213 |
| H | 1.9781525 | 19.5300483 | 8.6172889 |
| C | 3.0236363 | 22.7142755 | 7.7692895 |
| H | 3.2824832 | 23.6423552 | 8.2744688 |
| H | 2.1405702 | 22.9035101 | 7.1557720 |
| H | 3.8470259 | 22.4396427 | 7.1076668 |
| C | 4.6278758 | 16.6717215 | 7.6612236 |
| H | 3.6610385 | 16.5317782 | 7.1998344 |
| C | 5.1729283 | 15.6803496 | 8.5089858 |
| H | 4.6002166 | 14.7914194 | 8.7303852 |
| C | 6.4235376 | 15.8821318 | 9.1354932 |
| H | 6.8073180 | 15.1383790 | 9.8162173 |
| C | 7.1400354 | 17.0823873 | 8.9423974 |

| | | | |
|---|---|---|---|
| H | 8.0790245 | 17.2476411 | 9.4523992 |
| C | 6.6175445 | 18.0557550 | 8.0619716 |
| H | 7.1320943 | 18.9974247 | 7.9292586 |
| C | 5.3715664 | 17.8492401 | 7.4264110 |
| H | 4.9472123 | 18.6389346 | 6.8223070 |

Compound TS**3**

111

E= -4037,02559

| | | | |
|---|---|---|---|
| Fe | 4.9930235 | 17.6909309 | 9.6815038 |
| Si | 3.7546262 | 17.0574926 | 11.4564102 |
| Si | 4.3711768 | 19.5639999 | 10.5255827 |
| Si | 3.2121701 | 17.1365752 | 14.9027955 |
| N | 3.4368720 | 19.0206972 | 13.0931139 |
| N | 3.5065662 | 17.6908752 | 13.2338157 |
| C | 4.7800315 | 16.5862501 | 15.7805115 |
| H | 5.5554760 | 17.3486442 | 15.6729679 |
| H | 5.1853250 | 15.6426320 | 15.4194496 |
| H | 4.5725637 | 16.4700233 | 16.8484004 |
| C | 2.4931108 | 18.5727674 | 15.8693316 |
| H | 2.2221476 | 18.2416524 | 16.8763430 |
| H | 1.5980553 | 18.9656886 | 15.3836611 |
| H | 3.2073204 | 19.3924331 | 15.9540175 |
| C | 1.9366540 | 15.7602895 | 14.8541709 |
| H | 1.7182009 | 15.4082664 | 15.8660708 |
| H | 2.2772501 | 14.9128348 | 14.2650242 |
| H | 1.0068116 | 16.1269625 | 14.4141068 |
| N | 3.8235302 | 15.1655727 | 11.9512211 |
| N | 2.0787068 | 16.1852759 | 11.2106059 |
| N | 5.4821513 | 21.0335090 | 11.0216157 |
| N | 3.8191069 | 21.1666216 | 9.6715737 |
| N | 3.2953729 | 19.3073729 | 11.8631711 |
| C | 2.5952300 | 14.9852112 | 11.4712454 |
| C | 1.9569305 | 13.6806514 | 11.1455126 |
| C | 1.3234028 | 12.9091149 | 12.1164997 |
| H | 1.2817925 | 13.2601338 | 13.1385446 |
| C | 0.7197466 | 11.7057928 | 11.7708326 |
| H | 0.2235441 | 11.1167810 | 12.5319115 |
| C | 0.7474580 | 11.2622129 | 10.4530506 |
| H | 0.2772242 | 10.3244255 | 10.1853097 |
| C | 1.3791018 | 12.0298654 | 9.4791015 |
| H | 1.4045936 | 11.6913464 | 8.4509462 |
| C | 1.9772656 | 13.2346302 | 9.8232265 |
| H | 2.4655884 | 13.8354119 | 9.0673751 |

| | | | |
|---|---|---|---|
| C | 4.8741051 | 14.1779177 | 12.2757616 |
| C | 4.4935616 | 13.3417601 | 13.5104525 |
| H | 4.2738853 | 13.9763626 | 14.3663001 |
| H | 5.3252810 | 12.6870999 | 13.7799924 |
| H | 3.6271029 | 12.7125206 | 13.3137343 |
| C | 6.1375421 | 14.9878177 | 12.6023284 |
| H | 6.4348958 | 15.6059666 | 11.7585417 |
| H | 6.9566456 | 14.3146523 | 12.8616458 |
| H | 5.9638804 | 15.6559841 | 13.4437288 |
| C | 5.1757846 | 13.2126077 | 11.1157952 |
| H | 4.3480507 | 12.5293134 | 10.9351116 |
| H | 6.0548304 | 12.6121983 | 11.3595147 |
| H | 5.3804298 | 13.7534441 | 10.1959377 |
| C | 0.7593281 | 16.5903072 | 10.6890026 |
| C | 0.3982406 | 17.9187003 | 11.3721884 |
| H | 1.1755463 | 18.6646442 | 11.2253091 |
| H | 0.2808297 | 17.7722711 | 12.4477173 |
| H | -0.5420959 | 18.3019141 | 10.9712863 |
| C | -0.3643343 | 15.5906740 | 11.0027640 |
| H | -0.3834340 | 15.3439393 | 12.0654544 |
| H | -0.2825164 | 14.6668306 | 10.4356248 |
| H | -1.3189669 | 16.0534614 | 10.7458437 |
| C | 0.8704916 | 16.7946023 | 9.1696243 |
| H | -0.0570684 | 17.2077109 | 8.7665249 |
| H | 1.0666138 | 15.8431252 | 8.6717235 |
| H | 1.6934758 | 17.4712851 | 8.9460174 |
| C | 4.8447340 | 21.8466245 | 10.1926086 |
| C | 5.2370013 | 23.2446572 | 9.8719585 |
| C | 6.1692943 | 23.4906494 | 8.8648772 |
| H | 6.5757124 | 22.6623955 | 8.2989241 |
| C | 6.5676668 | 24.7917502 | 8.5837981 |
| H | 7.2913164 | 24.9744660 | 7.7994414 |
| C | 6.0381079 | 25.8550792 | 9.3079012 |
| H | 6.3499984 | 26.8687293 | 9.0901308 |
| C | 5.1076604 | 25.6123355 | 10.3134941 |
| H | 4.6950672 | 26.4359451 | 10.8824181 |
| C | 4.7084256 | 24.3120448 | 10.5956116 |
| H | 3.9965103 | 24.1193939 | 11.3875900 |
| C | 6.7426318 | 21.1488028 | 11.7692671 |
| C | 6.9155666 | 22.5210056 | 12.4350220 |
| H | 7.7753427 | 22.4860281 | 13.1069299 |
| H | 6.0328365 | 22.7762458 | 13.0236751 |
| H | 7.0909773 | 23.3145388 | 11.7109322 |
| C | 7.9271261 | 20.8609274 | 10.8318138 |

| | | | |
|---|---|---|---|
| H | 7.9861689 | 21.6085981 | 10.0392478 |
| H | 7.8110387 | 19.8764062 | 10.3790303 |
| H | 8.8671812 | 20.8810246 | 11.3878630 |
| C | 6.6841998 | 20.0689849 | 12.8620258 |
| H | 6.5444775 | 19.0814161 | 12.4168892 |
| H | 5.8519633 | 20.2551391 | 13.5419103 |
| H | 7.6139949 | 20.0652466 | 13.4338908 |
| C | 2.7428263 | 21.5897957 | 8.7628750 |
| C | 1.8577449 | 22.6565489 | 9.4288327 |
| H | 1.5105747 | 22.3022310 | 10.4007038 |
| H | 0.9865700 | 22.8651025 | 8.8037091 |
| H | 2.3983013 | 23.5913198 | 9.5700400 |
| C | 1.8892368 | 20.3453857 | 8.4883718 |
| H | 2.4900416 | 19.5477468 | 8.0527270 |
| H | 1.0795535 | 20.5931177 | 7.8000295 |
| H | 1.4495013 | 19.9673452 | 9.4108109 |
| C | 3.2984168 | 22.1164354 | 7.4295644 |
| H | 3.8536938 | 23.0434981 | 7.5610154 |
| H | 2.4779149 | 22.3125761 | 6.7361002 |
| H | 3.9613980 | 21.3779333 | 6.9750999 |
| C | 4.4348927 | 16.9658330 | 7.8034521 |
| H | 3.4556106 | 16.8265296 | 7.3683362 |
| C | 5.0569820 | 15.9222215 | 8.5278919 |
| H | 4.5291193 | 14.9921529 | 8.6817065 |
| C | 6.3243576 | 16.1128746 | 9.1207746 |
| H | 6.7697575 | 15.3204758 | 9.7023562 |
| C | 6.9819070 | 17.3565937 | 9.0210586 |
| H | 7.9364345 | 17.5115885 | 9.5046800 |
| C | 6.3827173 | 18.3871957 | 8.2625296 |
| H | 6.8518904 | 19.3598379 | 8.2075213 |
| C | 5.1203451 | 18.1903044 | 7.6550595 |
| H | 4.6469781 | 19.0128658 | 7.1375566 |

Compound **5**

109

E= -3927,6511

| | | | |
|---|---|---|---|
| Fe | 12.6087723 | 7.6362958 | 1.6459379 |
| Si | 11.6348270 | 7.0049179 | 3.4865382 |
| Si | 13.6951047 | 8.2621142 | 3.4220609 |
| Si | 12.7054092 | 7.7193873 | 6.4682643 |
| N | 15.5362652 | 8.1222526 | 3.9087926 |
| N | 9.8222077 | 7.1564502 | 4.0584340 |
| N | 10.9291894 | 5.3196372 | 4.0328158 |
| N | 14.4095653 | 9.9520347 | 3.9538068 |

| | | | |
|---|---|---|---|
| N | 12.7020522 | 7.6323633 | 4.7532898 |
| C | 8.4617608 | 5.0641414 | 4.4156757 |
| C | 7.7210307 | 4.5618344 | 3.3475912 |
| H | 8.0441063 | 4.7558672 | 2.3333645 |
| C | 9.7121956 | 5.8342597 | 4.1796118 |
| C | 8.0392669 | 4.8172697 | 5.7216203 |
| H | 8.6196786 | 5.1997638 | 6.5509249 |
| C | 8.7819937 | 8.1978760 | 3.9865755 |
| C | 13.9894304 | 11.3503984 | 3.7328298 |
| C | 15.6365913 | 9.4391896 | 4.0566213 |
| C | 11.3757695 | 3.9230176 | 3.8923997 |
| C | 6.5697845 | 3.8190714 | 3.5828719 |
| H | 6.0001705 | 3.4329180 | 2.7470074 |
| C | 6.1522636 | 3.5733450 | 4.8864561 |
| H | 5.2565789 | 2.9931957 | 5.0692412 |
| C | 6.8892717 | 4.0748941 | 5.9550831 |
| H | 6.5698719 | 3.8858302 | 6.9722032 |
| C | 12.8976153 | 3.9537953 | 4.0620186 |
| H | 13.3407240 | 4.6092392 | 3.3142909 |
| H | 13.3139561 | 2.9523319 | 3.9418878 |
| H | 13.1694598 | 4.3293390 | 5.0491790 |
| C | 9.5240700 | 9.5296988 | 3.8467075 |
| H | 10.1692388 | 9.7007291 | 4.7069737 |
| H | 8.8116537 | 10.3533280 | 3.7780796 |
| H | 10.1495871 | 9.5233041 | 2.9539787 |
| C | 16.5804406 | 7.0924591 | 3.7508785 |
| C | 15.8533975 | 5.7452045 | 3.7712924 |
| H | 15.3290078 | 5.6024243 | 4.7151970 |
| H | 16.5663726 | 4.9295153 | 3.6407194 |
| H | 15.1200928 | 5.7056385 | 2.9656306 |
| C | 16.8858060 | 10.2014470 | 4.3224084 |
| C | 17.2470135 | 10.4541026 | 5.6463620 |
| H | 16.6148772 | 10.0951058 | 6.4484122 |
| C | 18.4076255 | 11.1594096 | 5.9324523 |
| H | 18.6805322 | 11.3492891 | 6.9628002 |
| C | 19.2168578 | 11.6207535 | 4.8980988 |
| H | 20.1219737 | 12.1714537 | 5.1210857 |
| C | 18.8593542 | 11.3723931 | 3.5776530 |
| H | 19.4841214 | 11.7293386 | 2.7686351 |
| C | 17.6978537 | 10.6639932 | 3.2897702 |
| H | 17.4222393 | 10.4718460 | 2.2616542 |
| C | 10.7727034 | 2.9914794 | 4.9538268 |
| H | 10.9157668 | 3.3998904 | 5.9552814 |
| H | 11.2762131 | 2.0239419 | 4.9050280 |

| | | | |
|---|---|---|---|
| H | 9.7093784 | 2.8200565 | 4.8007692 |
| C | 11.0396309 | 3.4087635 | 2.4819726 |
| H | 9.9614079 | 3.3376589 | 2.3385725 |
| H | 11.4666170 | 2.4153199 | 2.3263427 |
| H | 11.4490519 | 4.0873211 | 1.7332926 |
| C | 12.4585315 | 11.3469392 | 3.7664692 |
| H | 12.0695736 | 10.6578711 | 3.0168920 |
| H | 12.0759042 | 12.3477631 | 3.5592231 |
| H | 12.0941524 | 11.0332062 | 4.7428348 |
| C | 7.8882894 | 7.9979961 | 2.7509686 |
| H | 8.5048752 | 7.9196793 | 1.8541923 |
| H | 7.2136699 | 8.8488546 | 2.6334061 |
| H | 7.2797871 | 7.0990765 | 2.8376167 |
| C | 14.4494160 | 11.8239421 | 2.3425507 |
| H | 15.5354699 | 11.8904571 | 2.2865970 |
| H | 14.0416640 | 12.8136738 | 2.1250583 |
| H | 14.0996891 | 11.1282857 | 1.5789777 |
| C | 7.9308167 | 8.2328411 | 5.2647336 |
| H | 7.3216693 | 7.3376213 | 5.3740356 |
| H | 7.2592974 | 9.0934488 | 5.2351502 |
| H | 8.5696137 | 8.3284945 | 6.1446159 |
| C | 11.4673457 | 6.4986672 | 7.1943099 |
| H | 10.4575006 | 6.7359540 | 6.8534812 |
| H | 11.4760482 | 6.5452963 | 8.2864926 |
| H | 11.6908812 | 5.4740975 | 6.8934677 |
| C | 14.5143278 | 12.3011519 | 4.8184453 |
| H | 14.2673298 | 11.9253333 | 5.8126408 |
| H | 14.0437067 | 13.2791239 | 4.6984989 |
| H | 15.5918526 | 12.4411052 | 4.7603473 |
| C | 12.6901186 | 6.2385696 | 0.0743398 |
| H | 12.7958726 | 5.1625609 | 0.0822202 |
| C | 11.4044665 | 6.8316070 | 0.1304015 |
| H | 10.5322714 | 6.2007290 | 0.2388828 |
| C | 13.8319304 | 7.0654384 | 0.0300590 |
| H | 14.8202797 | 6.6271107 | 0.0141815 |
| C | 11.2525789 | 8.2400983 | 0.1603613 |
| H | 10.2671088 | 8.6769655 | 0.2474314 |
| C | 13.6800337 | 8.4737352 | 0.0494031 |
| H | 14.5599670 | 9.1015127 | 0.0941554 |
| C | 12.3951817 | 9.0670122 | 0.1237092 |
| H | 12.2929862 | 10.1425853 | 0.1721426 |
| C | 17.2741571 | 7.2562950 | 2.3869252 |
| H | 16.5285379 | 7.2833709 | 1.5915828 |
| H | 17.9494249 | 6.4176480 | 2.2030913 |

| | | | |
|---|---|---|---|
| H | 17.8617819 | 8.1727524 | 2.3495639 |
| C | 17.6192019 | 7.1222016 | 4.8815286 |
| H | 18.2334571 | 8.0200131 | 4.8535051 |
| H | 18.2835551 | 6.2614980 | 4.7802838 |
| H | 17.1316109 | 7.0635898 | 5.8549413 |
| C | 14.4145182 | 7.3362172 | 7.1654150 |
| H | 14.7350982 | 6.3232103 | 6.9120316 |
| H | 14.4227560 | 7.4299683 | 8.2549606 |
| H | 15.1564051 | 8.0269146 | 6.7604424 |
| C | 12.2406517 | 9.4318474 | 7.1122475 |
| H | 12.9540370 | 10.1838528 | 6.7694316 |
| H | 12.2389762 | 9.4473150 | 8.2061218 |
| H | 11.2468251 | 9.7346047 | 6.7743653 |

$N_2$

2

E= -109,53461

| | | | |
|---|---|---|---|
| N | 0.0000000 | 0.0000000 | 0.9805221 |
| N | 0.0000000 | 0.0000000 | -0.1105221 |

| | | |
|---|---|---|
| SM | -3463,66282 | |
| azide | -573,33931 | |
| combined | -4037,00213 | 0,0000 |
| FeSi2TS1 | -4036,97761 | 15,3865 |
| FeSi2TMSN3-1 | -4037,07239 | -59,4754 |
| FeSi2TMSN3-TS2-1 | -4037,03353 | 24,3850 |
| 4_ring | -4037,03454 | -0,6338 |
| 4_ring-TS3 | -4037,02559 | 5,6162 |
| FeSi2NTMS | -3927,6511 | |
| N2 | -109,53461 | |
| FeSi2NTMS+N2 | -4037,18571 | -94,8607 |

# Single crystal X-ray data diffraction

Table S1. X-ray data of $\{PhC(N^tBu)_2SiCl\}_2Fe(C_6H_6)$ (**2**). CCDC 2157512.

| | **$\{PhC(N^tBu)_2SiCl\}_2Fe(C_6H_6)$ (2)** |
|---|---|
| **chemical formula** | $C_{36}H_{52}Cl_2FeN_4Si_2$ |
| **fw** | 723.74 |
| ***T* (K)** | 300 |
| ***λ* (Å)** | 1.54178 |
| ***a* (Å)** | 9.7347(7) |
| ***b* (Å)** | 13.6577(8) |
| ***c* (Å)** | 18.8704(11) |
| ***α* (°)** | 99.845(4) |
| ***β* (°)** | 92.800(5) |
| ***γ* (°)** | 101.432(5) |
| ***V* (Å$^3$)** | 2413.99 |
| **space group** | *P* -1 |
| ***Z*, *Z'*** | 2, 1 |
| **$D_{calc}$ (g/cm$^3$)** | 0.996 |
| ***μ* (mm$^{-1}$)** | 4.174 |
| **R1 (I > 2σ(I)), wR2[a]** | 0.0840, 0.2672 |

[a] $R1 = \Sigma||F_o| - |F_c||/\Sigma|F_o|$, $wR2 = (\Sigma[w(F_o^2 - F_c^2)^2]/\Sigma[w(F_o^2)^2])c^{1/2}$

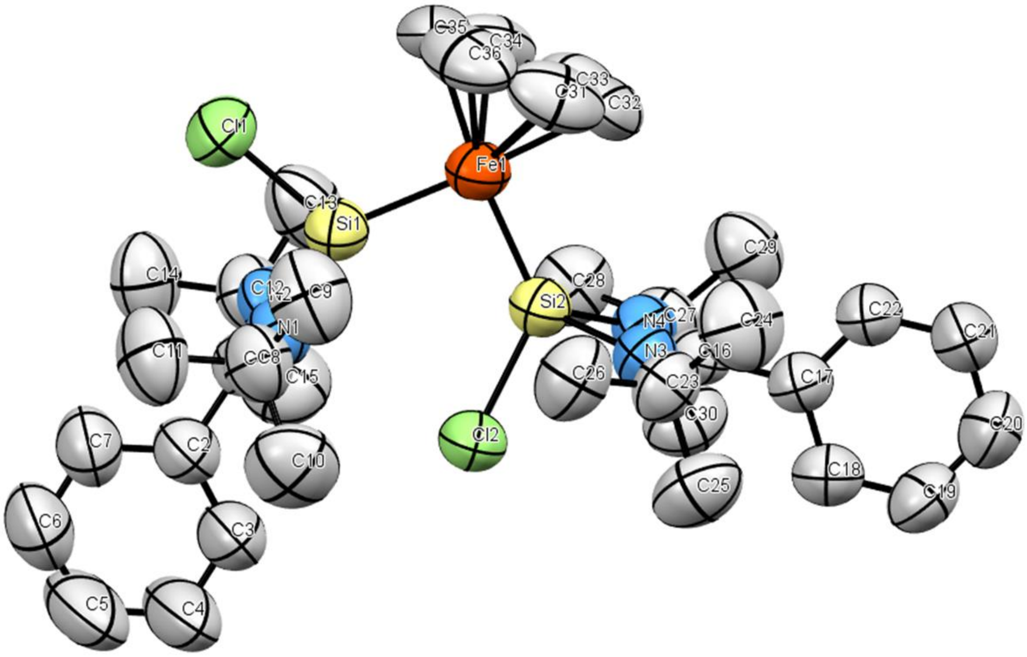


Figure S35. The full numbering scheme of $\{PhC(N^tBu)_2SiCl\}_2Fe(C_6H_6)$ (**2**). All atoms shown are depicted with 50% thermal contours. The hydrogen atoms have been removed for clarity.

Table S2. X-ray data of $\{PhC(N^tBu)_2Si\}_2Fe(C_6H_6)$ (**3**). CCDC 2157514.

| | **$\{PhC(N^tBu)_2Si\}_2Fe((C_6H_6)$ (3)** |
|---|---|
| **chemical formula** | $C_{36}H_{52}FeN_4Si_2$ |
| **fw** | 652.86 |
| ***T* (K)** | 150 |
| ***λ* (Å)** | 1.34138 |
| ***a* (Å)** | 21.1980(9) |
| ***b* (Å)** | 10.2965(5) |
| ***c* (Å)** | 15.8356(6) |
| ***α* (°)** | 90 |
| ***β* (°)** | 91.273(2) |
| ***γ* (°)** | 90 |
| ***V* (Å$^3$)** | 3455.51 |
| **space group** | *C 2/c* |
| ***Z, Z'*** | 4, 0.5 |
| **$D_{calc}$ (g/cm$^3$)** | 1.255 |
| ***μ* (mm$^{-1}$)** | 2.995 |
| **R1 (I > 2σ(I)), wR2[a]** | 0.0418, 0.1035 |

[a] $R1 = \Sigma||F_o| - |F_c||/\Sigma|F_o|$, $wR2 = (\Sigma[w(F_o^2 - F_c^2)^2]/\Sigma[w(F_o^2)^2])c^{1/2}$

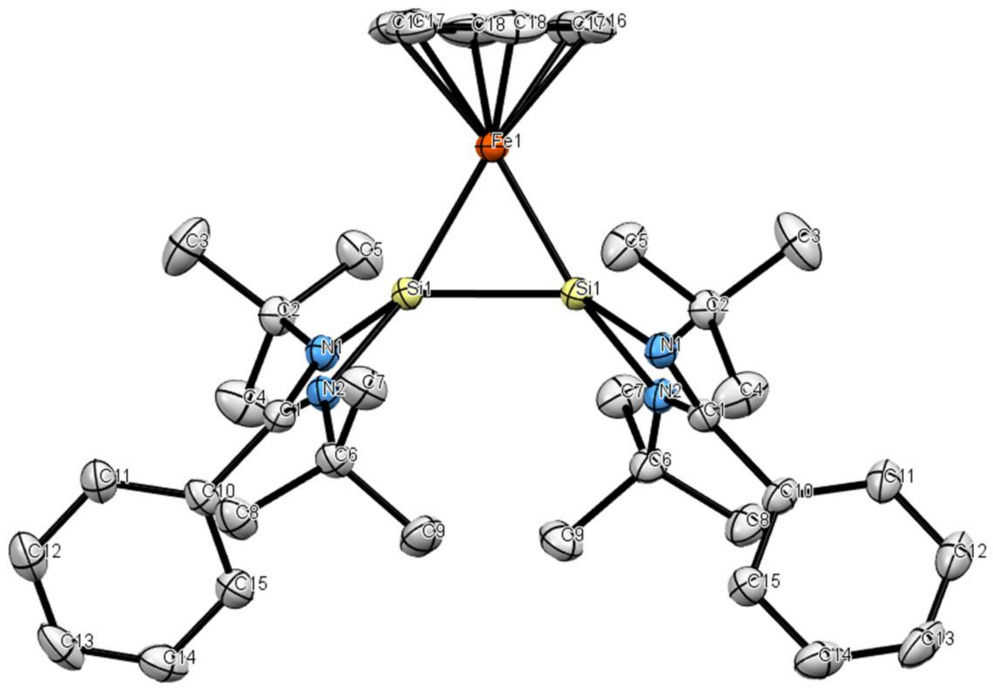


Figure S36. The full numbering scheme of $\{PhC(N^tBu)_2Si\}_2Fe(C_6H_6)$ (**3**). All atoms shown are depicted with 50% thermal contours. The hydrogen atoms have been removed for clarity.

Table S3. X-ray data of $\{PhC(N^tBu)_2Si\}_2Fe(C_6H_6)(N_3SiMe_3)$ (**4**). CCDC 2157515.

| | **$\{PhC(N^tBu)_2Si\}_2Fe((C_6H_6)(N_3SiMe_3)$ (4)** |
|---|---|
| **chemical formula** | $C_{39}H_{61}FeN_7Si_3$ |
| **fw** | 768.06 |
| ***T* (K)** | 150 |
| ***λ* (Å)** | 1.34138 |
| ***a* (Å)** | 9.8807(8) |
| ***b* (Å)** | 28.708(2) |
| ***c* (Å)** | 14.9418(13) |
| ***α* (°)** | 90 |
| ***β* (°)** | 92.442(5) |
| ***γ* (°)** | 90 |
| ***V* ($Å^3$)** | 4234.47 |
| **space group** | *P 2₁/n* |
| ***Z, Z'*** | 4, 1 |
| **$D_{calc}$ ($g/cm^3$)** | 1.205 |
| ***μ* ($mm^{-1}$)** | 2.676 |
| **R1 (I > 2σ(I)), wR2[a]** | 0.0515, 0.1454 |

[a] $R1 = \Sigma||F_o| - |F_c||/\Sigma|F_o|$, $wR2 = (\Sigma[w(F_o^2 - F_c^2)^2]/\Sigma[w(F_o^2)^2])c^{1/2}$

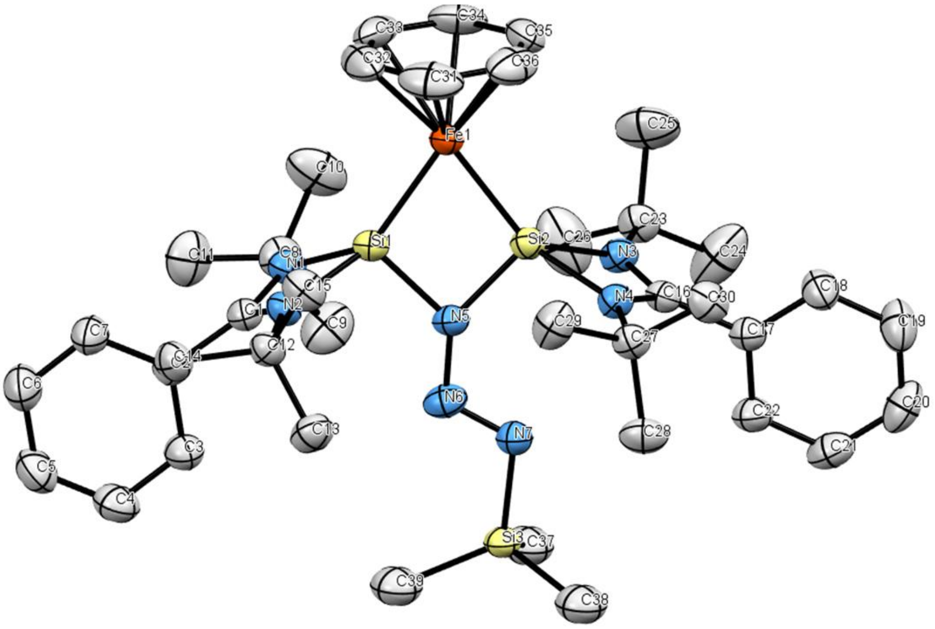


Figure S37. The full numbering scheme of $\{PhC(N^tBu)_2Si\}_2Fe(C_6H_6)(N_3SiMe_3)$ (**4**). All atoms shown are depicted with 50% thermal contours. The hydrogen atoms have been removed for clarity.

Table S4. X-ray data of $\{PhC(N^tBu)_2Si\}_2Fe(C_6H_6)(NSiMe_3)$ (**5**). CCDC 2157513.

| | **$\{PhC(N^tBu)_2Si\}_2Fe(C_6H_6)(NSiMe_3)$ (5)** |
|---|---|
| **chemical formula** | $C_{39}H_{61}FeN_5Si_3$ |
| **fw** | 740.05 |
| ***T* (K)** | 150 |
| ***λ* (Å)** | 1.34138 |
| ***a* (Å)** | 13.2544(10) |
| ***b* (Å)** | 13.2778(10) |
| ***c* (Å)** | 16.7537(13) |
| ***α* (°)** | 76.065(3) |
| ***β* (°)** | 89.450(3) |
| ***γ* (°)** | 62.243(2) |
| ***V* ($Å^3$)** | 2514.29 |
| **space group** | *P* -1 |
| ***Z, Z'*** | 2, 1 |
| **$D_{calc}$ ($g/cm^3$)** | 0.978 |
| ***μ* ($mm^{-1}$)** | 2.187 |
| **R1 (I > 2σ(I)), wR2[a]** | 0.0833, 0.2473 |

[a] $R1 = \Sigma||F_o| - |F_c||/\Sigma|F_o|$, $wR2 = (\Sigma[w(F_o^2 - F_c^2)^2]/\Sigma[w(F_o^2)^2])c^{1/2}$

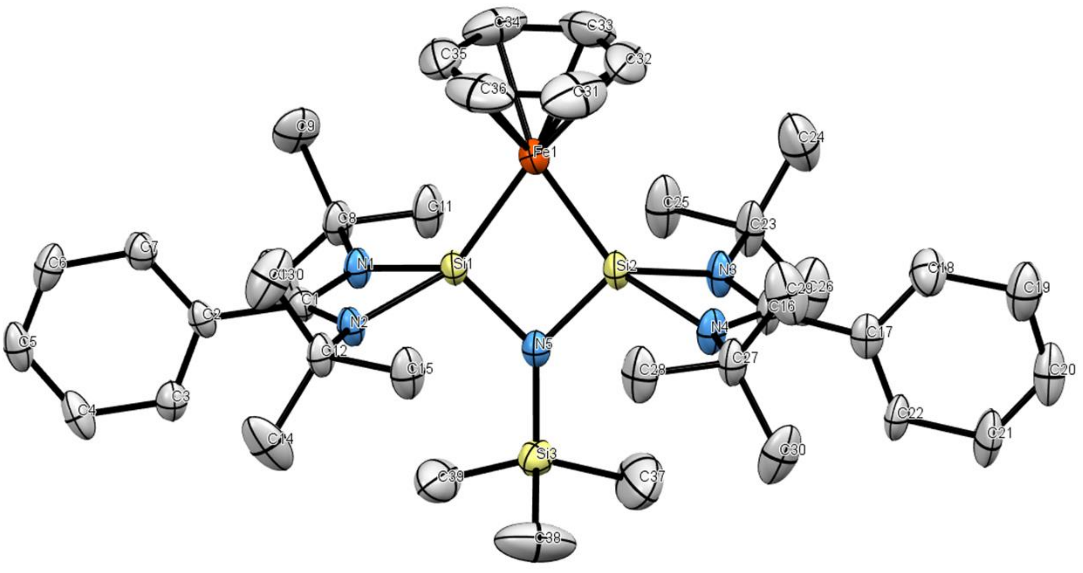


Figure S38. The full numbering scheme of $\{PhC(N^tBu)_2Si\}_2Fe(C_6H_6)(NSiMe_3)$ (**5**). All atoms shown are depicted with 50% thermal contours. The hydrogen atoms have been removed for clarity.

Table S5. X-ray data of {PhC(N$^t$Bu)$_2$Si}$_2$Fe(C$_6$H$_6$))(Ph$_2$O) (**6**). CCDC 2157516.

| | **{PhC(N$^t$Bu)$_2$Si}$_2$Fe(C$_6$H$_6$)(Ph$_2$O) (6)** |
|---|---|
| **chemical formula** | $C_{49}H_{62}FeN_4OSi_2$ |
| **fw** | 835.05 |
| ***T*** **(K)** | 150 |
| ***λ*** **(Å)** | 1.34138 |
| ***a*** **(Å)** | 13.0002(10) |
| ***b*** **(Å)** | 13.7708(9) |
| ***c*** **(Å)** | 14.3396(11) |
| ***α*** **(°)** | 86.224(3) |
| ***β*** **(°)** | 68.025(3) |
| ***γ*** **(°)** | 70.234(3) |
| ***V*** **(Å$^3$)** | 2234.93 |
| **space group** | *P* -1 |
| ***Z, Z'*** | 2, 1 |
| **$D_{calc}$ (g/cm$^3$)** | 1.241 |
| ***μ*** **(mm$^{-1}$)** | 2.347 |
| **R1 (I > 2σ(I)), wR2$^a$** | 0.0537, 0.1579 |

$^a$ R1 = $\Sigma||F_o| - |F_c||/\Sigma|F_o|$, wR2 = $(\Sigma[w(F_o^2 - F_c^2)^2]/\Sigma[w(F_o^2)^2])c^{1/2}$

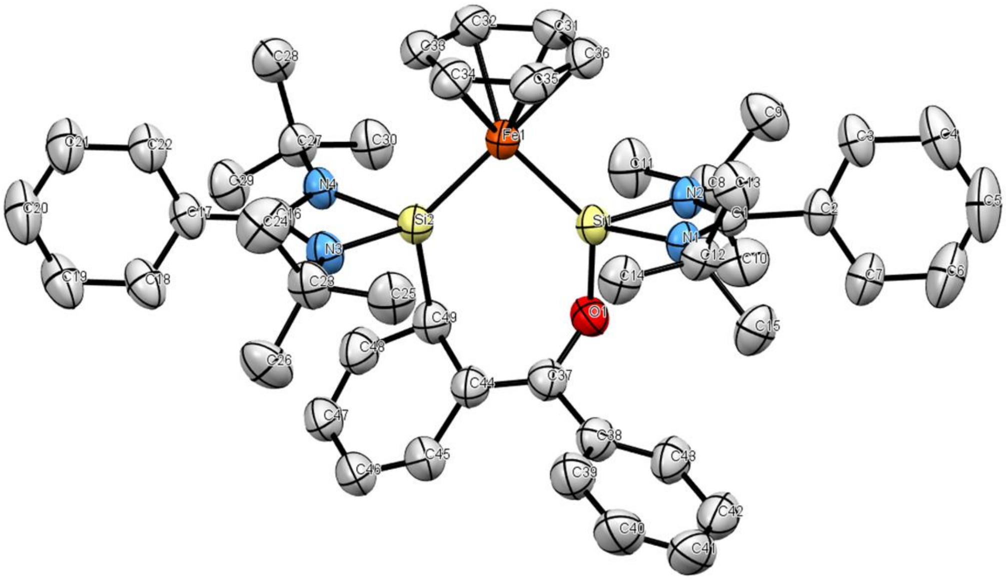


Figure S39. The full numbering scheme of {PhC(N$^t$Bu)$_2$Si}$_2$Fe(C$_6$H$_6$)(Ph$_2$O) (**6**). All atoms shown are depicted with 50% thermal contours. The hydrogen atoms have been removed for clarity.

## Supplementary References


[S1] Sen, S. S.; Roesky, H. W.; Stern, D.; Henn, J.; Stalke, D. High Yield Access to Silylene RSiCl (R = PhC(NtBu)$_2$) and Its Reactivity toward Alkyne: Synthesis of Stable Disilacyclobutene. *J. Am. Chem. Soc.* **2010**, *132*, 1123–1126.

[S2] Broere, D. L. J.; Coric, I.; Brosnahan, A.; Holland, P. L. Quantitation of the THF Content in Fe[N(SiMe$_3$)$_2$]$_2$·*x*THF. *Inorg. Chem.* **2017**, *56*, 3140–3143.

[S3] He, Z.; Xue, X.; Liu, Y.; Yu, N.; Krogman, J. P. Aminolysis of bis[bis(trimethylsilyl)amido]-manganese, -iron, and -cobalt for the synthesis of mono- and bis-silylene complexes. *Dalton Trans.* **2020**, *49*, 12586–12591.

[S4] Dolomanov, O. V.; Bourhis, L. J.; Gildea, R. J.; Howard, J. A. K.; Puschmann, H. *J. Appl. Cryst.* **2009**, *42*, 339–341.

[S5] Sheldrick, G. M. SHELXT–Integrated space-group and crystal-structure determination. *Acta Cryst. A* **2015**, *71*, 3–8.

[S6] Sheldrick, G. M. A short history of SHELX. *Acta Cryst. A* **2008**, *64*, 112–122.

[S7] TURBOMOLE, Version 7.5.1 (TURBOMOLE Gmbh, Karlsruhe, Germany).

[S8] a) PQS version 2.4, 2001, Parallel Quantum Solutions, Fayetteville, Arkansas, USA (the Baker optimizer is available separately from PQS upon request); (b) Baker, J. An algorithm for the location of transition states. *J. Comput. Chem.* **1986**, *7*, 385–395.

[S9] Budzelaar, P. H. M. Geometry optimization using generalized, chemically meaningful constraints. *J. Comput. Chem.* **2007**, *28*, 2226–2236.

[S10] Becke, A. D. Density-functional thermochemistry. III. The role of exact exchange. *J. Chem. Phys.* **1993**, *98*, 5648-5652; (b) Lee, C.; Yang, W.; Parr, R. G. Development of the Colle-Salvetti correlation-energy formula into a functional of the electron density. *Phys. Rev. B* **1988**, *37*, 785–789.

[S11] (a) Weigend, F.; Ahlrichs, R. Balanced basis sets of split valence, triple zeta valence and quadruple zeta valence quality for H to Rn: Design and assessment of accuracy. *Phys. Chem. Chem. Phys.* **2005**, *7*, 3297–3305; (b) Weigend, F.; Häser, M.; Patzelt, H.; Ahlrichs, R. RI-MP2: optimized auxiliary basis sets and demonstration of efficiency. *Chem. Phys. Lett*. **1998**, *294*, 143–152.

[S12] Grimme, S.; Antony, J.; Ehrlich, S.; Krieg, H. A consistent and accurate ab initio parametrization of density functional dispersion correction (DFT-D) for the 94 elements H-Pu. *J. Chem. Phys*. **2010**, *132*, 154104.

[S13] EDA is very informative for symmetrical structures with the orbital interactions identifiable in different irreducible representations. See: Ziegler, T.; Rauk, A. On the calculation of bonding energies by the Hartree Fock Slater method. *Theor. Chim. Acta* **1977,** *1*, 46.

[S14] a) van Lenthe, E.; Baerends, E. J. Optimized Slater-type basis sets for the elements 1–118. *J. Comput. Chem.* **2003**, *24*, 1142–1156; b) Becke, A. D. *Phys. Rev. A* **1988**, *38*, 3098–3100; c) Perdew, J. P.; Yue, W. Accurate and simple density functional for the electronic exchange energy: Generalized gradient approximation. *Phys. Rev. B* **1986**, *33*, 8800–8802.

[S15] a) von Ragué Schleyer, P.; Maerker, C.; Dransfeld, A.; Jiao, H. J.; van Eikema Hommes, N. J. R. Nucleus-Independent Chemical Shifts: A Simple and Efficient Aromaticity Probe. *J. Am. Chem. Soc.* **1996**, *118*, 6317–6318; b) von Ragué Schleyer, P.; Manoharan, M.; Wang, Z. X.; Kiran, B.; Jiao, H. J.; Puchta, R.; van Eikema Hommes, N. J. R. Dissected Nucleus-Independent Chemical Shift Analysis of π-Aromaticity and Antiaromaticity. *Org. Lett.* **2001**, *3*, 2465–2468; c) Chen, Z. F.; Wannere, C. S.; Corminboeuf, C.; Puchta, R.; von Ragué Schleyer, P. Nucleus-Independent Chemical Shifts (NICS) as an Aromaticity Criterion. *Chem. Rev.* **2005**, *105*, 3842–3888.

[S16] Gaussian: Gaussian 16, Revision A.03, Frisch, M. J.; Trucks, G. W.; Schlegel, H. B.; Scuseria, G. E.; Robb, M. A.; Cheeseman, J. R.; Scalmani, G.; Barone, V.; Petersson, G. A.; Nakatsuji, H.; Li, X.; Caricato, M.; Marenich, A. V.; Bloino, J.; Janesko, B. G.; Gomperts, R.; Mennucci, B.; Hratchian, H. P.; Ortiz, J. V.; Izmaylov, A. F.; Sonnenberg, J. L.; Williams-Young, D.; Ding, F.; Lipparini, F.; Egidi, F.; Goings, J.; Peng, B.; Petrone, A.; Henderson, T.; Ranasinghe, D.; Zakrzewski, V. G.; Gao, J.; Rega, N.; Zheng, G.; Liang, W.; Hada, M.; Ehara, M.; Toyota, K.; Fukuda, R.; Hasegawa, J.; Ishida, M.; Nakajima, T.; Honda, Y.; Kitao, O.; Nakai, H.; Vreven, T.; Throssell, K.; Montgomery, Jr., J. A.; Peralta, J. E.; Ogliaro, F.; Bearpark, M. J.; Heyd, J. J.; Brothers, E. N.; Kudin, K. N.; Staroverov, V. N.; Keith, T. A.; Kobayashi, R.; Normand, J.; Raghavachari, K.; Rendell, A. P.; Burant, J. C.; Iyengar, S. S.; Tomasi, J.; Cossi, M.; Millam, J. M.; Klene, M.; Adamo, C.; Cammi, R.; Ochterski, J. W.; Martin, R. L.; Morokuma, K.; Farkas, O.; Foresman, J. B.; Fox, D. J. Gaussian, Inc., Wallingford CT, 2016.

[S17] a) McLean, A. D.; Chandler, G. S. Contracted Gaussian basis sets for molecular calculations. I. Second row atoms, *Z*=11–18. *J. Chem. Phys.* **1980**, *72*, 5639; b) Krishnan, R.; Binkley, J. S.; Seeger, R.; Pople, J. A. Self-consistent molecular orbital methods. XX. A basis set for correlated wave functions. *J. Chem. Phys.* **1980**, *72*, 650.

[S18] Klod, S.; Kleinpeter, E. Ab initio calculation of the anisotropy effect of multiple bonds and the ring current effect of arenes—application in conformational and configurational analysis. *J. Chem. Soc., Perkin Trans. 2* **2001**, 1893–1898.

[S19] Tsipis, A. C.; Depastas, I. G.; Tsipis, C. A. Diagnosis of the σ-, π- and (σ+π)-Aromaticity by the Shape of the $NICS_{zz}$-Scan Curves and Symmetry-Based Selection Rules. *Symmetry* **2010**, *2*, 284–319.

[S20] a) Fallah-Bagher-Shaidaei, H.; Wannere, C. S.; Corminboeuf, C.; Puchta, R.; von Ragué Schleyer, P. Which NICS Aromaticity Index for Planar π Rings Is Best? *Org. Lett.* **2006**, *8*, 863–866; b) Wodrich, M. D.; Corminboeuf, C.; Park, S. S.; von Ragué Schleyer, P. Double Aromaticity in Monocyclic Carbon, Boron, and Borocarbon Rings Based on Magnetic Criteria. *Chem. Eur. J.* **2007**, *13*, 4582–4593.

[S21] a) Bohmann, J. A.; Weinhold, F.; Farrar, T. C. Natural chemical shielding analysis of nuclear magnetic resonance shielding tensors from gauge-

including atomic orbital calculations. *J. Chem. Phys.* **1997**, *107*, 1173–1184; b) Heine, T.; von Ragué Schleyer, P.; Corminboeuf, C.; Seifert, G.; Reviakine, R.; Weber, J. Analysis of Aromatic Delocalization: Individual Molecular Orbital Contributions to Nucleus-Independent Chemical Shifts. *J. Phys. Chem. A* **2003**, *107*, 6470–6475; c) Corminboeuf, C.; Heine, T.; Weber, J. Evaluation of aromaticity: A new dissected NICS model based on canonical orbitals. *Phys. Chem. Chem. Phys.* **2003**, *5*, 246–251.

[S22] a) Jimenez-Halla, J. O. C.; Matito, E.; Robles, J.; Sola, M. Nucleus-independent chemical shift (NICS) profiles in a series of monocyclic planar inorganic compounds. *J. Organomet. Chem.* **2006**, *691*, 4359–4366; b) Poater, J.; Bofill, J. M.; Alemany, P.; Sola, M. Role of Electron Density and Magnetic Couplings on the Nucleus-Independent Chemical Shift (NICS) Profiles of [2.2]Paracyclophane and Related Species. *J. Org. Chem.* **2006**, *71*, 1700–1702.

[S23] a) Stanger, A. Can Substituted Cyclopentadiene Become Aromatic or Antiaromatic? *Chem. Eur. J.* **2006,** *12,* 2745–2751; b) Stanger, A. Nucleus-Independent Chemical Shifts (NICS): Distance Dependence and Revised Criteria for Aromaticity and Antiaromaticity. *J. Org. Chem.* **2006**, *71*, 883–893.
